# Synthesis of Gentamicins C1, C2, and C2a and Antiribosomal and Antibacterial Activity of Gentamicins B1, C1, C1a, C2, C2a, C2b, and X2

**DOI:** 10.1021/acsinfecdis.3c00233

**Published:** 2023-07-23

**Authors:** Santanu Jana, Parasuraman Rajasekaran, Klara Haldimann, Andrea Vasella, Erik C. Böttger, Sven N. Hobbie, David Crich

**Affiliations:** † Department of Pharmaceutical and Biomedical Sciences, University of Georgia, 250 West Green Street, Athens, Georgia 30602, United States; ‡ Complex Carbohydrate Research Center, University of Georgia, 315 Riverbend Road, Athens, Georgia 30602, United States; § Institute of Medical Microbiology, University of Zurich, Gloriastrasse 30, 8006 Zürich, Switzerland; ∥ Organic Chemistry Laboratory, ETH Zürich, Vladimir-Prelog-Weg 1-5/10, 8093 Zürich, Switzerland; ⊥ Department of Chemistry, University of Georgia, 302 East Campus Road, Athens, Georgia 30602, United States

**Keywords:** gentamicins, mitochondrial and cytoplasmic ribosomes, antibacterial resistance, ototoxicity

## Abstract

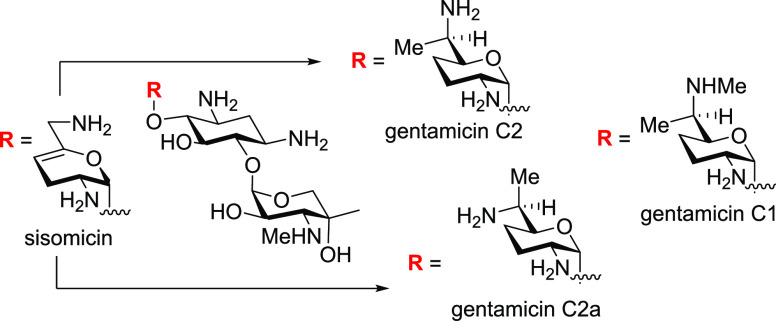

Complementing our earlier syntheses of the gentamicins B1, C1a, C2b, and X2, we describe the synthesis of gentamicins C1, C2, and C2a characterized by methyl substitution at the 6′-position, and so present an alternative access to previous chromatographic methods for accessing these sought-after compounds. We describe the antiribosomal activity of our full set of synthetic gentamicin congeners against bacterial ribosomes and hybrid ribosomes carrying the decoding A site of the human mitochondrial, A1555G mutant mitochondrial, and cytoplasmic ribosomes and establish structure–activity relationships with the substitution pattern around ring I to antiribosomal activity, antibacterial resistance due to the presence of aminoglycoside acetyl transferases acting on the 6′-position in ring I, and literature cochlear toxicity data.

Gentamicin is a clinically important 4,6-disubstituted-2-deoxystreptamine class aminoglycoside antibiotic (2-DOS AGA) and a member of the World Health Organization’s Essential Medicines List.^1^ Its use for the treatment of Gram-negative infections in a hospital setting is impaired by widespread resistance arising from the presence of aminoglycoside modifying enzymes (AMEs),^2−5^ or ribosomal methyltransferases (RMTs),^6−8^ and increasingly combinations of one or both mechanisms with resistance to carbapenem antibiotics.^9,10^ Gentamicin therapy is also marred by the side effects of nephrotoxicity and drug-induced hearing loss or ototoxicity.^11−14^ Unfortunately, while nephrotoxicity is adequately managed by use of a once daily dosing regimen for no more than 10 days, ototoxicity affects up to twenty percent of patients, and a significantly greater percentage of genetically hypersusceptible ones, is not monitored in the clinic, and is typically not apparent before discharge. Gentamicin is produced by fermentation from *Micromonospora* species^15^ as a mixture of multiple components,^16^ whose exact composition varies according to source but consists mainly of gentamicins C1, C1a, C2, C2a, and C2b along with minor amounts of gentamicins A, B, B1, and X2, and of sisomicin, 2-deoxystreptamine, garamine, and garosamine (Figure 1).^17−21^

**Figure 1:**
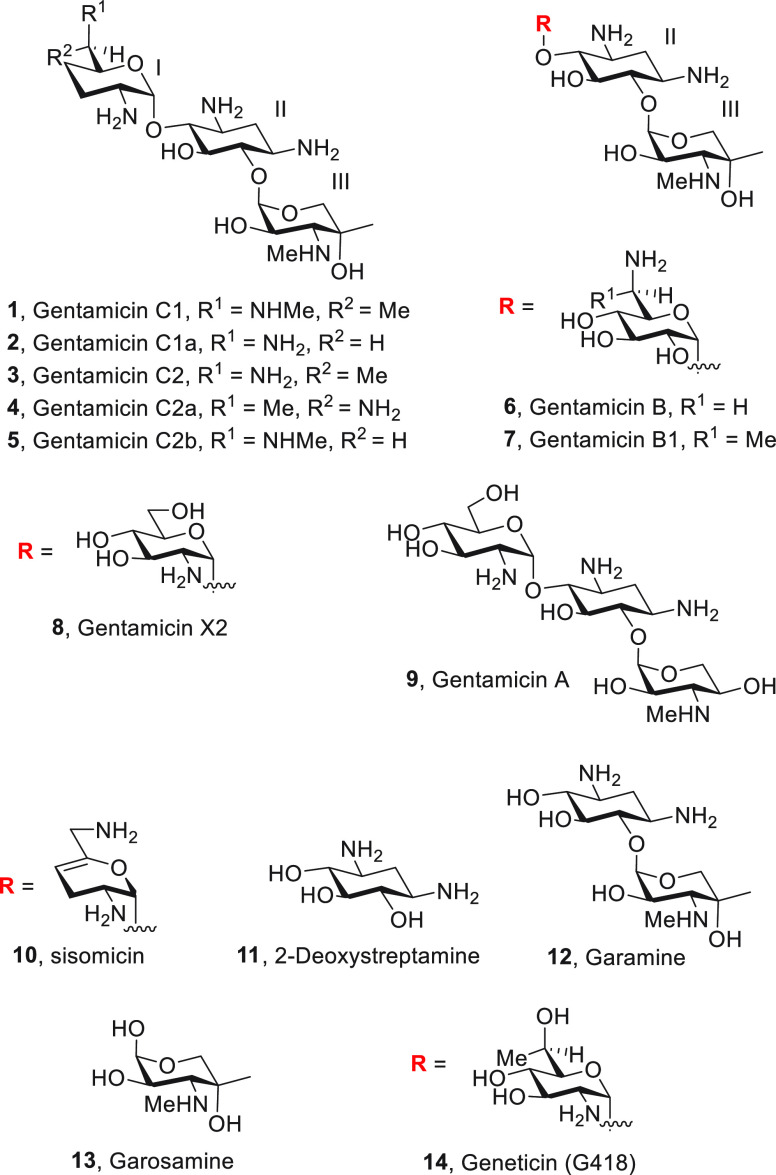
Components of the gentamicin mixture and geneticin (G418).

Beginning in the 1970s,^22−24^ multiple authors have studied the relative activity and toxicity of the individual components of the commercial mixtures leading to suggestions, for example, (i) that gentamicin C2 **3** is less nephrotoxic than the other congeners and^25^ (ii) that gentamicins C1 **1** and C1a **2** are less ototoxic than C2.^26^ More recent work from Ishikawa and workers, using mouse cochlear explants to evaluate ototoxicity, indicated gentamicin C1a (**2**) to be less ototoxic than a commercial gentamicin mixture,^27^ while similar but more extensive work from Cheng and coworkers, using rat cochlear explants, has determined that among the C subtype of gentamicins, C2b **5** is the least and C2 **3** the most ototoxic.^28^ At the same time, it was determined through standard MIC assays that all C subtypes evaluated had comparable antibacterial activity leading to the suggestion that gentamicin C2b **5** is the optimal congener.^28^ A contemporaneous study by Andrews and coworkers concluded that while gentamicins C1, C1a, C2, and C2a all had comparable activities against wild-type Gram negative pathogens, C2 **3** was more nephrotoxic than C1a **2** and C2a **4**, with C1 not isolated in sufficient quantity to assay toxicity.^29^ Building on early observations with geneticin (G418) **14**, other groups have studied aminoglycosides for improved read-through properties in diseases arising from the presence of premature termination codons.^30−34^ These studies led to (i) a report of the superior properties of gentamicin B1 **7** as a read-through agent that was subsequently retracted when it was found that the commercial sample employed was mislabeled geneticin **14**, with actual gentamicin B1 showing no such activity,^35^ and (ii) the description of gentamicin X2 **8** as a compound with read-through properties better than those of geneticin **14**.^36^

Not surprisingly, these efforts to characterize structure activity relationships and separate activity from toxicity among the difficult-to-separate components of the commercial gentamicin mixtures have spurred efforts for the preparation of single components. Such efforts have taken the path of either understanding and manipulating the biosynthetic pathways from sisomicin^16,37−39^ or of chemical synthesis.^40−43^ Following the latter path, in our laboratory, we have described syntheses of gentamicins B1 and X2 from sisomicin by a route involving ring I cleavage and reglycosylation^44^ and of gentamicins C1a and C2b by manipulation of the sisomicin ring I.^45^

The antibacterial activity of aminoglycosides derives from their well-known ability to bind to the decoding A site of the bacterial ribosome and their consequent inhibition of bacterial protein synthesis.^12,46−50^ One hypothesis for the ototoxic effects of aminoglycosides revolves around their ability to bind and inhibit the decoding A site of human mitochondrial ribosomes and especially the A1555G mutant that characterizes patients hypersensitive to AGA ototoxicity.^51−55^ A second, and not necessarily mutually exclusive, hypothesis of AGA ototoxicity centers on their uptake into cochlear hair cells by mechanotransducer (MET) channels.^56−58^ Both hypotheses provide the opportunity for compound optimization and have been tested experimentally with positive results in both cases.^59−63^ The potential of aminoglycosides to serve as therapeutics for read-through diseases similarly derives from their ability to bind and inhibit ribosomes, but this time human cytoplasmic ribosomes.^64−66^

Extrapolating from our syntheses of gentamicins C1a and C2b, we now describe the synthesis of gentamicins C1, C2, and C2a again from sisomicin and then, taking advantage of the availability of pure samples from our synthesis program, report on the relative inhibitory abilities of gentamicins B1, C1, C1a, C2, C2a, C2b, and X2 toward the bacterial ribosome and humanized hybrid bacterial ribosomes bearing the complete decoding A site of human mitochondrial, human A1555G mutant mitochondrial, and human cytoplasmic ribosomes using a series of cell-free translation assays.^67,68^

## Results and Discussion

### Synthesis

**Scheme 1:**
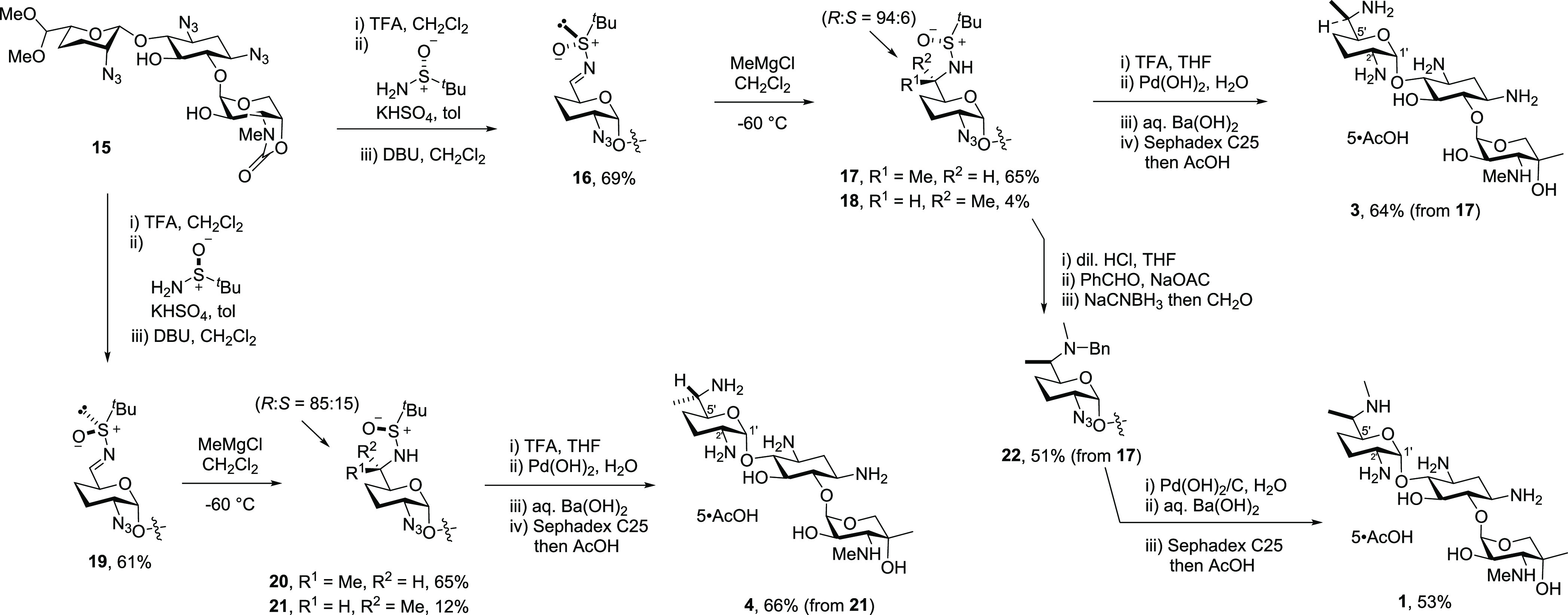
Synthesis of Gentamicins C2 (**3**), C2a (**4**), and C1 (**1**)

Our synthesis of gentamicins C1 **1**, C2 **3**, and C2a **4** began with sisomicin **10** that was converted to the key intermediate **15** in seven steps as described previously.^45^ Cleavage of the acetal with trifluoroacetic acid in wet dichloromethane and subsequent exposure to (*R*)-*tert*-butylsulfinamide and potassium hydrogen sulfate in toluene at 40 °C gave a sulfinyl imine that, without purification, was stirred with diazabicycloundecene in dichloromethane at 40 °C, resulting in epimerization at C5′ and inversion of conformation to obtain **16** in 69% yield over three steps. Addition of methylmagnesium chloride in dichloromethane at −60 °C^69,70^ then gave a 94:6 mixture of two 6′-C-methylated compounds, retrospectively assigned as **17** and **18**, which were isolated after chromatographic purification in 65% and 4% yield, respectively. Finally, acidic hydrolysis of sulfinamide **17**, hydrogenolysis over palladium hydroxide on carbon, and treatment with hot aqueous barium hydroxide gave gentamicin C2 **3** in 64% yield in the form of its acetate salt after chromatography over Sephadex C-25 and lyophilization from aqueous acetic acid (Scheme 1).

Repeating the sequence from **15** with (*S*)-*tert*-butylsulfinamide gave compound **19** in 61% yield, which on treatment with methyl magnesium chloride in dichloromethane at −60 °C gave an 85:15 mixture of two 6′-C-methylated compounds **20** and **21**, which were isolated by column chromatography in 65% and 12% yield, respectively, and assigned the indicated relative configurations following deprotection. The minor isomer **21** was deprotected by exposure to trifluoroacetic acid followed by hydrogenolysis and eventual removal of the oxazolidinone with aqueous barium hydroxide to give gentamicin C2a **4** in 66% yield (Scheme 1). Treatment of sulfinamide **17** with hydrochloric acid in tetrahydrofuran gave an amine that was condensed with benzaldehyde to give an imine that was reduced with excess sodium cyanoborohydride followed by addition of formaldehyde and further reduction to afford the tertiary amine **22** in 51% yield over three steps. Hydrogenolysis of **22** followed by heating with aqueous barium hydroxide then provided gentamicin C1 **1** in 53% yield (Scheme 1).

### Assignment of Configuration of **3** and **4** and Their Precursors

**Figure 2:**
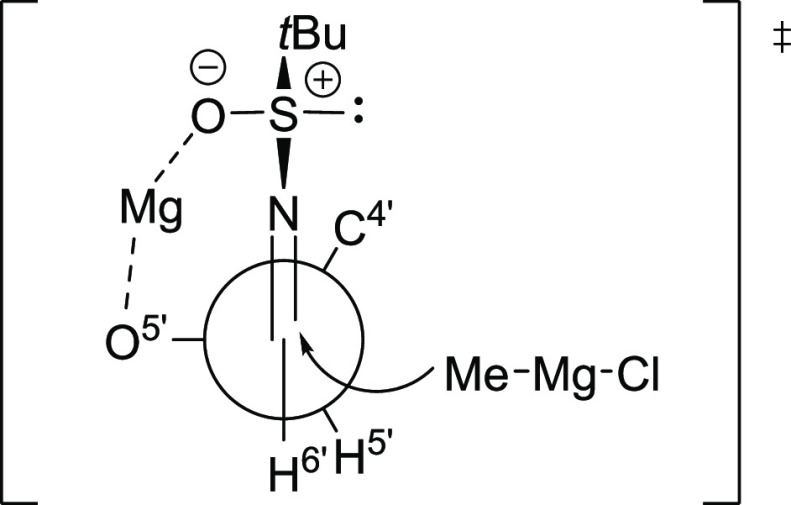
Chelation-controlled open-transition state with Felkin–Anh control for the selective formation of **17** from **16.**

The NMR spectra of gentamicins C1 **1**, C2 **3**, and C2a **4** obtained in this manner are fully consistent with the assigned structures and serve to confirm the original assignments by workers at Schering Corporation.^71^ In particular, the anomeric proton of ring I of the acetate salts resonates at δ 5.73–5.79 in the form of a doublet with a vicinal coupling constant of 3.6 Hz, while H2′ has a chemical shift of δ 3.54 and is an apparent doublet of triplets with coupling constants of 12.3–12.8 and 3.6–4.3 Hz, and H5′ appears at δ 4.21 as either an apparent doublet of triplets or a doublet of doublet of doublets with a ^3^*J*_H4’ax,H5’_ of between 11.8 and 12.3 Hz, indicating an overall ^4^*C*_1_ conformation of ring I with two equatorial substituents and an axial glycosidic bond. The ^1^H and ^13^C NMR data for **1**, **3**, and **4** are also consistent with those reported by Holzgrabe and co-workers recorded with samples isolated by preparative HPLC,^17^ albeit minor differences in chemical shift exist because of the different salts employed. Notably, the distinguishing C6′-methyl group in gentamicin C2 **3** (δ_H_ 1.30, d, *J* = 6.9 Hz) is upfield of that for its epimer gentamicin C2a **4** (δ_H_ 1.33, d, *J* = 6.8 Hz), while that for gentamicin C1 appears at δ_H_ 1.31 (d, *J* = 6.9 Hz); the ^13^C chemical shifts for this methyl group (δ_C_ 12.7, 14.4, and 10.1 for **3**, **4**, and **1**, respectively) also follow the literature pattern. Backtracking from the assignment of **3** as gentamicin C2 and of **4** as gentamicin C2a gives the indicated configurations in the diastereomeric pairs **17** and **18** and **20** and **21** and leads to the conclusion that the outcome of these reactions is dominated by substrate control employing the Felkin–Anh model^72^ onto which is layered the influence of the sulfinamide auxiliary with assistance from chelation to the magnesium counter-ion. The observed selectivities are not consistent with the chair-like six-membered cyclic transition state proposed by Ellman and coworkers for Grignard reagent addition to simple *tert*-butylsulfinyl imines in dichloromethane solution.^69^ In the case of the *R*-sulfinyl imine **16**, stereochemical matching^73,74^ between the chiral auxiliary and the substrate is observed leading to high selectivity for the formation of **17**, possibly via an open transition state such as depicted in Figure 2, whereas the lower selectivity in addition to the *S*-sulfinyl imine **19** is the result of stereochemical mismatching. The substrate-directed addition to **16** and **19** stands in contrast to the reaction of a sisomicin derived 4′,5′-unsaturated aldehyde with methylmagnesium bromide reported by Hanessian and coworkers in their synthesis of the vedamicins C2 and C2a when a 1:1 mixture of isomers was obtained.^75^

### Side Chain Conformation of Gentamicins C1 (**1**), C (**3**), C2a (**4**), and B1 (**7**)

**Figure 3:**
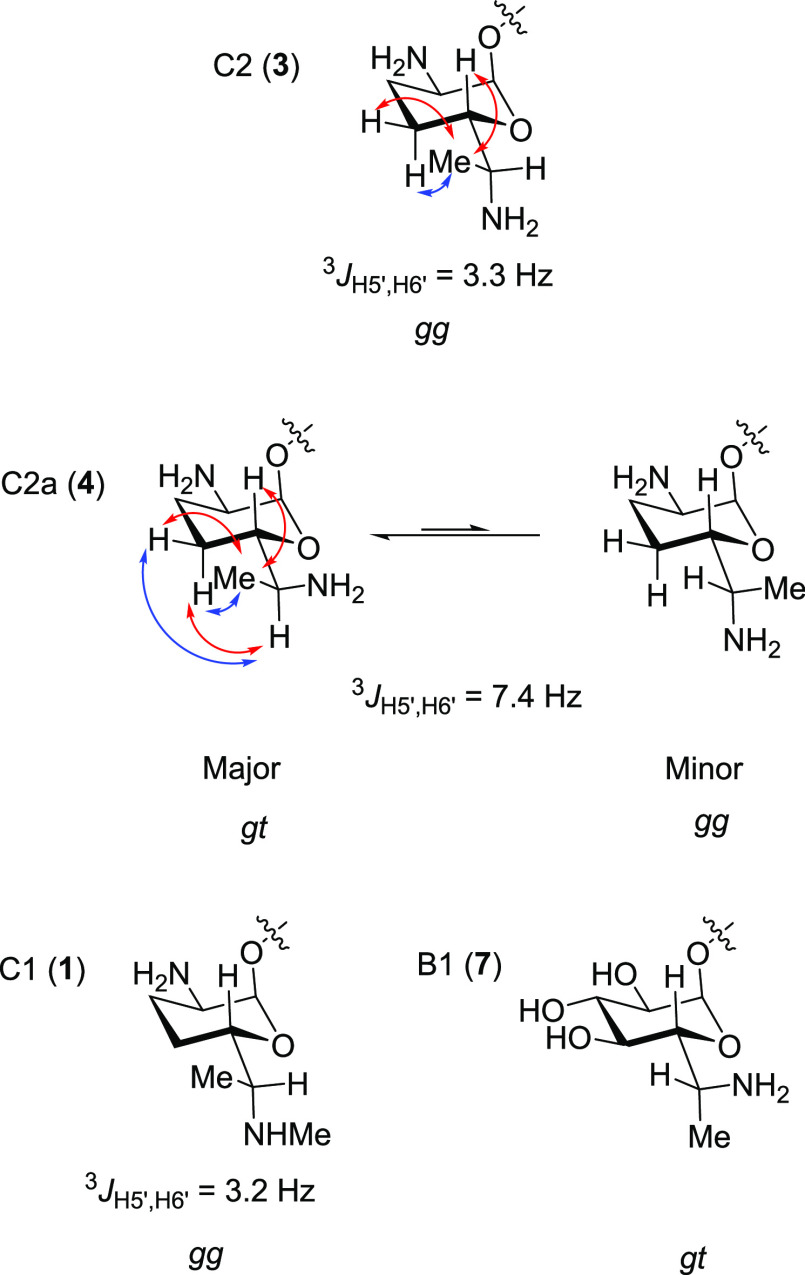
Diagnostic coupling constants, major (red) and minor (blue) ROESY interactions, and predominant side chain conformations of C2 (**3**) and C2a (**4**) in D_2_O at pD5, and predominant side chain conformations of C1 (**1**) and B1 (**7**) assigned by analogy with C2 and with the neomycin series, respectively.

In hexopyranosides and aminoglycosides, the inclusion of methyl or a larger substituent at C6 in the side chain constrains rotation about the exocyclic side chain bond and typically results in a preference for particular conformation in a configuration dependent manner.^76^ It was therefore of interest to examine the side chain conformations of gentamicins C, C1, C2a, and B1. To this end, NMR spectroscopic studies were conducted on compounds C2 (**3**) and C2a (**4**) in aqueous solution at pD5. Gentamicin C2 **3** has a ^3^*J*_H5′,H6′_ coupling constant of 3.3 Hz, indicating a predominantly *gauche* relationship between H’s 5′ and 6′. In the ROESY spectrum, its 6′-methyl group exhibits a strong correlation to the equatorial H4′ and to H5′ and a slightly weaker correlation to the axial H4’. Together with the absence of any correlation between H6′ and either of the axial or equatorial H4′s, these observations suggest that the side chain of C2 (**3**) predominantly populates the *gauche-gauche* (*gg*) conformation (Figure 3).^77,78^ In contrast, C2a (**4**) has a ^3^*J*_H5′,H6′_ coupling constant of 7.4 Hz, strong ROESY interactions between the 6′-methyl group and both the equatorial H4′-equatorial and H5′, and only a very weak correlation between the 6′-methyl group and the axial H4′. C2a (**4**) also displays a ROESY correlation between H6′ and the axial H4′-axial that is approximately twice as intense as that between H6′ and the equatorial H4′, overall suggesting that the side chain of C2 (**4**) is a mixture of a major *gauche,trans* (*gt*) and a minor *gg* conformation (Figure 3). We did not conduct ROESY studies on gentamicin C1, but note that it has the same relative configuration as C2 and a ^3^*J*_5’,6’_ of 3.2 Hz, leading us to assign it a predominant *gg* conformation. We previously carried out conformational analysis of both of 6′*R*- and 6′*S*-methyl neomycin and assigned them predominant *gt* and *gg* conformations, respectively:^76^ by analogy, gentamicin B1 (**7**) with its 6′*R*-configuration will predominantly populate the *gt* conformation in which the 6′-amino group is *gauche* to O5′ and trans to C4′. The difference in predominant side chain conformations between gentamicins C2 (**3**) and B1 (**7**) (*gg* and *gt*, respectively), despite the presence of a 6′*R*-methyl substituent in both compounds, is due to the additional C4′-O bond in **7** that destabilizes the *gg* conformation.

### Cell-Free Translation Assays

**Figure 4:**
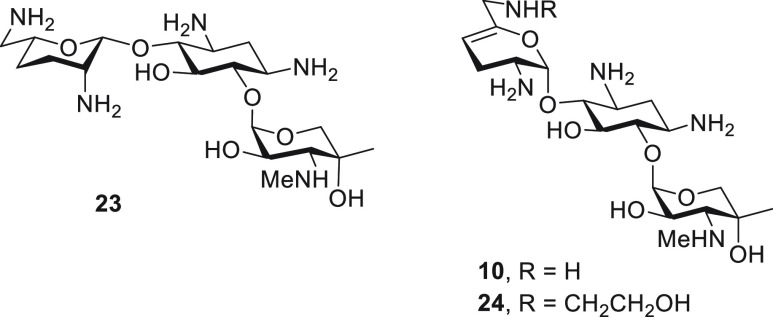
Structure of 5′-epigentamicin C1a (**23**), sisomicin (**10**), and 6′*N*-(hydroxyethyl) sisomicin (**24**).

The ability of commercial gentamicin complex, gentamicins B1, C1, C1a, C2, C2a, C2b, and X2, and 5′-epigentamicin C1a (**23**, Figure 4), a byproduct of our synthetic pathways,^45,79^ to disrupt protein synthesis was determined with cell-free translation assays of wild-type bacterial ribosomes and hybrid bacterial ribosomes with the human mitochondrial decoding A site (Mit13), the human A1555G mutant mitochondrial (A1555G), and the human cytoplasmic (Cyt14) decoding A sites (Table 1).^51−55,67,68^

**Table 1:**
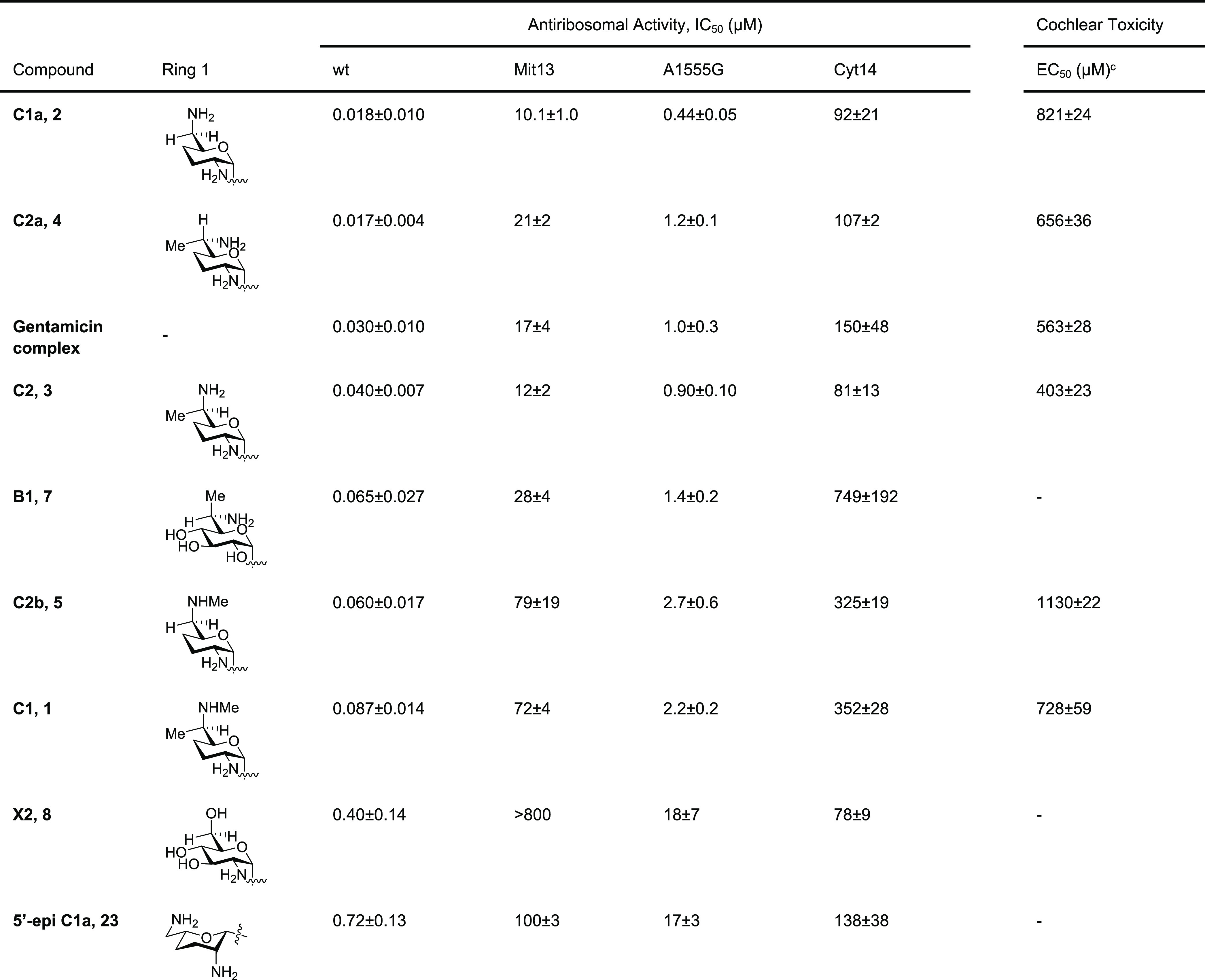
Antiribosomal Activities and Cochlear Toxicity of Commercial Gentamicins and of Individual Synthetic Components^a^^,^^b^

a Compounds arranged in order of descending wild-type antibacterial ribosomal activity.

b All values were determined in at least triplicate using a 2-fold dilution series.

c Reproduced from Ricci et al.^28^

### Inhibition of Protein Synthesis by Bacterial Ribosomes

**Figure 5:**
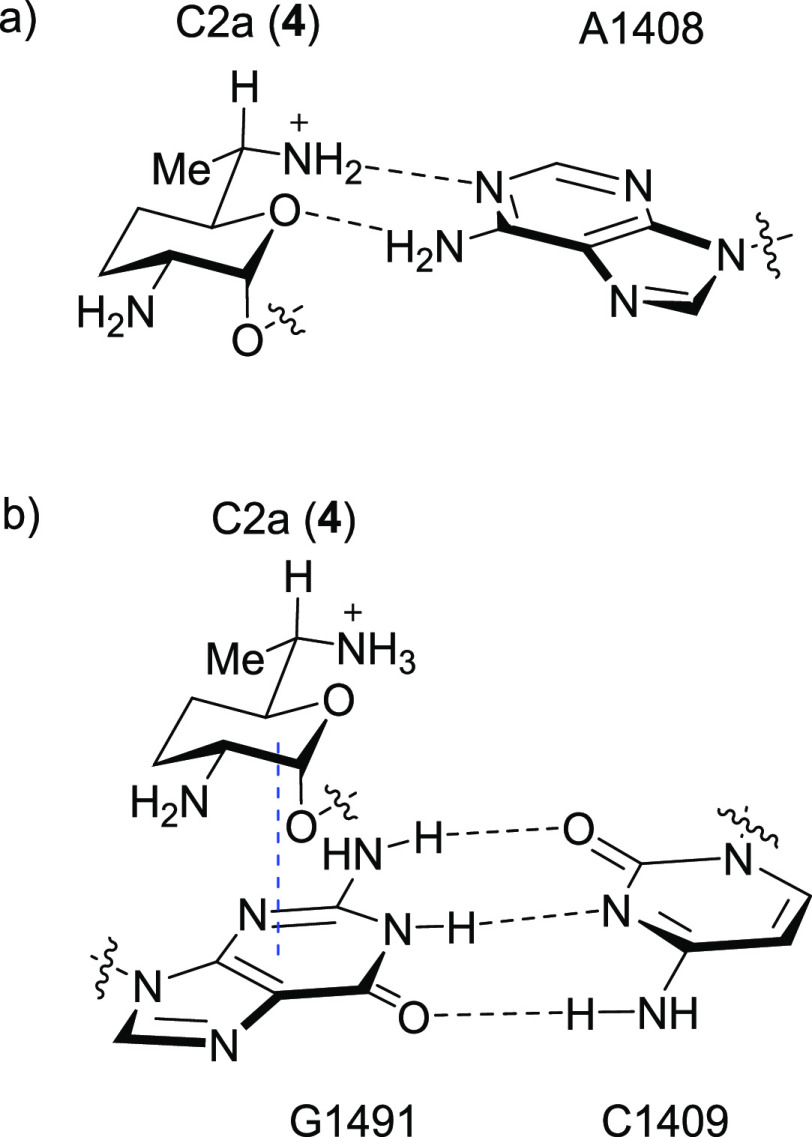
Interactions of ring 1 with the bacterial decoding A site illustrated for gentamicin C2a (**4**): (a) ideal pseudobase pair interaction with A1408 and (b) “stacking” with the G1491≡C1409 canonical base pair, where the dashed blue line indicates “stacking”.

Two compounds stand out for their poor inhibition of the bacterial ribosome: gentamicin X2 (**8**) and 5′-epigentamicin C1a (**23**). 5′-Epigentamicin C1a (**23**) is forty-fold less active than gentamicin C1a (**2**) despite the two compounds formally differing only in configuration at a single center. This is because the change in configuration at C5′ provokes the inversion of conformation of ring 1 such that the 5′-epi-isomer is a poor fit for the drug binding pocket. Comparison of the activity of gentamicin X2 (**8**) with that of the pseudo-regioisomer gentamicin B1 (**7**) reveals the far greater importance of a basic nitrogen at the 6′-position than at the 2′-position, consistent with earlier observation in the kanamycin series of 4′,6′-AGAs.^80,81^ Turning to the C1 and C2 series of compounds, only a five-fold difference in activity is seen between the least (C1, **1**) and most active (C2a, **4**) compounds, indicating the relatively minor influence of methylation at either C6′ or N6′ or both on activity. The two least active compounds are gentamicins C1 (**1**) and C2b (**5**), both of which are methylated on N6′. This result is consistent with our earlier observation that the 6′*N*-hydroxyethyl derivative (Figure 4) of sisomicin (**10**) results in a six-fold loss of inhibitory activity for the bacterial ribosome and points to the steric impact of the 6′*N*-alkyl group on an important hydrogen bond between N6′ and N1 of adenine 1408 in the pseudobase interaction of the AGA ring I with the drug binding pocket (Figure 5a). The approximately two-fold difference in activity between the epimers C2 (**3**) and C2a (**4**) arising from the presence of a methyl group at the 6′-position can be attributed to the differences in preferred side chain conformation conferred by the presence of the methyl group (Figure 2). In effect, C2a (**4**) with the predominant *gt* conformation of its side chain is preorganized to participate in the ideal pseudobase pair interaction (Figure 5a),^76,82,83^ whereas a penalty must be paid on binding of C2 (**3**) because of the need for its side chain to adopt a higher energy conformation than the ground state *gg* conformation observed in free solution. The parity in activity between C2a (**4**) and C1a (**2**), which lacks any additional *C*- or *N*-methyl group and has a freely rotating side chain, supports the notion that the two-fold difference in activity between the two 6′-methyl epimers C2 (**3**) and C2a (**4**) is due to destabilization of the pseudobase pair with A1408 by the methyl group in C2 (**3**) rather than to stabilization of the pseudobase pair with C2a (**4**) arising from preorganization of the side chain into the ideal *gt* conformation. In addition to screening the individual gentamicin congenors, we also screened a commercial gentamicin mixture^84^ and unsurprisingly found it to be moderately less active than C1a (**2**) and C2a (**4**) but somewhat more active than the remaining congeners (Table 1).

### Inhibition of Protein Synthesis by Hybrid Mitochondrial (Mit13) and Hybrid Mutant Mitochondrial (A1555G) Ribosomes and Relationship to Cochleotoxicity and Nephrotoxicity

**Figure 6:**
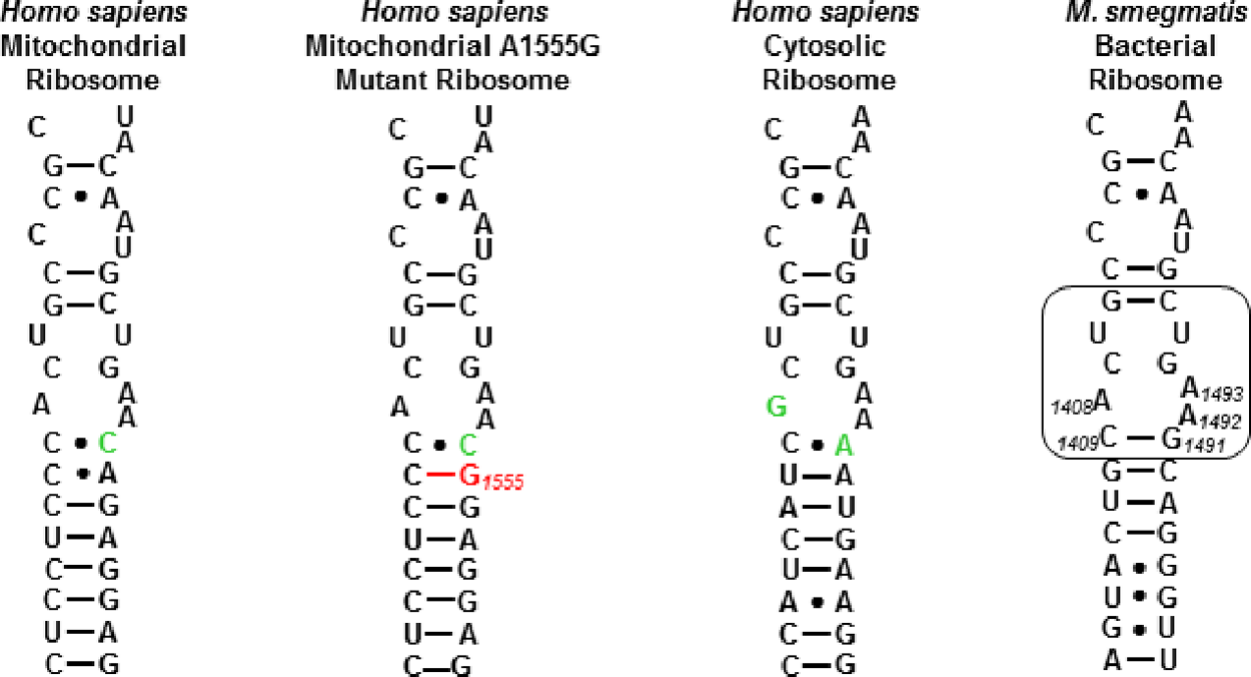
Decoding A sites of the human mitochondrial, A1555G mutant mitochondrial, and cytoplasmic ribosomes and of the bacterial ribosome. The bacterial AGA binding pocket is boxed. The bacterial numbering scheme is illustrated for the AGA binding pocket. Changes from the bacterial ribosome binding pocket are colored green. The A1555G mutant conferring hypersusceptibility to AGA ototoxicity is colored red.

The Mit13 hybrid bacterial ribosome carrying the complete decoding A site of the human mitochondrial ribosome has only two differences with the bacterial ribosome, namely, the C1490A and the G1491C substitutions at the base of the drug binding pocket (Figure 6). Both the C1490A and the G1491C substitutions result in the replacement of canonical Watson–Crick base pairs by non-canonical wobble pairs (C1409•C1491 and C1410•A1490) with a consequent overall increase in mobility of the decoding A site. In the A1555G mutant mitochondrial ribosome, the C1410•A1490 wobble pair of the wild-type mitochondrial decoding A site is replaced by a canonical pair C1410≡G1490 (in the human numbering scheme this is the A1555G modification); as a result, there is only a single wobble pair at the base of the drug binding pocket (C1409•C1491, Figure 6) such that the decoding A site of the A1555G mutant mitochondrial ribosome has intermediate flexibility between that of the bacterial and the nonmutant mitochondrial ribosomes. Consequently, it is to be expected that the SAR trends for inhibition of the bacterial ribosome by the various gentamicin isomers are grossly reproduced for inhibition of the hybrid mitochondrial ribosome, albeit at a significantly lower level, and for the hybrid A1555G mutant mitochondrial ribosome with an intermediate level of activity. This prediction is borne out by the data (Table 1) with the single exception of gentamicin C2a (**4**) that is moderately less active against the two mitochondrial ribosomes than its epimer gentamicin C2 (**3**), whereas the opposite was true for inhibition of the bacterial ribosome. We attribute this change in SAR for **3** and **4** to the G1491C substitution on going from the bacterial to the mitochondrial ribosome and its A1555G mutant form, as in the bacterial ribosome G1491 sits immediately underneath the AGA ring I and “stacks” with it (Figure 5b), such that SAR about the pyranose ring 1 might be expected to be sensitive to the G1491C and other mutants at that site. Indeed, it has been demonstrated previously that mutagenesis of the C1409≡G1491 base pair differentiates between 6′-hydroxy and 6′-amino AGAs.^85^ Clearly, the precise location of the 6′-methyl group in gentamicins C2 (**3**) and C2a (**4**) impacts the interaction of these two 6′-epimers with the base located at position 1491.

Two hypotheses have been advanced for the ototoxicity of the AGAs. The first, based on the correlation between hypersusceptibility to AGA-induced ototoxicity and mutations in the mitochondrial ribosome (A1555G), suggests that ototoxicity arises from the AGA inhibition of protein synthesis in the cochlea where AGAs can be detected up to 30 days after administration despite their very rapid clearance from the body as a whole.^51−55^ The second hypothesis rests on the manner in which the AGAs block mechanotransducer (MET) channels on entry into the cochlea.^56−58^ Ricci and coworkers, using EC_50_ values for hair cell loss in rat cochlear explants as a yardstick, measured the ototoxicities after 1 h exposures to high (200-2000 μM) concentrations of chromatographically purified gentamicins C1, C1a, C2, C2a, and C2b and hospital gentamicin complex (reproduced in Table 1). While some differences in EC_50_ values were observed, it was concluded that the structural differences between the various gentamicin congeners are too small to cause significant changes in the compounds ability to permeate and block the MET channel.^28^ In particular, Ricci and coworkers could not account for the differences in EC_50_ in the isosteric pair C2 (**3**) and C2a (**4**), suggesting instead that their differences in ototoxicity likely arise from factors other than their properties as permanent blockers of the MET channel or by a mechanism of intracellular toxicity. This need for such a mechanism of intracellular toxicity is satisfied by the inhibition of the mitochondrial and mutant mitochondrial ribosomes with the more ototoxic gentamicin C2 (**3**) inhibiting both more effectively than gentamicin C2a (**4**). The correlation between inhibition of mitochondrial ribosomes and ototoxicity extends to gentamicins C1a (**2**) and C2b (**5**), which differ only by the presence of an 6′-*N*-methyl group in the latter, with N-methylation resulting in reduced inhibition of the mitochondrial and hybrid mitochondrial ribosomes and reduced ototoxicity. It further continues with the C2 (**3**) and C1 (**1**) pair, which again differ only by the presence of the 6′-*N*-methyl group in the less ototoxic and less inhibitory C1. Despite the correlation between cochlear toxicity and inhibition of mitochondrial and hybrid mitochondrial ribosome inhibition within the pairs C1a (**2**) and C2b (**5**) and C2 (**3**) and C1 (**1**), in both of which the N-methylated homolog is the least toxic, gentamicin C1a (**2**) is an outlier when the full data set is taken into account (Table 1) as its low cochlear toxicity does not correlate with its stronger inhibition of the mitochondrial ribosomes. Presumably, this is related to the smaller size of C1a (**2**), which lacks methylation on either C6′ or N6′, and a consequent less effective blockade of the MET or more effective clearance from the hair cell via the MET.^86^

Although much work has been conducted and the pathology is understood, the molecular basis of AGA nephrotoxicity is not known with certainty,^12,87,88^ which presently excludes mechanism-based design of nephrotoxicity-free AGAs. It is widely considered, however, that oxidative stress and the formation of reactive oxygen species (ROS) are involved in AGA-induced nephrotoxicity.^12,87−89^ Moreover, it has been demonstrated that AGAs enhance production of hydrogen peroxide in renal cortical mitochondria^90^ and that the co-administration of antioxidants with AGAs minimizes nephrotoxicity.^91−94^ Consequently, the possibility exists that AGA-induced-nephrotoxicity is caused at least in part by a parallel mechanism to ototoxicity such as the inhibition of protein synthesis by mitochondrial ribosomes in the kidney. Differences in nephrotoxicity of the various gentamicin congeners have been reported by various groups,^22,24,25^ with the most recent and complete study indicating gentamicin C2a (**4**) to be less toxic than C2 (**3**),^28^ which is consistent with their relative levels of inhibition of the hybrid mitochondrial and mutant mitochondrial ribosomes.

### Inhibition of Protein Synthesis by Hybrid Cytoplasmic Ribosomes (Cyt14)

The hybrid cytoplasmic ribosomes (Cyt14) differs from the bacterial ribosome by a G1491A substitution at the base of the decoding A site, resulting in a wobble pair, but more importantly also by a A1408G modification that disrupts the key pseudo base pair interaction with ring 1 of 6′-amino AGAs leading to a reduction in activity. For the 6′-hydroxy AGAs on the other hand, G1408 in the cytoplasmic ribosome is capable of accommodating a pseudobase pair interaction with ring 1 such that the loss of activity is smaller.^95^ Thus, while all compounds tested are substantially less active against the hybrid cytoplasmic ribosome than against the bacterial ribosome, the loss of activity is the least for gentamicin X2 (**8**) that carries a 6′-hydroxy group and the greatest for gentamicin B1 (**7**) in which the loss of the critical pseudobase pair interaction is not compensated for by the presence of a 2′-amino group, consistent with observations in the kanamycin 4,6-AGAs.^80,81^ The poor activity of gentamicin B1 (**7**) against the cytoplasmic ribosome is consistent with its failure to act as a read-through agent for premature termination codons,^35^ while the moderately good activity of gentamicin X2 (**8**), especially when coupled with its poor inhibition of the bacterial ribosome, supports its potential as a read-through agent.^36^

### Antibacterial Activity and Susceptibility to Aminoglycoside Modifying Enzymes

We screened the synthetic congeners for inhibition of a collection of wild-type Gram-negative pathogens (*Escherichia coli*, *Klebsiella pneumoniae*, *Acinterobacter baumannii*, *Enterobacter cloacae*) and a strain of the Gram-positive methicillin-resistant *Staphylococcus aureus* (MRSA) (Table 2).

**Table 2:**
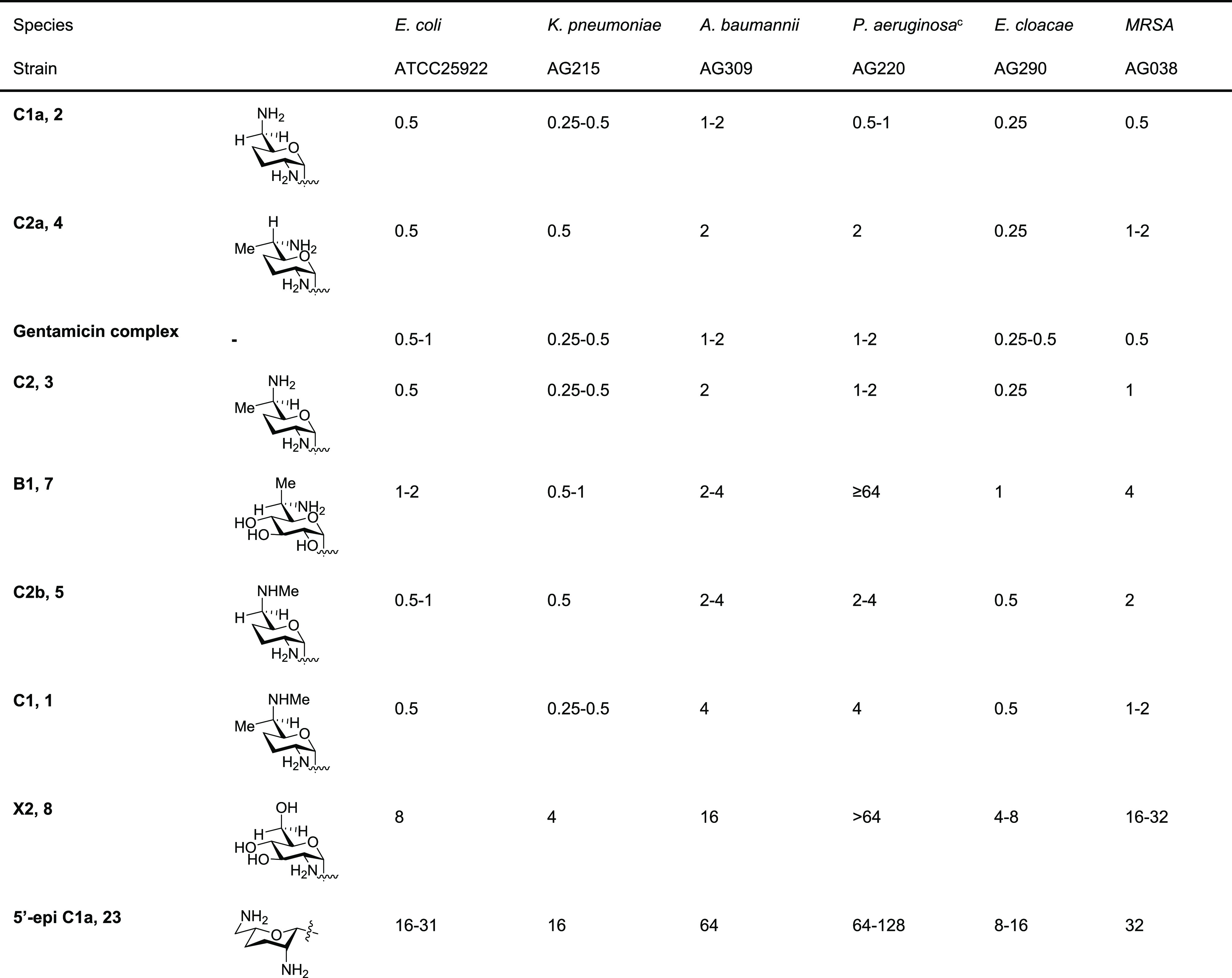
Antibacterial Activity against *E. coli* and ESKAPE Pathogens (MIC, μg/mL)^a^^,^^b^

a Arranged consistently with Table 1.

b All values were determined in at least duplicate using a 2-fold dilution series.

c *P. aeruginosa* carries a chromosomal APH(3*′*) gene.

In agreement with findings from other laboratories,^28,29^ the various gentamicin C1 and C2 congenors showed little variation in their inhibition of wild-type antibacterial activity (Table 2), consistent with the limited variation in the levels of inhibition of the bacterial ribosome (Table 1). Again consistent with their poor inhibition of the bacterial ribosome, both gentamicin X2 (**8**) and 5′-epigentamicin C1a (**23**) showed much lower levels of antibacterial activity. A minor exception to the overall congruence of the antibacterial ribosomal activity and antibacterial activity concerns the much reduced activity of gentamicin B1 (**7**), against *Pseudomonas aeruginosa*, which we attribute to the presence of an aminoglycoside phosphotransferase AME acting on the 3′-hydroxy group (APH3′)^96^ of this compound.

Finally, we screened for antibacterial activity against a set of *E. coli* strains carrying aminoglycoside acetyltransferase AMEs acting on the 3 and 6′-positions, the most common forms of resistance to gentamicin in the clinic (Table 3).^97^

**Table 3:**
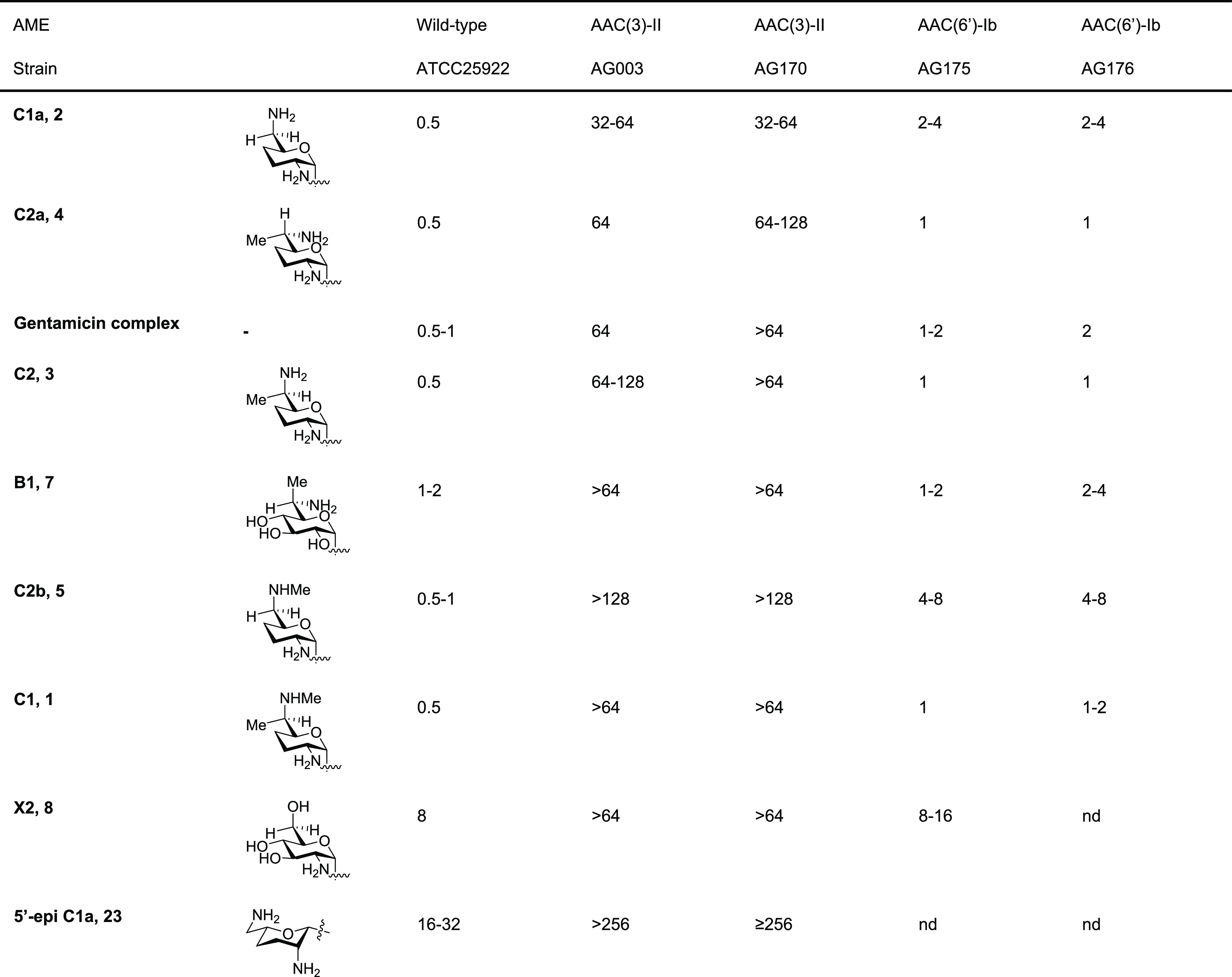
Antibacterial Activity against *E. coli* Carrying AAC(3)-II and AAC(6′)-Ib Resistance Determinants (MIC, μg/mL)^a^^,^^b^

a Arranged consistently with Table 1.

b All values were determined in at least duplicate using 2-fold dilution series.

All compounds tested lost activity in the presence of AAC(3)-II, indicating that none of the existing substitution patterns in ring I and its side chain mitigate the activity of this established mechanism of resistance to gentamicin^5^ and that it will continue to be a major cause of resistance even to preparations of single gentamicin congeners. Turning to the AAC(6′)-Ib AME that acts directly on the 6′-amino group of the gentamicins and at the site of variation among the C1 and C2 congeners, we find that the unsubstituted C1a (**2**) and its *N*-methyl variant C2b (**5**) are the most susceptible to this mode of resistance with four- to eight- and eight- to sixteen-fold losses of antibacterial activity compared to the wild type, respectively, (Table 3) and consequently that *N*-methylation does not impede the action of this AME. On the other hand, 6′-C-methylation, as in gentamicins C2 (**3**) and C2a (**4**), results in only a two to four-fold loss of antibacterial activity compared to the wild type in the presence of this AME. The AAC(6′)-Ib AME therefore tolerates methylation directly on the target amino group but poorly accommodates methylation on the carbon atom adjacent to it. Consistent with this observation, gentamicin C1 (**1**) that is methylated at both the C6′ and N6′-positions displays only a minor two- to four-fold loss of antibacterial activity. Similar observations were found by early workers in the field who noted that gentamicin C1a (**2**) was a substrate for AAC(6′), while gentamicins C1 (**1**) and C2 (**3**) were not.^71,98^

## Conclusions

Gentamicins C1 and C2 have been prepared by a straightforward stereocontrolled route from the readily available sisomicin, while gentamicin C2a was obtained from a minor isomer formed en route to C1 and C2. The inhibition of protein synthesis by these gentamicin congeners and others obtained previously by synthesis was determined using cell-free translation assays with bacterial ribosomes and hybrids carrying the decoding A site of human mitochondrial, mutant mitochondrial, and cytoplasmic ribosomes. The relatively minor differences between the various components observed in these assays and in antibacterial inhibition assays are discussed as a function of the substitution pattern around ring I and its side chain. On the basis of the little differences in antibacterial activity between the various C isoforms of gentamicin and the comparably minor differences in inhibition of the mitochondrial ribosomes together with their susceptibility to inactivation by AAC(3)-II, we consider it unlikely that there will be a significant clinical advantage in treatment with one or more of the pure isoforms as compared to the mixtures currently obtained by fermentation methods.

## Methods

### General Experimental Section

All experiments were carried out under a dry argon atmosphere unless otherwise specified. Chromatographic purifications were carried over silica gel (230–400 mesh) or Sephadex C-25 as specified. Thin-layer chromatography was performed with precoated glass-backed plates (w/UV 254). TLC plates were visualized by UV irradiation (254 nm) and by charring with sulfuric acid in ethanol (20:80, v/v) or with ceric ammonium molybdate solution [Ce(SO_4_)_2_: 4 g, (NH_4_)_6_Mo_7_O_24_: 10 g, H_2_SO_4_: 40 mL, H_2_O: 360 mL]. Optical rotations were measured at 589 nm and 22 °C on a digital polarimeter with a path length of 10 cm. NMR spectra were recorded in CDCl_3_, CD_3_OD, or D_2_O as indicated using a 600 or 900 MHz instrument, and assignments were made with the help of COSY, HMBC, and HSQC spectra. Chemical shifts (δ) are given in ppm, with multiplicities abbreviated as follows: s (singlet), m (multiplet), br (broad), d (doublet), t (triplet), q (quartet), sept (septet), br s (broad singlet), qd (quartet of doublets), ttd (triplet of triplet of doublets). High-resolution (HRMS) mass spectra were recorded in the electrospray mode with an Orbitrap analyzer. The heating of reaction mixtures was carried out on an aluminum heating block of the appropriate size. The commercial gentamicin mixture was obtained from the European Pharmacopeia Standards collection.

### Cell-Free Translation Assays

Cell-free in vitro translation inhibition assays were performed using luciferase mRNA and bacterial S30 extracts containing either wild-type bacterial or human hybrid ribosomes. In brief, firefly luciferase mRNA was transcribed in vitro using T7 RNA polymerase using a plasmid as a template in which the mammalian promoter in pGL4.14 has been replaced by the T7 bacteriophage promoter. Test articles in aqueous solution containing 0.3% Tween20 were dispensed into white 96-well plates using a digital dispenser. The test article dispension volume was balanced to a total of 1.5 μL by 0.3% Tween20 in water. The reaction volume was brought to 15 μL by addition of 13.5 μL of Translation Master Mix comprising bacterial S30 extract, 0.2 mM amino acid mix, 6 μg tRNA, 0.4 μg hFluc mRNA, 0.3 μL protease inhibitor, 12 U RNAse inhibitor, and 6 μL S30 premix without amino acids. Dispension and mixing of reagents was performed on ice prior to incubating the sealed plates at 37 °C. After 1 h of incubation, the reaction was stopped on ice and 75 μL of luciferase assay reagent was added to each well. Luminescence was recorded with a plate reader.

### Antibacterial Inhibition Assays

The minimal inhibitory concentrations (MIC) of synthesized compounds were determined by broth microdilution assays according to CLSI reference methodology M07^99^ as described previously^100^ and using strains described previously.^101^ Clinical bacterial isolates were obtained from the diagnostic laboratories of the Institute of Medical Microbiology, University of Zurich.

## Supporting Information Available

Full experimental details, characterization data for all new compounds, and copies of ^1^H, ^13^C, and ^2^D NMR spectra (PDF)
