# Supplementary material for: Synthesis of Gentamicins C1, C2, and C2a and Antiribosomal and Antibacterial Activity of Gentamicins B1, C1, C1a, C2, C2a, C2b, and X2
Source: ACS Infect Dis. 2023 Jul 23;9(8):1622–33. doi: 10.1021/acsinfecdis.3c00233 (PMC10425985; doi:10.1021/acsinfecdis.3c00233)

Supporting Information

Synthesis of Gentamicins C1, C2 and C2a and Antiribosomal and Antibacterial Activity of Gentamicins B1, C1, C1a, C2, C2a, C2b, and X2.

Santanu Jana,[a,b] Parasuraman Rajasekaran,[a,b] Klara Haldimann,[c] Andrea Vasella,[d] Erik C. Böttger,[c] Sven N. Hobbie,[c] and David Crich[a,b,e,*]

a) Department of Pharmaceutical and Biomedical Sciences, University of Georgia, 250 West Green Street, Athens, GA 30602, USA
b) Complex Carbohydrate Research Center, University of Georgia, 315 Riverbend Road, Athens, GA 30602, USA
c) Institute of Medical Microbiology, University of Zurich, Gloriastrasse 30, 8006 Zürich, Switzerland
d) Organic Chemistry Laboratory, ETH Zürich, Vladimir-Prelog-Weg 1-5/10, 8093 Zürich, Switzerland
e) Department of Chemistry, University of Georgia, 302 East Campus Road, Athens, GA 30602, USA,

Email: David.crich@uga.edu

Table of Contents

	Expt	Spectra
6′-(R_S)-*N*-(*tert*-Butylsulfinyl)imino-1,3,2′-tri(deamino)-1,3,2′-triazido-3″-*N*,4″-*O*-carbonyl-gentamicin C1a (**16**)	S3	S12-S16
6′-(R_S)-*N*-(*tert*-Butylsulfinyl)amino-1,3,2′-tri(deamino)-1,3,2′-triazido-3″-*N*,4″-*O*-carbonyl-gentamicin C2 (**17**) and 6′-(R_S)-*N*-(*tert*-Butylsulfinyl)amino-1,3,2′-tri(deamino)-1,3,2′-triazido-3″-*N*,4″-*O*-carbonyl-gentamicin C2a (**18**)	S4	S17-S26
Gentamicin C2 (**3**)	S6	S27-S34
6′-(S_R)-*N*-(*tert*-Butylsulfinyl)imino-1,3,2′-tri(deamino)-1,3,2′-triazido-3″-*N*,4″-*O*-carbonyl-gentamicin C1a (**19**)	S7	S35-S39
6′-(S_R)-*N*-(*tert*-Butylsulfinyl)amino-1,3,2′-tri(deamino)-1,3,2′-triazido-3″-*N*,4″-*O*-carbonyl-gentamicin C2 (**20**) and 6′-(S_R)-*N*-(*tert*-Butylsulfinyl)amino-1,3,2′-tri(deamino)-1,3,2′-triazido-3″-*N*,4″-*O*-carbonyl-gentamicin C2a (**21**)	S8	S40-S49
Gentamicin C2a (**4**)	S9	S50-S57
6′-*N*-Benzyl-1,3,2′-tri(deamino)-1,3,2′-triazido-3″-*N*,4″-*O*-carbonyl-gentamicin C1 (**22**)	S10	S58-S62
Gentamicin C1 (**1**)	S11	S63-S67
References	S11	

6′-(R_S)-*N*-(*tert*-Butylsulfinyl)imino-1,3,2′-tri(deamino)-1,3,2′-triazido-3″-*N*,4″-*O*-carbonyl-gentamicin C1a (16):

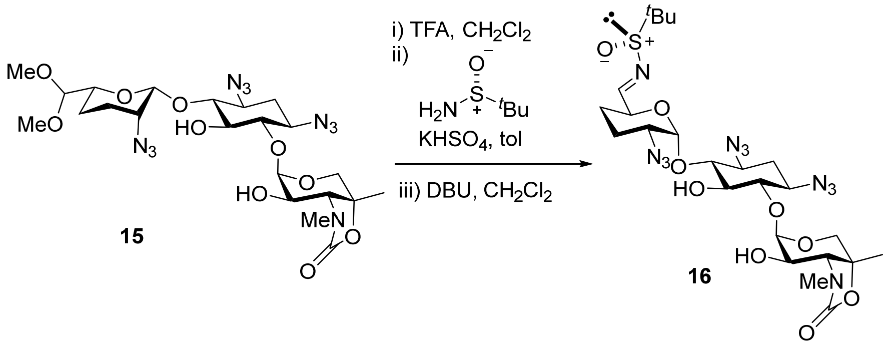

A stirred solution of compound **15**[1] (72 mg 0.12 mmol) in CH_2Cl_2 (1.2 mL) was treated with trifluoroacetic acid (0.12 mL, 10% v) and stirred at room temperature for 5 h. The reaction mixture was diluted with toluene (2 mL) and the solvents were removed under reduced pressure and co-evaporated with toluene (2 mL). The intermediate aldehyde was dissolved in toluene (1.2 mL) and treated with $KHSO_4$ (49.0 mg, 0.36 mmol) and (*R*)-*tert*-butylsulfinamide (15 mg, 0.12 mmol) and stirred at 40 °C for 3 h. Next, the reaction mixture was filtered through a syringe filter and the solvent was removed under reduced pressure. The crude product was dissolved in CH_2Cl_2 and stirred with 1,8-diazabicyclo[5.4.0]undec-7-ene (54 μL, 0.36 mmol) at 40 °C for 12 h. The solvents were removed under reduced pressure and the residue was purified by silica column chromatography (70→90% ethyl acetate in hexane) to obtain compound **16** (55 mg, 69%) as a white amorphous solid. $[\alpha]_D^{22}$ +7.8 (*c* 0.48, $CHCl_3$). **^{1}H NMR** (600 MHz, $CDCl_3$) δ 8.03 (d, *J* = 2.9 Hz, 1H, H6′), 5.65 (d, *J* = 3.4 Hz, 1H, H1′), 4.94 (d, *J* = 3.5 Hz, 1H, H1″), 4.91 (t, *J* = 2.9 Hz, 1H, H5′), 4.27 (t, *J* = 3.5 Hz, 1H, H2″), 4.143 (br s, 1H, OH), 4.14 (d, *J* = 12.2 Hz, 1H, H5″a), 3.78 (d, *J* = 12.3 Hz, 1H, H5″b), 3.70 (t, *J* = 8.8 Hz, 1H, H5), 3.65 (d, *J* = 3.5 Hz, 1H, H3″), 3.59 – 3.49 (m, 3H, H4, H1, H6), 3.42 (ddd, *J* = 12.5, 9.9, 4.4 Hz, 1H, H3), 3.31 – 3.25 (dt, *J* = 12.3, 3.9 Hz, 1H, H2′), 2.92 (s, 3H, H7″), 2.85 (br s, 1H, OH), 2.34 (dt, *J* = 13.4, 4.4 Hz, 1H, H2a), 2.22 – 2.10 (m, 2H, H3′a, H4′a), 2.00 (dq, *J* = 11.7, 3.7 Hz, 1H, H3′b), 1.66 (qd, *J* = 13.0, 3.9 Hz, 1H, H4′b), 1.51 (q, *J* = 12.6 Hz, 1H, H2b), 1.45 (s, 3H, H6′), 1.19 (s, 9H, tBu). **^{13}C NMR** (151 MHz, $CDCl_3$) δ 167.9, 157.5, 98.3, 96.7, 85.6, 79.9, 76.7, 75.4, 70.4, 67.1, 65.4, 63.2, 59.6, 59.2, 57.8, 57.1, 32.1, 30.4, 27.9, 24.0, 22.5, 22.3. **ESI-HRMS**: *m/z* $[M+H]^+$ Calcd for $C_{24}H_{38}N_{11}O_9S$ 656.2569; Found 656.2548.

6′-(R_S)-*N*-(*tert*-Butylsulfinyl)amino-1,3,2′-tri(deamino)-1,3,2′-triazido-3″-*N*,4″-*O*-carbonyl-gentamicin C2 (17) and 6′-(R_S)-*N*-(*tert*-Butylsulfinyl)amino-1,3,2′-tri(deamino)-1,3,2′-triazido-3″-*N*,4″-*O*-carbonyl-gentamicin C2a (18):

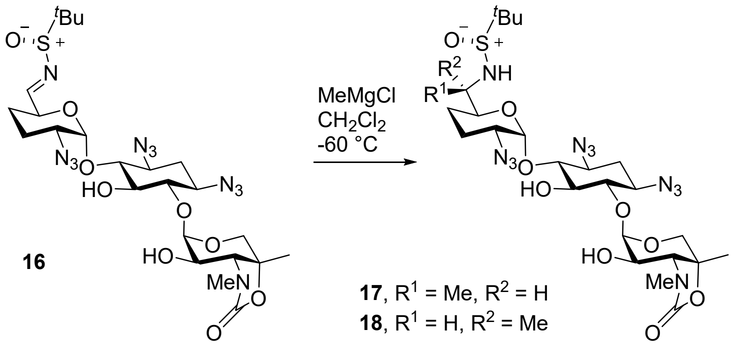

A stirred solution of compound **16** (60 mg 0.09 mmol) in CH_2Cl_2 (1.0 mL) was cooled to -60 °C and treated with MeMgCl solution (0.15 mL, 0.46 mmol, 3M in THF). After 0.5 h the reaction mixture was quenched by the addition of saturated aqueous NH_4Cl solution (2.0 mL) at -60°C. The reaction mixture was brought to room temperature, the organic solvent was separated and the aqueous portion was washed with ethyl acetate (2×2 mL). The combined organic portion was dried over Na_2SO_4, concentrated, and purified by prep HPLC over a silica column (60→90% ethyl acetate in hexane) to obtain compound **17** (40 mg, 65%) as a white amorphous solid and compound **18** (2.5 mg, 4%) as a colorless sticky gum.

(17): $[\alpha]_D^{22}$ +2.5 (*c* 0.1, $CHCl_3$). **^{1}H NMR** (600 MHz, $CDCl_3$) δ 5.59 (d, *J* = 3.4 Hz, 1H, H1′), 4.94 (d, *J* = 3.5 Hz, 1H, H1″), 4.26 (t, *J* = 3.6 Hz, 1H, H2″), 4.13 (d, *J* = 12.3 Hz, 1H, H5″a), 4.10 (dt, *J* = 12.1, 2.9 Hz, 1H, H5′), 3.77 (d, *J* = 12.3 Hz, 1H, H5″b), 3.70 (br s, 1H, OH), 3.68 (t, *J* = 8.8 Hz, 1H, H5), 3.64 (d, *J* = 3.5 Hz, 1H, H3″), 3.59 (t, *J* = 9.4 Hz, 1H, H4), 3.57 – 3.49 (m, 3H, H6, H1, H6′), 3.46 (ddd, *J* = 13.8, 10.2, 4.5 Hz, 1H, H3), 3.20 (dt, *J* = 12.6, 4.1 Hz, 1H, H2′), 2.93 (s, 3H, H7″), 2.31 (dt, *J* = 13.5, 4.4 Hz, 1H, H2a), 2.13 – 2.04 (m, 1H, H3′a), 1.96 – 1.91 (m, 1H, H3′b), 1.83 – 1.76 (m, 1H, H4′a), 1.71 – 1.56 (m, 1H, H4′b), 1.52 (q, *J* = 12.5 Hz, 1H, H2b), 1.45 (s, 3H, H6″), 1.20 (s, 9H, tBu), 1.16 (d, *J* = 6.6 Hz, 3H, H7′). **^{13}C NMR** (151 MHz, $CDCl_3$) δ 157.5, 97.7, 96.6, 85.3, 79.1, 76.7, 75.4, 71.5, 67.1, 65.4, 63.2, 59.6, 59.6, 58.4, 55.7, 53.1, 32.4, 30.4, 24.9, 24.0, 22.8, 22.2, 16.4. **ESI-HRMS**: *m/z* $[M+Na]^+$ Calcd for $C_{25}H_{41}N_{11}O_9SNa$ 694.2701; Found 694.2692.

(18): $[\alpha]_D^{22}$ +1.2 (*c* 0.3, $CHCl_3$). **^{1}H NMR** (600 MHz, $CDCl_3$) δ 5.7 (d, *J* = 3.4 Hz, 1H, H1′), 4.92 (d, *J* = 3.5 Hz, 1H, H1″), 4.27 (t, *J* = 3.5 Hz, 1H, H2″), 4.2 (br s, 1H, OH), 4.14 (d, *J* = 12.3 Hz, 1H, H5″a), 3.96 (dt, *J* = 11.6, 2.7 Hz, 1H, H5′), 3.78 (d, *J* = 12.3 Hz, 1H, H5″b), 3.70 (t, *J* = 8.8 Hz, 1H, H5), 3.65 (d, *J* = 3.5 Hz, 1H, H3″), 3.62 (t, *J* = 9.4 Hz, 1H, H4), 3.57 – 3.44 (m, 3H, H6, H1, H3), 3.33 (br s, 1H, H6′), 3.14 (dt, *J* = 12.6, 4.0 Hz, 1H, H2′), 2.93 (s, 3H, H7″), 2.87 (s, 1H, OH), 2.30 (dt, *J* = 13.4, 4.4 Hz, 1H, H2a), 2.15 – 2.06 (m, 1H, H3′a), 1.94 – 1.84 (m, 1H, H3′b), 1.76 – 1.61 (m, 2H, H4′), 1.50 (q, *J* = 12.6 Hz, 2H, H2b), 1.45 (s, 3H, H6″), 1.35 (d, *J* = 5.1 Hz, 3H, H7′), 1.21 (s, 9H, tBu). **^{13}C NMR** (151 MHz, $CDCl_3$) δ 157.5, 97.2, 96.8, 86.0, 78.4, 76.7, 75.7, 71.6, 67.2, 65.3, 63.2, 59.8, 59.6, 58.3, 56.3, 55.8, 32.4, 30.4, 26.6, 24.0, 22.9, 22.1, 19.6. **ESI-HRMS**: *m/z* $[M+Na]^+$ Calcd for $C_{25}H_{41}N_{11}O_9SNa$ 694.2702; Found 694.2691.

Gentamicin C2 (3):

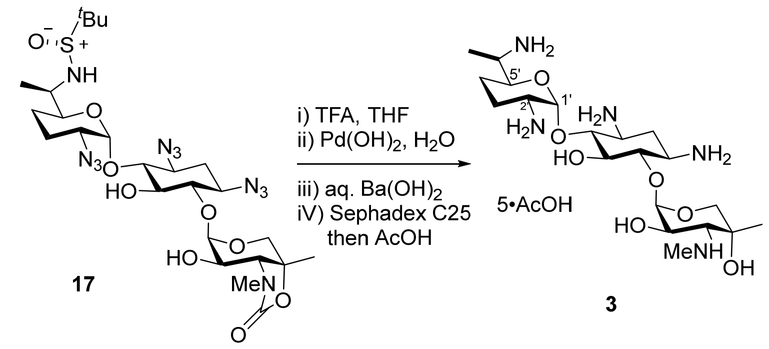

A solution of compound **17** (15.0 mg 22.3 μmol) in THF (0.5 mL) was treated with TFA (0.05 mL) and stirred for 1 h after which the solvents were removed under reduced pressure, and the crude compound was dissolved in MeOH/H_2O (2 mL, 2:1 v/v) and treated with $Pd(OH)_2$/C (5 mg, 20 wt%) and stirred at room temperature for 1 h under a hydrogen atmosphere. The reaction mixture was filtered through a syringe filter, the solvents were removed under reduced pressure, and the residue was dissolved in saturated aqueous $Ba(OH)_2$ (1 mL) and heated with stirring to 100 °C for 7 h. The reaction mixture was neutralized with dry ice and the mixture was filtered, concentrated, and purified by Sephadex C-25 column chromatography (0.06 to 06% ammonium hydroxide in water). The product-containing fractions were combined, glacial acetic acid (19 μL, 0.31 mmol) was added, and the mixture was lyophilized to give **3** as a white solid (11.0 mg, 64%) as the pentaacetate salt. $[\alpha]_D^{22}$ +108.9 (*c* 0.3, H_2O). **^{1}H NMR** (900 MHz, D_2O) δ 5.74 (d, *J* = 3.6 Hz, 1H, H1′), 5.10 (d, *J* = 3.8 Hz, 1H, H1″), 4.23 (dd, *J* = 11.0, 3.73 Hz, 1H, H2″), 4.15 (dt, *J* =

12.3, 3.0 Hz, 1H, H5′), 4.02 (d, J = 12.8 Hz, 1H, H5″), 3.79 – 3.73 (m, 2H, H4, H5), 3.69 (t, J = 9.4 Hz, 1H, H6), 3.61 (qd, J = 6.9, 3.3 Hz, 1H, H6′), 3.54 (dt, J = 12.8, 4.2 Hz, 1H, H2′), 3.52 (d, J = 12.8 Hz, 1H, H5′b), 3.50 – 3.46 (m, 1H, H1), 3.47 (d, J = 10.9 Hz, 1H, H3″), 3.32 (ddd, J = 12.7, 9.3, 4.0 Hz, 1H, H3), 2.93 (s, 3H, H7″), 2.41 (dt, J = 12.7, 4.3 Hz, 1H, H2a), 2.07 (dq, J = 12.7, 4.3 Hz, 1H, H3′a), 2.01 (qd, J = 12.7, 3.8 Hz, 1H, H3′b), 1.93 (dq, J = 12.7, 3.3 Hz, 1H, H4′a), 1.76 (q, J = 12.6 Hz, 1H, H2b), 1.60 (qd, J = 12.9, 4.0 Hz, 1H, H4′b), 1.36 (s, 3H, H6″), 1.30 (d, J = 6.9 Hz, 3H, H7′). **^{13}C NMR** (226 MHz, D_2O) δ 101.1 (C1″), 95.7 (C1′), 84.5 (C6), 79.6 (C4), 74.5(C5), 69.8 (C4″), 68.8 (C5′), 67.7 (C5″), 66.2 (C2″), 63.4 (C3″), 50.0 (C1), 49.6 (C6′), 48.9 (C2′), 48.6 (C3), 34.5 (C7″), 29.8 (C2), 22.4 (C4′), 20.9 (C6″), 20.7 (C3′), 12.7 (C7′). **ESI-HRMS**: *m/z* $[M+H]^+$ Calcd for $C_{20}H_{42}N_5O_7$ 464.3078; Found 464.3056.

6′-(S_R)-*N*-(*tert*-Butylsulfinyl)imino-1,3,2′-tri(deamino)-1,3,2′-triazido-3″-*N*,4″-*O*-carbonyl-gentamicin C1a (19):

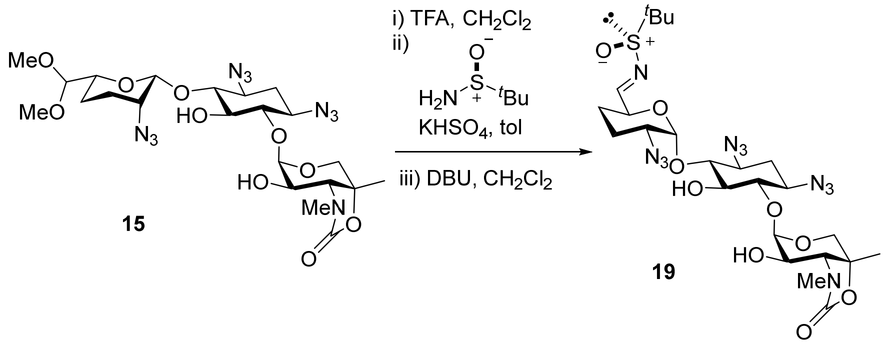

A solution of compound **15** (65 mg 0.11 mmol) in CH_2Cl_2 (1.1 mL) was treated with trifluoroacetic acid (0.1 mL, 10% v) and stirred at room temperature for 5 h. The reaction mixture was diluted with toluene (2 mL) and the solvents was removed under reduced pressure and co-evaporated with toluene (2 mL). The intermediate aldehyde was dissolved in toluene (1.2 mL) and treated with $KHSO_4$ (44 mg, 0.33 mmol) and (*S*)-*tert*-butylsulfinamide (13 mg, 0.11 mmol) and stirred at 40 °C for 3 h. Next, the reaction mixture was filtered through a syringe filter and the solvent was removed under reduced pressure. The crude compound was dissolved in CH_2Cl_2 and stirred with 1,8-diazabicyclo[5.4.0]undec-7-ene (48 µL, 0.33 mmol) at 40 °C for 12 h. The solvents were removed under reduced pressure and the residue was purified by silica column chromatography (70→90% ethyl acetate in hexane) to obtain compound **19** (43 mg, 61%) as a white amorphous solid. $[\alpha]_D^{22}$ +112.0 (*c* 0.25, $CHCl_3$). **^{1}H NMR** (900 MHz, $CDCl_3$) δ 8.03 (d, J = 3.3 Hz, 1H, H6′),

5.61 (d, J = 3.4 Hz, 1H, H1′), 4.94 (d, J = 3.4 Hz, 1H, H1″), 4.92 (t, J = 3.0 Hz, 1H, H5′), 4.26 (t, J = 3.6 Hz, 1H, H2″), 4.14 (d, J = 12.4 Hz, 1H, H5″b), 4.12 (d, J = 1.7 Hz, 1H, OH), 3.78 (d, J = 12.3 Hz, 1H, H5″b), 3.70 (td, J = 9.0, 1.7 Hz, 1H, H5), 3.65 (d, J = 3.5 Hz, 1H, H2″), 3.58 – 3.53 (m, 2H, H4, H1), 3.51 (t, J = 9.4 Hz, 1H, H6), 3.42 (ddd, J = 12.5, 10.0, 4.5 Hz, 1H, H3), 3.29 (ddd, J = 12.5, 4.6, 3.4 Hz, 1H, H2′), 2.93 (s, 3H, H7″), 2.83 (s, 1H, OH), 2.34 (dt, J = 13.3, 4.5 Hz, 1H, H2a), 2.16 (qd, J = 12.6, 4.0 Hz, 1H, H3′a), 2.12 (dq, J = 13.7, 3.3 Hz, 1H, H4′a), 2.00 (dq, J = 12.0, 3.9 Hz, 1H, H3′b), 1.69 (qd, J = 13.3, 4.0 Hz, 2H, H4′b), 1.51 (dt, J = 13.4, 12.5 Hz, 1H, H2b), 1.45 (s, 3H, H6″), 1.19 (s, 9H, tBu). **^{13}C NMR** (226 MHz, $CDCl_3$) δ 168.0, 157.5, 98.4, 96.7, 85.5, 80.0, 76.7, 75.3, 70.3, 67.1, 65.4, 63.2, 59.6, 59.2, 57.9, 57.3, 32.1, 30.4, 27.8, 24.0, 22.5, 22.4. **ESI-HRMS**: *m/z* $[M+Na]^+$ Calcd for $C_{24}H_{37}N_{11}O_9SNa$ 678.2388; Found 678.2358.

6′-(S_R)-*N*-(*tert*-Butylsulfinyl)amino-1,3,2′-tri(deamino)-1,3,2′-triazido-3″-*N*,4″-*O*-carbonyl-gentamicin C2 (20) and 6′-(S_R)-*N*-(*tert*-Butylsulfinyl)amino-1,3,2′-tri(deamino)-1,3,2′-triazido-3″-*N*,4″-*O*-carbonyl-gentamicin C2a (21)

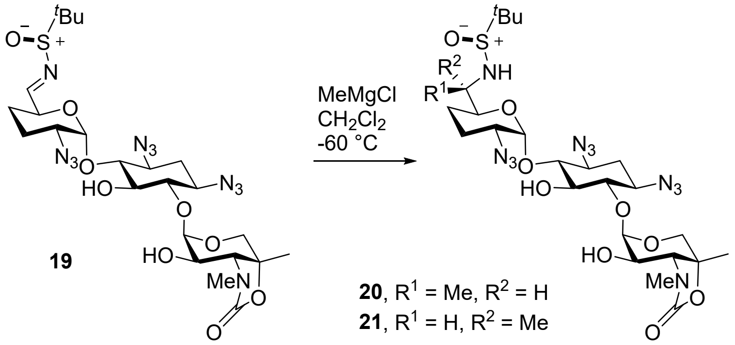

A stirred solution of compound **19** (38 mg 0.06 mmol) in CH_2Cl_2 (0.6 mL) was cooled to -60 °C and treated with MeMgCl solution (0.1 mL, 0.29 mmol, 3M in THF). After 0.5 the reaction mixture was quenched by the addition of saturated aqueous NH_4Cl solution (2.0 mL) at -60°C. The reaction mixture was brought to room temperature, the organic solvent was separated and the aqueous portion was washed with ethyl acetate (2×2 mL). The combined organic portion was dried over Na_2SO_4, concentrated, and purified by preparative HPLC over a silica column (60→90% ethyl acetate in hexane) to obtain compound **20** (25.5 mg, 65%) as a white amorphous solid and compound **21** (4.7 mg, 12%) as a colorless sticky gum.

(20): $[\alpha]_D^{22}$ +95.4 (*c* 1.1, $CHCl_3$). **^{1}H NMR** (900 MHz, $CDCl_3$) δ 5.69 (d, J = 3.4 Hz, 1H, H1′), 4.94 (d, J = 3.5 Hz, 1H, H1″), 4.26 (t, J = 3.5 Hz, 1H, H2″), 4.14 (d, J = 12.3 Hz, 1H, H5″a), 3.91

(ddd, J = 12.0, 4.6, 2.4 Hz, 1H, H5′), 3.77 (d, J = 12.3 Hz, 1H, H5″b), 3.69 (t, J = 9.0 Hz, 1H, H5), 3.64 (d, J = 3.5 Hz, 1H, H3″), 3.58 (t, J = 9.4 Hz, 1H, H4), 3.54 (ddd, J = 12.3, 9.8, 4.5 Hz, 1H, H1), 3.50 (t, J = 9.4 Hz, 1H, H6), 3.45 (ddd, J = 12.5, 9.8, 4.4 Hz, 1H, H3), 3.37 (br s, 1H, H6′), 3.21 (br s, 1H, OH), 3.17 (dt, J = 12.7, 4.1 Hz, 1H, H2′), 2.92 (s, 3H, H7″), 2.30 (dt, J = 13.4, 4.5 Hz, 1H, H2a), 2.09 (qd, J = 12.8, 4.0 Hz, 1H, H3′a), 1.93 (dq, J = 12.2, 4.0 Hz, 1H, H3′b), 1.86 (dq, J = 13.5, 3.3 Hz, 1H, H4′a), 1.52 (qd, J = 13.2, 3.9 Hz, 1H, H4′b), 1.44 (s, 3H, H6″), 1.42 (q, J = 12.9 Hz, 1H, H2b), 1.31 (d, J = 6.7 Hz, 3H, H7′), 1.21 (s, 9H, tBu). **^{13}C NMR** (226 MHz, $CDCl_3$) δ 157.5, 97.5, 96.6, 85.3, 79.0, 77.4, 76.7, 75.6, 71.8, 67.1, 65.4, 63.2, 59.9, 59.6, 58.3, 56.2, 55.9, 32.5, 30.4, 26.3, 24.0, 22.8, 22.1, 18.1. **ESI-HRMS**: *m/z* $[M+Na]^+$ Calcd for $C_{25}H_{41}N_{11}O_9SNa$ 694.2701; Found 694.2683.

(21): $[\alpha]_D^{22}$ +72.9 (c 0.2, $CHCl_3$). **^{1}H NMR** (900 MHz, $CDCl_3$) δ 5.67 (d, J = 3.4 Hz, 1H, H1′), 4.99 (d, J = 3.6 Hz, 1H, H1″), 4.22 (t, J = 3.7 Hz, 1H, H2″), 4.13 (d, J = 12.5 Hz, 1H, H5″a), 3.76 (ddd, J = 11.8, 7.1, 2.4 Hz, 1H, H5′), 3.73 (d, J = 12.5 Hz, 1H, H5″b), 3.71 (d, J = 5.4 Hz, 1H, NH), 3.67 (t, J = 8.9 Hz, 1H, H5), 3.63 (d, J = 3.7 Hz, 1H, H3″), 3.57 (t, J = 9.4 Hz, 1H, H4), 3.55 (t, J = 9.4 Hz, 1H, H6), 3.52 (ddd, J = 12.1, 9.8, 4.3 Hz, 1H, H1), 3.48 (ddd, J = 12.4, 9.8, 4.5 Hz, 1H, H3), 3.30 (td, J = 6.8, 5.4 Hz, 1H, H6′), 3.23 (ddd, J = 12.6, 4.7, 3.5 Hz, 1H, H2′), 2.92 (s, 3H, H7″), 2.31 (dt, J = 13.2, 4.4 Hz, 1H, H2a), 2.13 – 2.05 (m, 1H, H3′a), 1.95 (dq, J = 12.3, 4.0 Hz, 1H, H3′b), 1.87 (dq, J = 13.5, 3.3 Hz, 1H, H4′a), 1.58 (tdd, J = 13.3, 11.6, 4.0 Hz, 1H, H4′b), 1.42 (s, 3H, H6″), 1.41 (q, J = 12.9 Hz, 1H, H2b), 1.20 (s, 9H, tBu), 1.16 (d, J = 6.7 Hz, 3H, H7′). **^{13}C NMR** (226 MHz, $CDCl_3$) δ 157.4, 97.1, 95.7, 83.2, 79.5, 76.8, 75.2, 72.0, 66.6, 65.3, 63.1, 59.8, 59.6, 57.9, 55.5, 54.9, 32.5, 30.2, 26.8, 23.8, 22.5, 22.0, 18.2. **ESI-HRMS**: *m/z* $[M+Na]^+$ Calcd for $C_{25}H_{41}N_{11}O_9SNa$ 694.2701; Found 694.2721.

Gentamicin C2a (4)

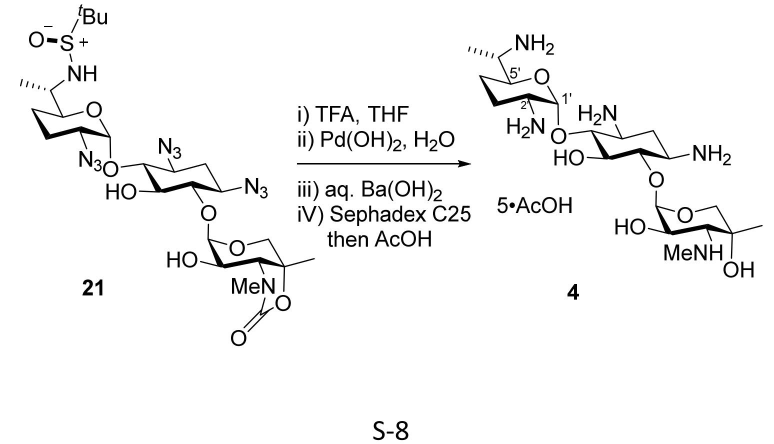

A solution of compound **21** (15.0 mg 22.3 μmol) in THF (0.5 mL) was treated with TFA (0.05 mL) and stirred for 1 h after which the solvents were removed under reduced pressure, and the crude compound was dissolved in MeOH/H_2O (2 mL, 2:1 v/v), treated with Pd$(OH)_2$/C (5 mg, 20 wt%) and stirred at room temperature for 1 h under a hydrogen atmosphere. The reaction mixture was filtered through a syringe filter, the solvents were removed under reduced pressure, and the residue was dissolved in saturated aqueous Ba$(OH)_2$ (1 mL) and heated with stirring to 100 °C for 7 h. The reaction mixture was neutralized with dry ice and the mixture was filtered, concentrated, and purified by Sephadex C-25 column chromatography (0.06 to 06% ammonium hydroxide in water). The product-containing fractions were combined, glacial acetic acid (19 μL, 0.31 mmol) was added, and the mixture was lyophilized to give **4** as a white solid (11.3 mg, 66%) as the pentaacetate salt. $[\alpha]_D^{22}$ +84.9 (*c* 0.26, H_2O). **^{1}H NMR** (900 MHz, D_2O) δ 5.79 (d, *J* = 3.6 Hz, 1H, H1′), 5.10 (d, *J* = 3.8 Hz, 1H, H1″), 4.23 (dd, *J* = 11.0, 3.8 Hz, 1H, H2″), 4.02 (d, *J* = 12.9 Hz, 1H, H5″a), 3.95 (ddd, *J* = 11.8, 7.4, 2.4 Hz, 1H, H5′), 3.80 – 3.72 (m, 2H, H4, H5), 3.70 (t, *J* = 9.3 Hz, 1H, H6), 3.54 (ddd, *J* = 9.6, 6.0, 3.6 Hz, 1H, H2′), 3.52 (d, *J* = 12.8 Hz, 1H, H5″b), 3.51 – 3.46 (m, 1H, H1), 3.48 (d, *J* = 10.9 Hz, 1H, H3″), 3.39 (p, *J* = 6.9 Hz, 1H, H6′), 3.32 – 3.27 (m, 1H, H3), 2.93 (s, 3H, H7″), 2.39 (dt, *J* = 12.8, 4.4 Hz, 1H, H2a), 2.04 – 1.98 (m, 3H, H3′, H4′a), 1.75 (q, *J* = 12.6 Hz, 1H, H2b), 1.63 – 1.51 (m, 1H, H4′b), 1.36 (s, 3H, H6″), 1.33 (d, *J* = 6.8 Hz, 3H, H7′). **^{13}C NMR** (226 MHz, D_2O) δ , 101.1 (C1″), 95.0 (C1′), 84.4 (C6), 79.3 (C4), 74.7 (C5), 69.8 (C4″, C5′), 67.7 (C5″), 66.2 (C2″), 63.4 (C3″), 50.9 (C6′), 50.0 (C1′), 48.9 (C2′), 48.6 (C3), 34.5 (C7″), 29.9 (C2), 25.4 (C4′), 20.9 (C6″), 20.5 (C3′), 14.4 (C7′). **ESI-HRMS**: *m/z* $[M+H]^+$ Calcd for $C_{20}H_{42}N_5O_7$ 464.3078; Found 464.3057.

6′-*N*-Benzyl-1,3,2′-tri(deamino)-1,3,2′-triazido-3″-*N*,4″-*O*-carbonyl-gentamicin C1 (22):

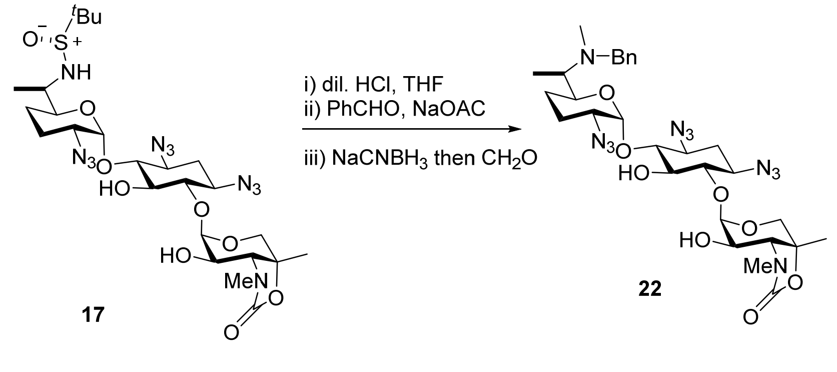

A stirred solution of compound **17** (20.0 mg 29.8 μmol) in THF (0.5 mL) was treated with 1M aqueous HCl (0.2 mL), after 1 h, 2 mL toluene was added to the reaction mixture and the solvents were removed under reduced pressure and co-evaporated with toluene (2×2 mL). The crude compound was dissolved in 1,2-dichloromethane (0.5 mL) and treated with benzaldehyde (4.0 μL, 35.7 μmol) and NaOAc (7.0 mg, 89.0 μmol) and stirred at 40 °C for 5 h. $NaCNBH_3$ (6.0 mg, 89.0 μmol) was then added to the reaction mixture and stirring continued for 5 h. Formaldehyde (25.0 μL, 37 wt.% in H_2O) was then added and the reaction mixture was stirred at room temperature for 18 h. The reaction mixture was diluted with CH_2Cl_2 (5 mL) and 1M aqueous KOH (2 mL). The organic portion was separated, and the aqueous portion was washed with CH_2Cl_2 (2×3 mL), the combined organic portion was dried over Na_2SO_4, concentrated, and purified by silica column chromatography (3% methanol in dichloromethane) to obtain compound **22** (10.2 mg, 51%) as a white amorphous solid. $[\alpha]_D^{22}$ +106.7 (*c* 0.3, MeOH). **^{1}H NMR** (600 MHz, CD_3OD) δ 7.42 – 7.26 (m, 4H, ArH), 7.25 – 7.16 (m, 1H, ArH), 5.84 (d, *J* = 3.4 Hz, 1H, H1′), 5.06 (d, *J* = 3.6 Hz, 1H, H1″), 4.24 (d, *J* = 12.7 Hz, 1H, H5″a), 4.20 – 4.16 (m, 1H, H2″), 4.13 (ddd, *J* = 11.0, 7.4, 2.4 Hz, 1H, H5′), 3.74 – 3.64 (m, 5H, H3″, H5″b, H1, H5, BnCH), 3.60 (dt, *J* = 6.1, 2.9 Hz, 2H, H3, H4), 3.55 (d, *J* = 13.4 Hz, 1H, BnCH), 3.45 (t, *J* = 9.5 Hz, 1H, H6), 3.09 (dt, *J* = 12.8, 4.3 Hz, 1H, H2′), 2.93 (s, 3H, H7″), 2.61 (p, *J* = 6.7 Hz, 1H, H6′), 2.30 (dt, *J* = 13.5, 3.9 Hz, 1H, H2a), 2.20 (br s, 4H, H8′, H4′a), 2.14 (qd, *J* = 12.6, 4.0 Hz, 1H, H3′a), 1.89 (dp, *J* = 12.2, 3.5 Hz, 1H, H3′b), 1.45 – 1.37 (m, 5H, H2b, H6″, H4′b), 1.12 (d, *J* = 6.6 Hz, 3H, H7′). **^{3}C NMR** (151 MHz, CD_3OD) δ 158.5, 139.8, 128.4, 127.8, 126.5, 97.0, 96.1, 82.3, 78.1, 77.7, 75.4, 69.6, 65.6, 65.5, 63.5, 61.0, 60.4, 60.1, 58.0, 57.9, 36.3, 32.2, 28.9, 28.5, 22.1, 21.8, 7.2. **ESI-HRMS**: *m/z* $[M+H]^+$ Calcd for $C_{29}H_{42}N_{11}O_8$ 672.3212; Found 672.3214.

Gentamicin C1 (1):

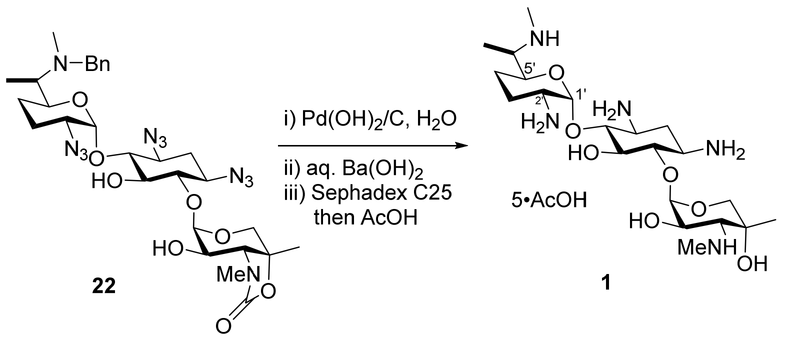

A stirred solution of compound **22** (15.0 mg 22.3 μmol) in MeOH/H_2O/AcOH (2 mL, 2:1:0.1 v/v/v) was treated with $Pd(OH)_2$/C (5 mg, 20 wt%) and stirred at room temperature for 16 h under a hydrogen atmosphere. The reaction mixture was filtered through a syringe filter, the solvents were removed under reduced pressure. The residue was dissolved in saturated aqueous $Ba(OH)_2$ (1 mL) and heated with stirring to 100 °C for 7 h. The reaction mixture was neutralized with dry ice and the mixture was filtered, concentrated, and purified by Sephadex C-25 column chromatography (0.06 to 06% ammonium hydroxide in water). The product-containing fractions were combined, glacial acetic acid (19 μL, 0.31 mmol) was added, and the mixture was lyophilized to give **1** as a white solid (9.2 mg, 53%) as the pentaacetate salt. $[\boldsymbol{\alpha}]_{\mathbf{D}}^{\mathbf{22}}$ +81.7 (*c* 0.82, H_2O). **^{1}H NMR** (900 MHz, D_2O) δ 5.73 (d, *J* = 3.6 Hz, 1H, H1′), 5.10 (d, *J* = 3.7 Hz, 1H, H1″), 4.22 (dd, *J* = 10.8, 3.8 Hz, 1H, H2″), 4.21 (dt, *J* = 12.3, 2.9 Hz, 1H, H5′), 4.02 (d, *J* = 12.8 Hz, 1H, H5″a), 3.75 (t, *J* = 9.1 Hz, 1H, H5), 3.70 (t, *J* = 9.6 Hz, 1H, H4), 3.68 (t, *J* = 9.7 Hz, 1H, H6), 3.54 (dt, *J* = 12.8, 4.3 Hz, 1H, H2′), 3.51 (d, *J* = 12.8 Hz, 1H, H5″b), 3.47 (d, *J* = 10.9 Hz, 1H, H3″), 3.46 – 3.43 (m, 1H, H1), 3.44 (qd, *J* = 6.9, 3.2 Hz, 1H, H6′), 3.32 – 3.21 (m, 1H, H3), 2.93 (s, 3H, H7′), 2.74 (s, 3H, H8′), 2.37 (dt, *J* = 13.3, 4.3 Hz, 1H, H2a), 2.09 – 1.95 (m, 2H, H3′), 1.97 – 1.91 (m, 1H, H4′a), 1.72 (q, *J* = 12.6 Hz, 1H, H2b), 1.59 (qd, *J* = 12.9, 3.9 Hz, 1H, H4′b), 1.35 (s, 3H, H6″), 1.31 (d, *J* = 6.9 Hz, 3H, H7′). **^{13}C NMR** (226 MHz, D_2O) δ 101.0 (C1″), 95.7 (C1′), 84.6 (C6), 80.1 (C4), 74.5 (C5), 69.9 (C4″), 68.9 (C5′), 67.7 (C5″), 66.2 (C2″), 63.5 (C2″), 57.7 (C6′), 50.0 (C1), 48.9 (C2′), 48.6 (C3), 34.5 (C7″), 31.7 (C8′), 30.3 (C2), 22.6 (C4′), 20.9 (C6″), 20.8 (C3′), 10.1 (C7′). **ESI-HRMS**: *m/z* $[M+H]^+$ Calcd for $C_{21}H_{44}N_5O_7$ 478.3235; Found 478.3212.

References:

1. Jana, S.; Crich, D.; Synthesis of Gentamicin Minor Components: Gentamicin C1a and Gentamicin C2b. *Org. Lett.* **2022,** *24*, 8564-8567.

6′-(R_S)-*N*-(*tert*-Butylsulfinyl)imino-1,3,2′-tri(deamino)-1,3,2′-triazido-3′′-*N*,4′′-*O*-carbonyl-gentamicin C1a (^{1}H 600 MHz, CDCl$_3$) (16):

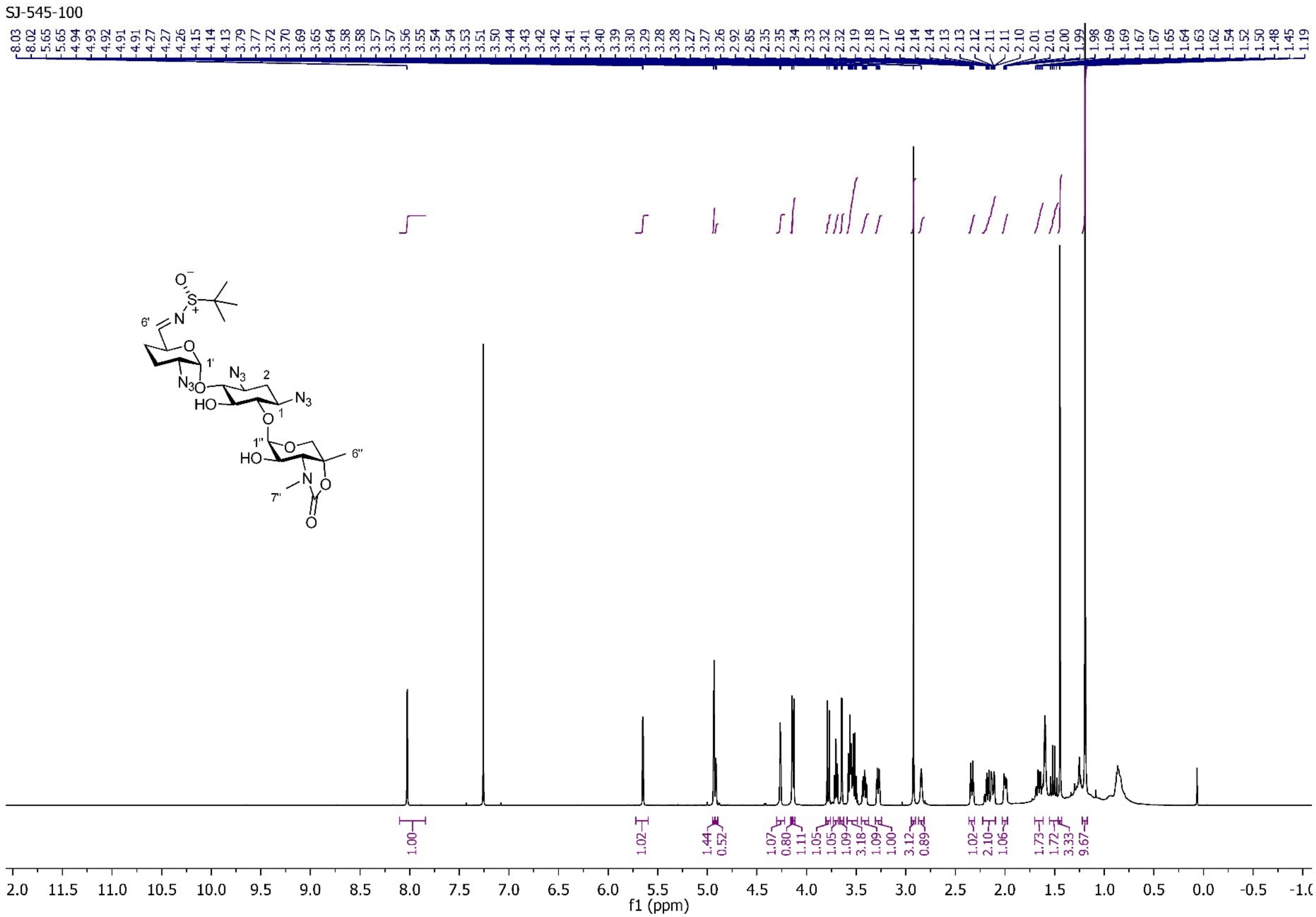

6′-(R_s)-*N*-(*tert*-Butylsulfinyl)imino-1,3,2′-tri(deamino)-1,3,2′-triazido-3′′-*N*,4′′-*O*-carbonyl-gentamicin C1a (^{13}C 151 MHz, $CDCl_3$) (16):

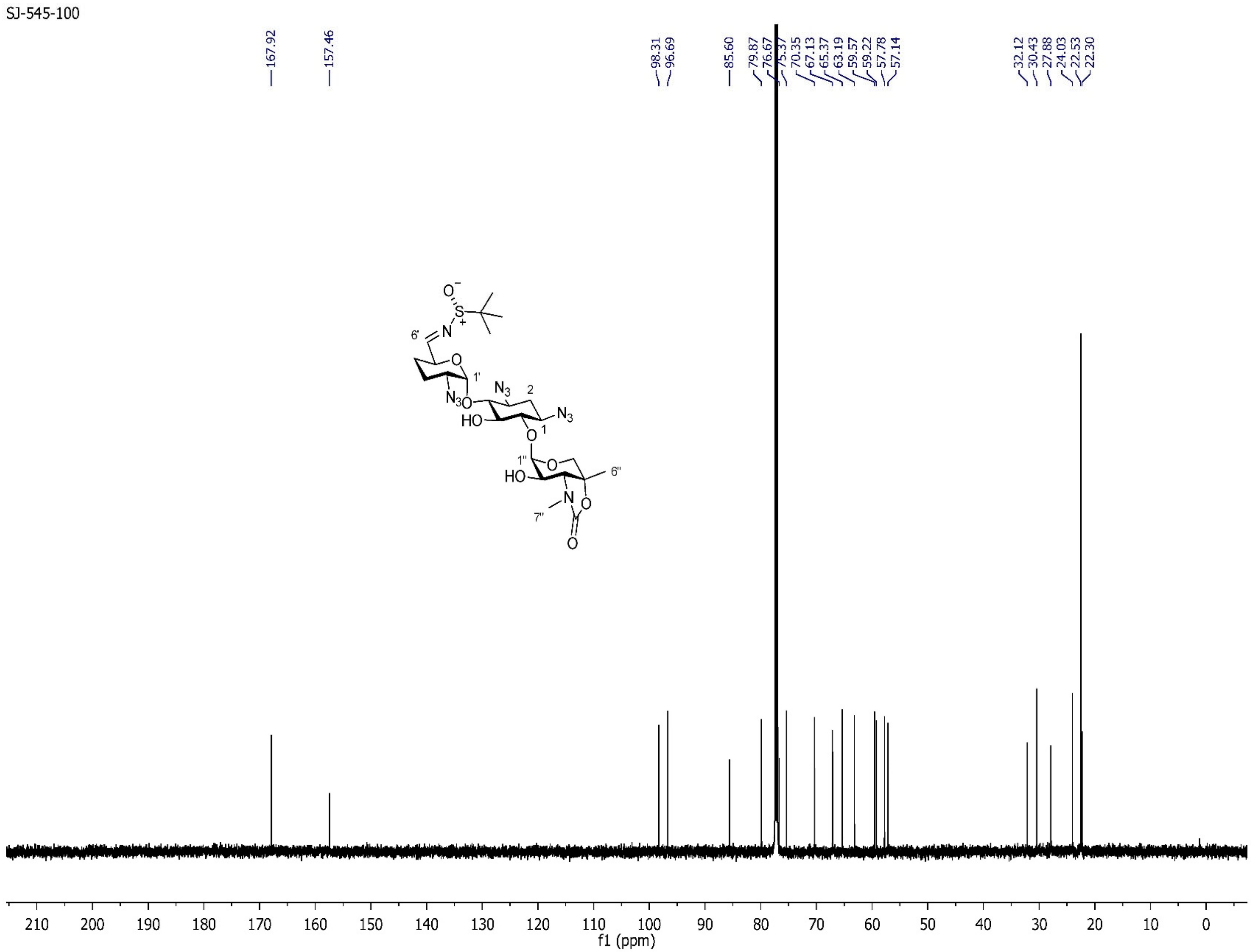

6′-(*R*$_S$)-*N*-(*tert*-Butylsulfinyl)imino-1,3,2′-tri(deamino)-1,3,2′-triazido-3′′-*N*,4′′-*O*-carbonyl-gentamicin C1a (^{1}H-^{1}H-COSY 600 MHz, $CDCl_3$) (16):

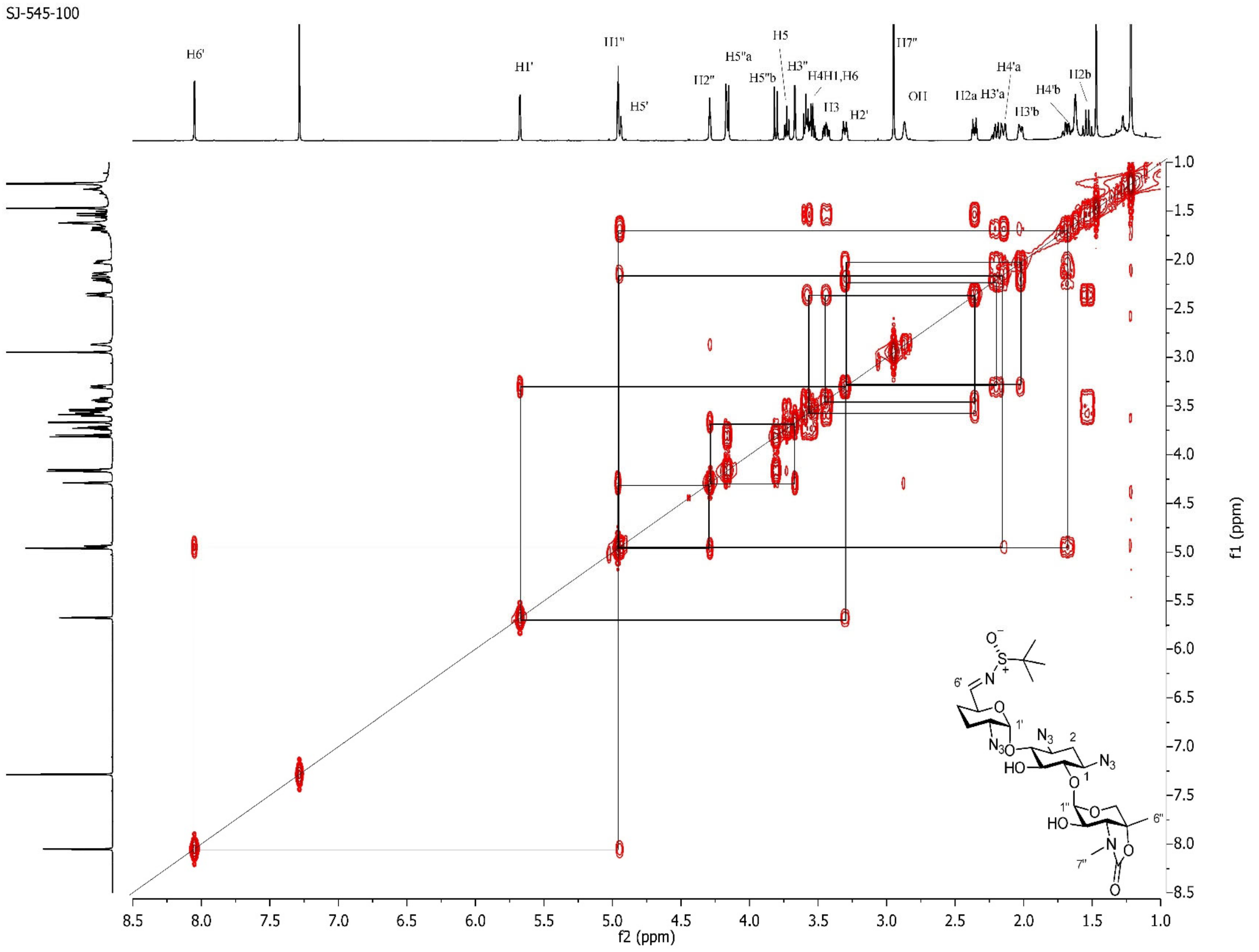

6′-(R_s)-*N*-(*tert*-Butylsulfinyl)imino-1,3,2′-tri(deamino)-1,3,2′-triazido-3′′-*N*,4′′-*O*-carbonyl-gentamicin C1a (^{1}H-^{13}C-HSQC 600 MHz, $CDCl_3$) (16):

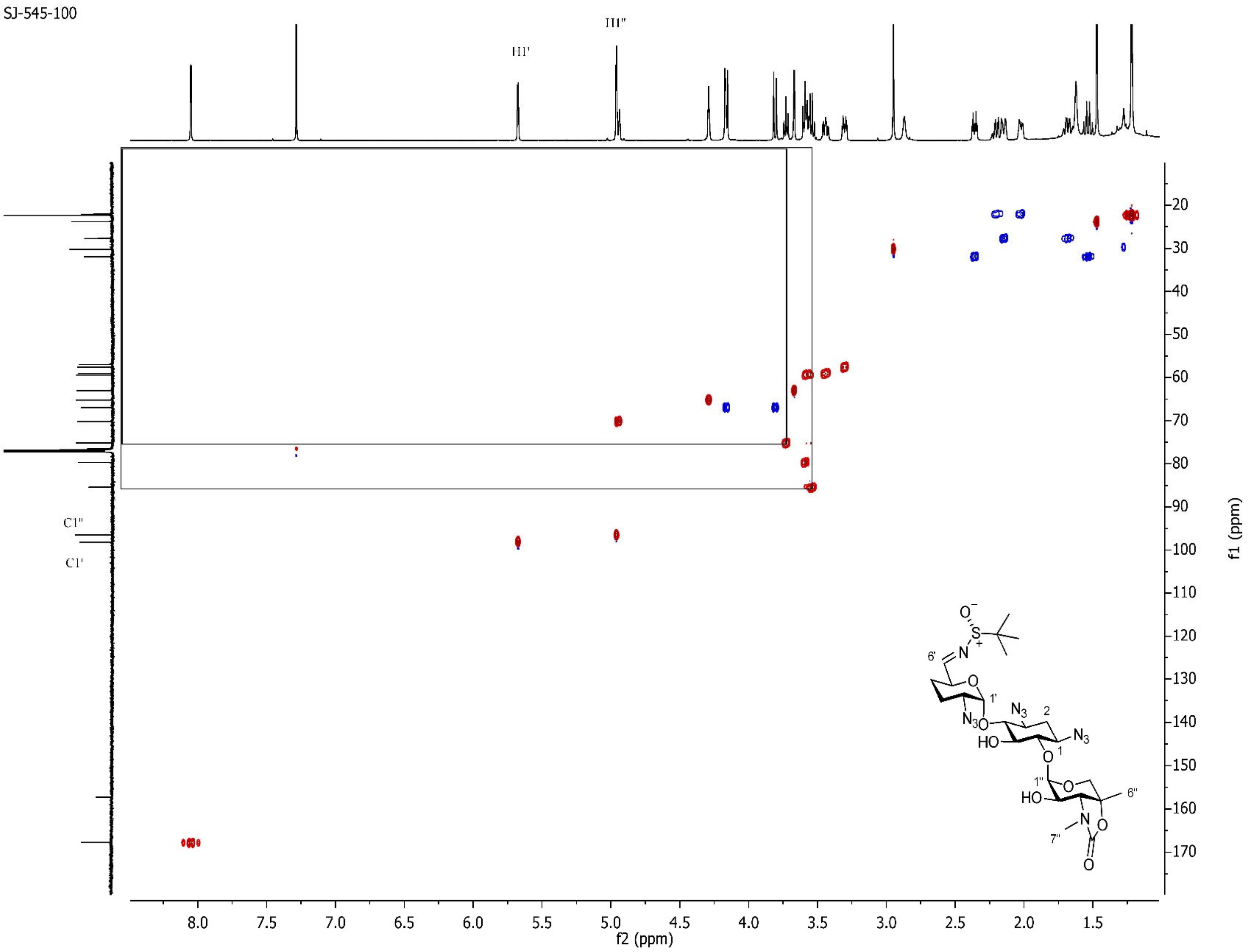

6′-(R_S)-*N*-(*tert*-Butylsulfinyl)imino-1,3,2′-tri(deamino)-1,3,2′-triazido-3′′-*N*,4′′-*O*-carbonyl-gentamicin C1a (^{1}H-^{13}C-HMBC 600 MHz, $CDCl_3$) (16):

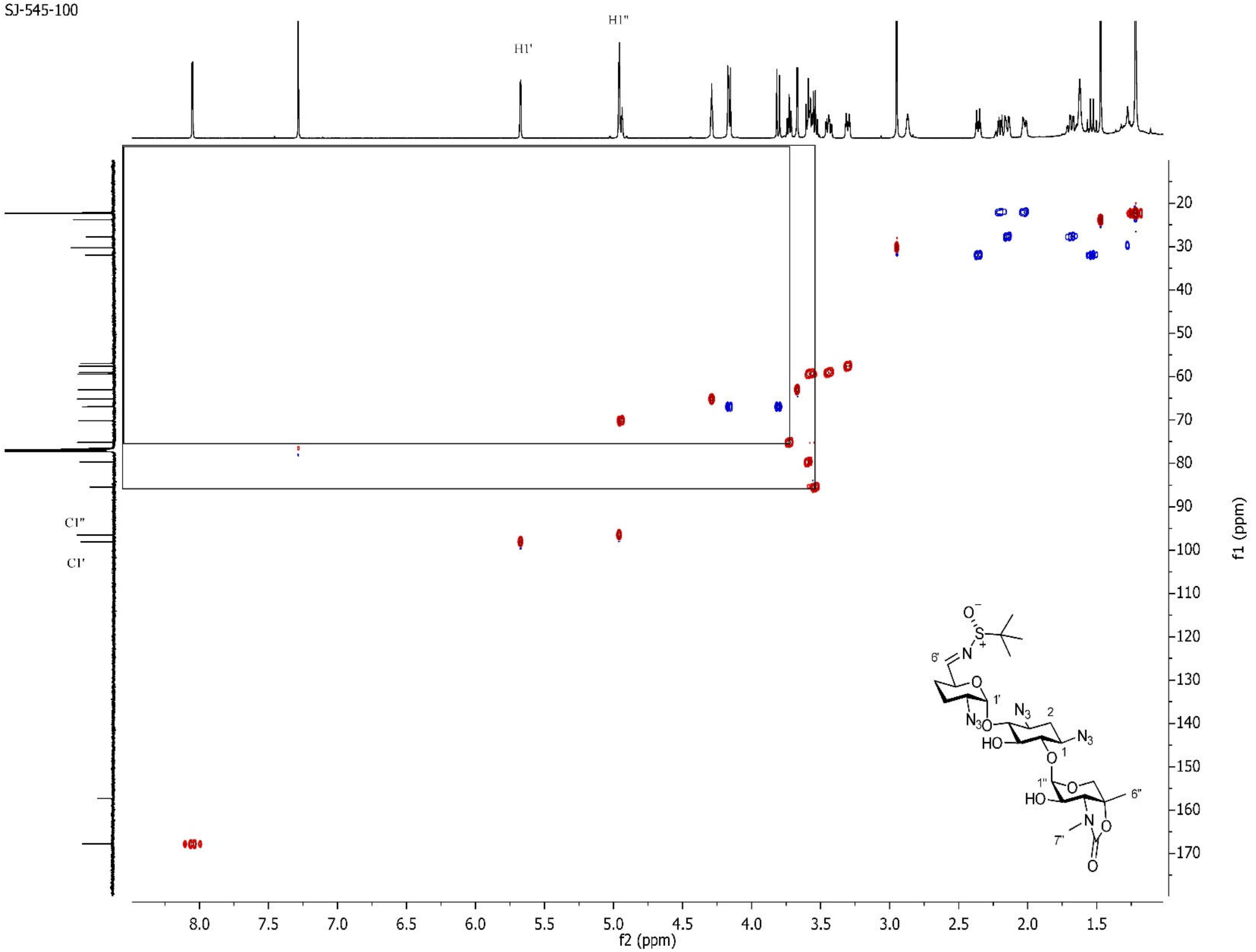

6′-(*R*s)-*N*-(*tert*-Butylsulfinyl)amino-1,3,2′-tri(deamino)-1,3,2′-triazido-3′′-*N*,4′′-*O*-carbonyl-gentamicin C2 (^{1}H 600 MHz, CDCl$_3$) (17):

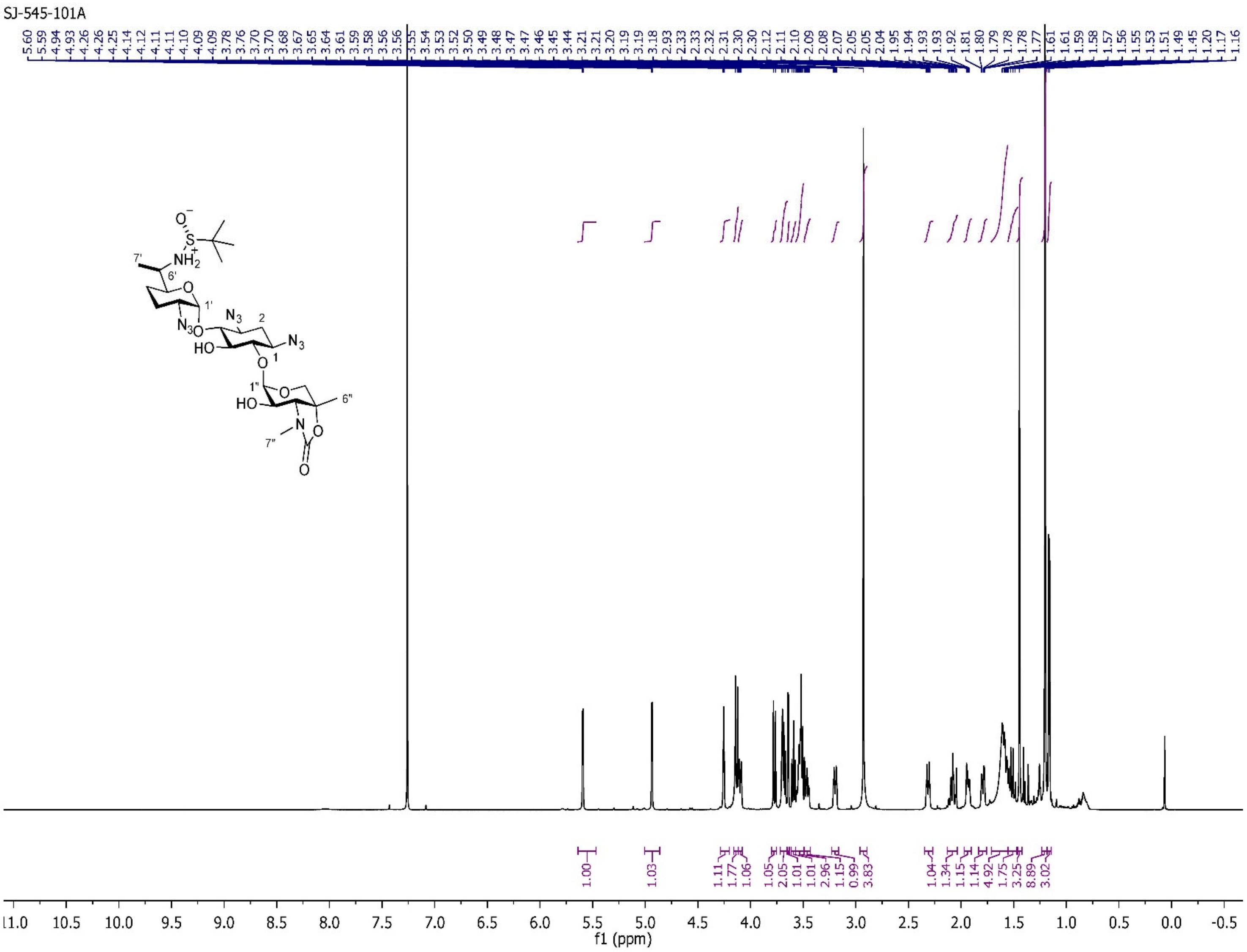

6′-(R_S)-*N*-(*tert*-Butylsulfinyl)amino-1,3,2′-tri(deamino)-1,3,2′-triazido-3″-*N*,4″-*O*-carbonyl-gentamicin C2 (^{13}C 151 MHz, CDCl$_3$) (17):

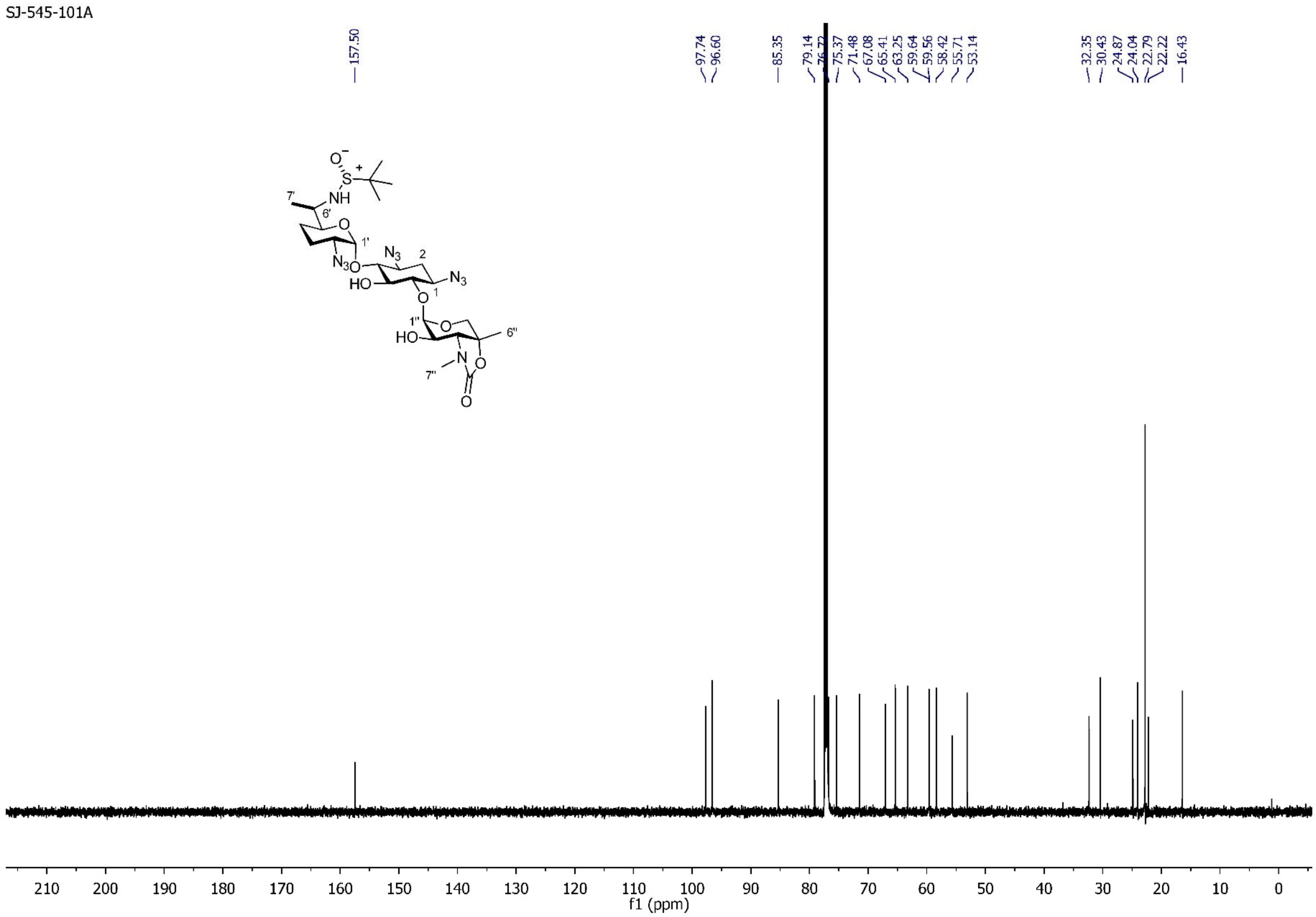

6′-(R_S)-*N*-(*tert*-Butylsulfinyl)amino-1,3,2′-tri(deamino)-1,3,2′-triazido-3″-*N*,4″-*O*-carbonyl-gentamicin C2 (^1H-^1H-COSY 600 MHz, $CDCl_3$) (17):

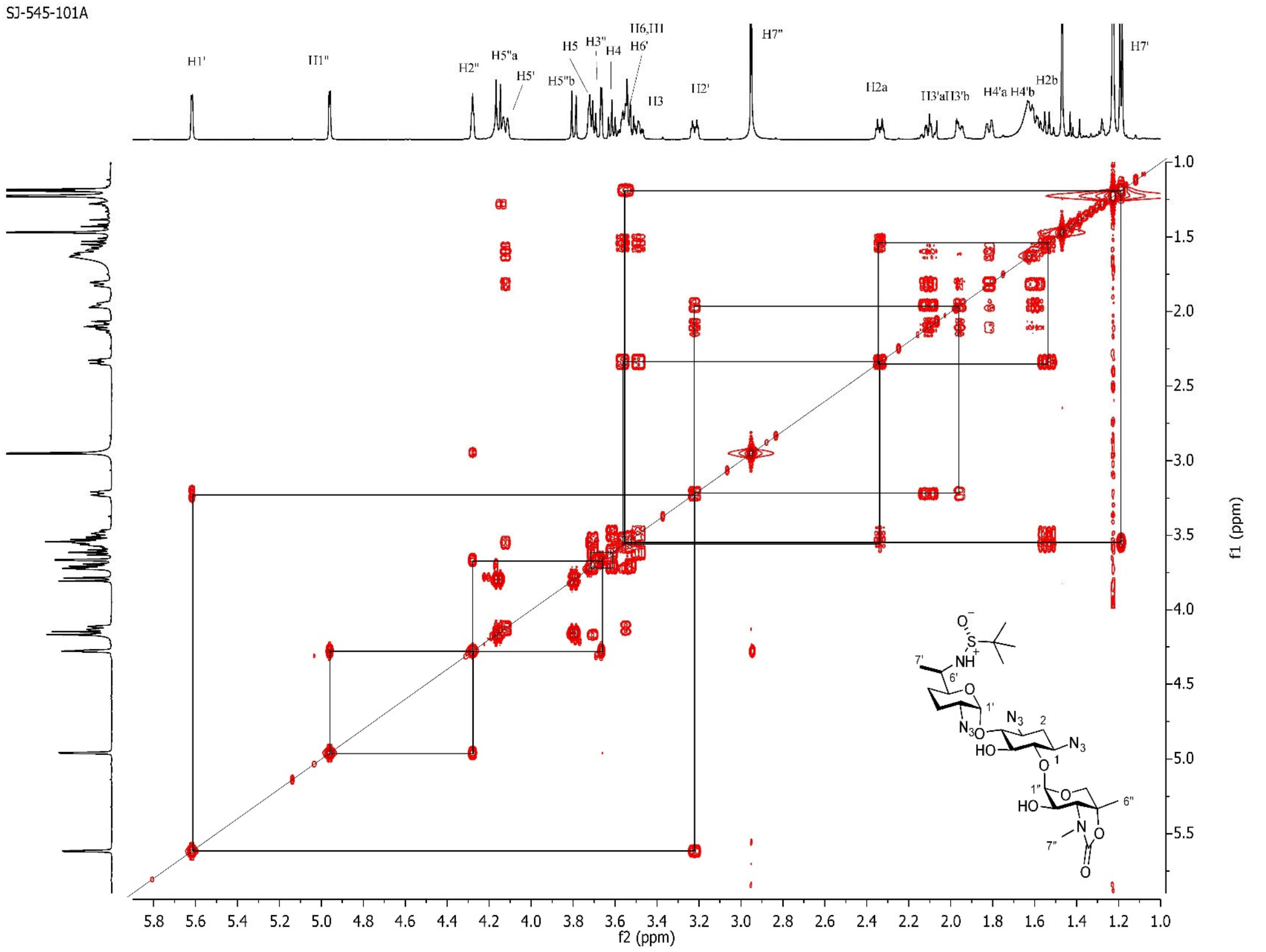

6′-(R_S)-*N*-(*tert*-Butylsulfinyl)amino-1,3,2′-tri(deamino)-1,3,2′-triazido-3′′-*N*,4′′-*O*-carbonyl-gentamicin C2 (^{1}H-^{13}C-HSQC 600 MHz, $CDCl_3$) (17):

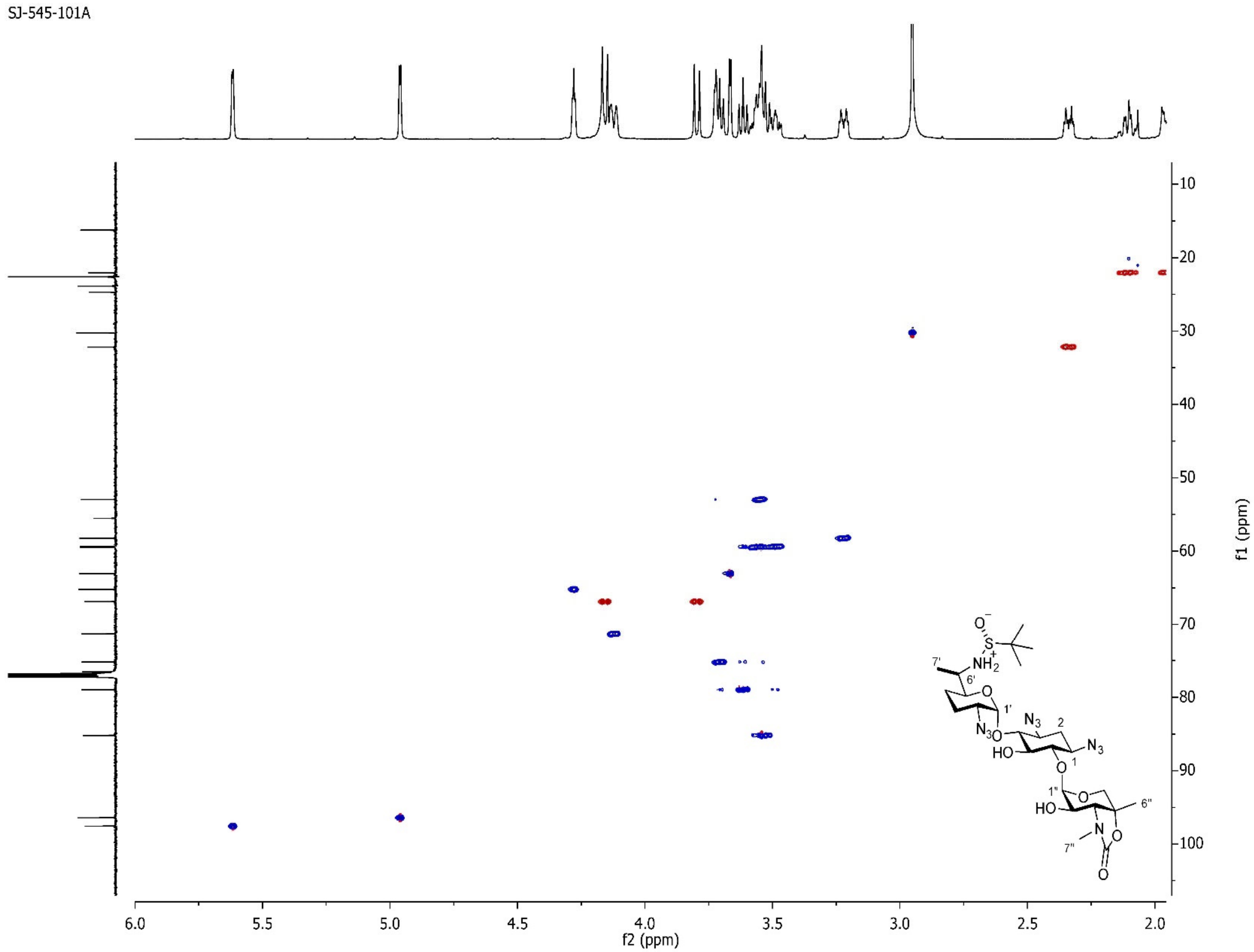

6′-(R_S)-*N*-(*tert*-Butylsulfinyl)amino-1,3,2′-tri(deamino)-1,3,2′-triazido-3′′-*N*,4′′-*O*-carbonyl-gentamicin C2 (^{1}H-^{13}C-HMBC 600 MHz, $CDCl_3$) (17):

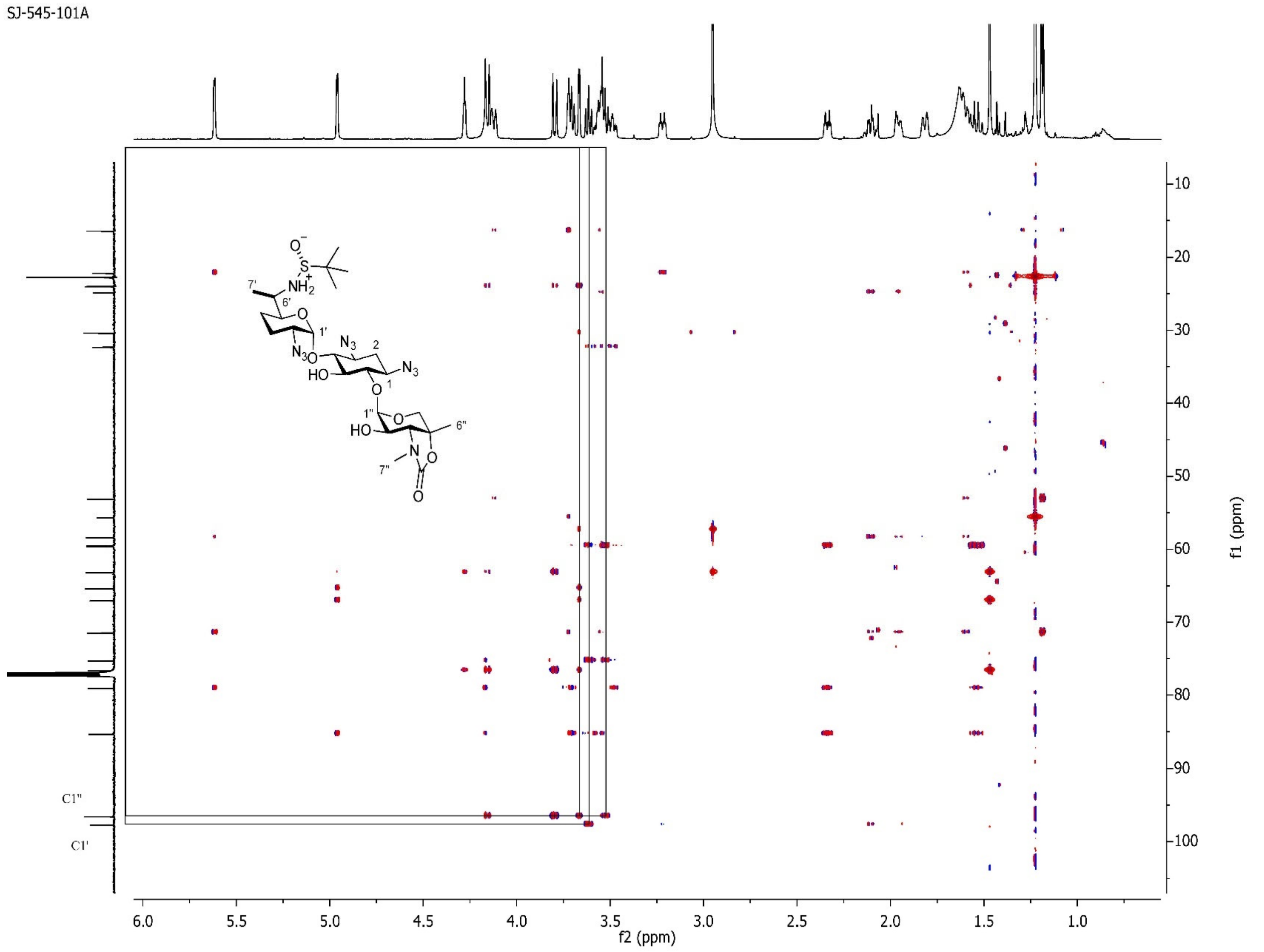

6′-(*R*s)-*N*-(*tert*-Butylsulfinyl)amino-1,3,2′-tri(deamino)-1,3,2′-triazido-3′′-*N*,4′′-*O*-carbonyl-gentamicin C2a (^{1}H 600 MHz, CDCl$_3$) (18):

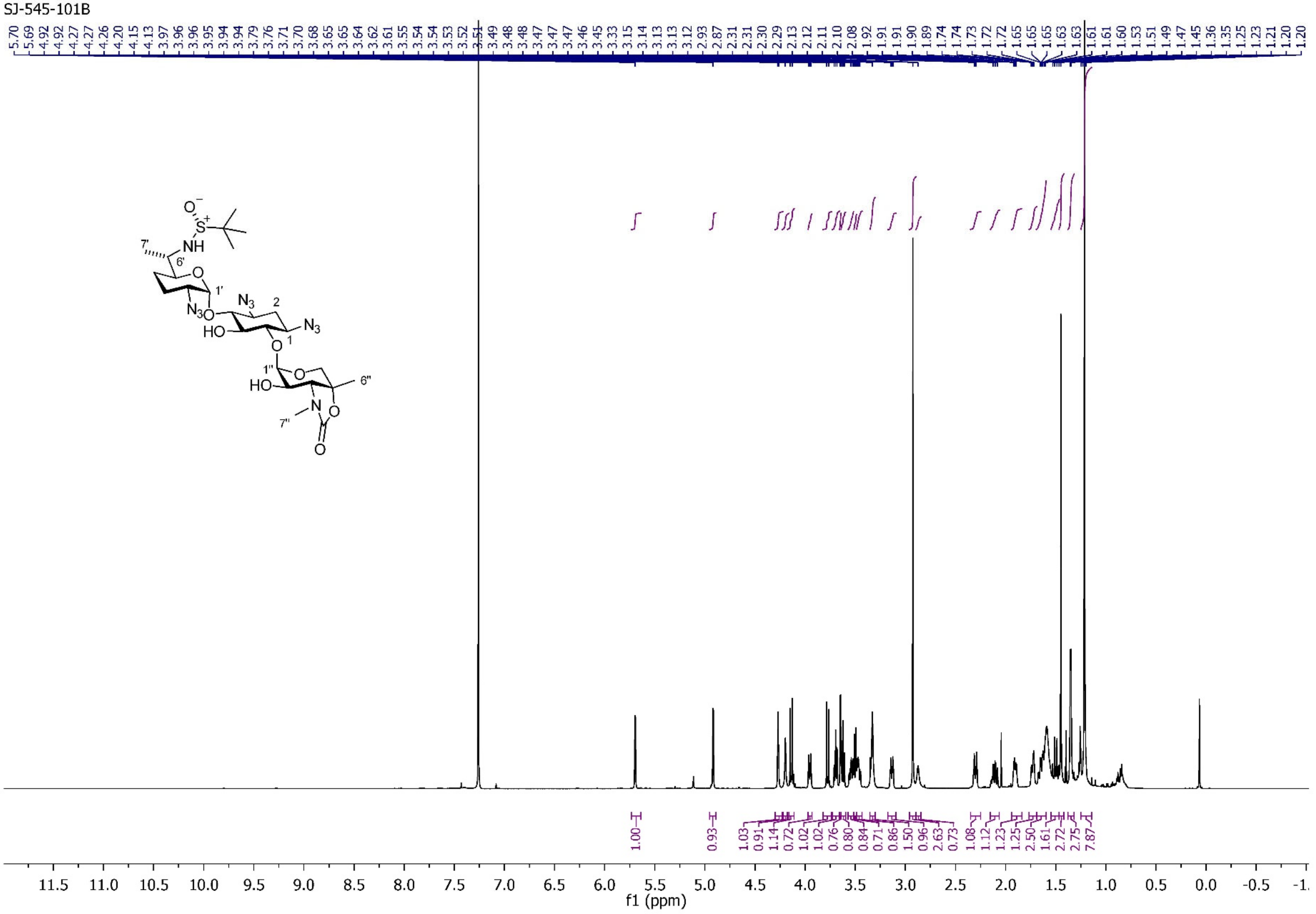

6′-(R_S)-*N*-(*tert*-Butylsulfinyl)amino-1,3,2′-tri(deamino)-1,3,2′-triazido-3″-*N*,4″-*O*-carbonyl-gentamicin C2a (^{13}C 151 MHz, CDCl$_3$) (18):

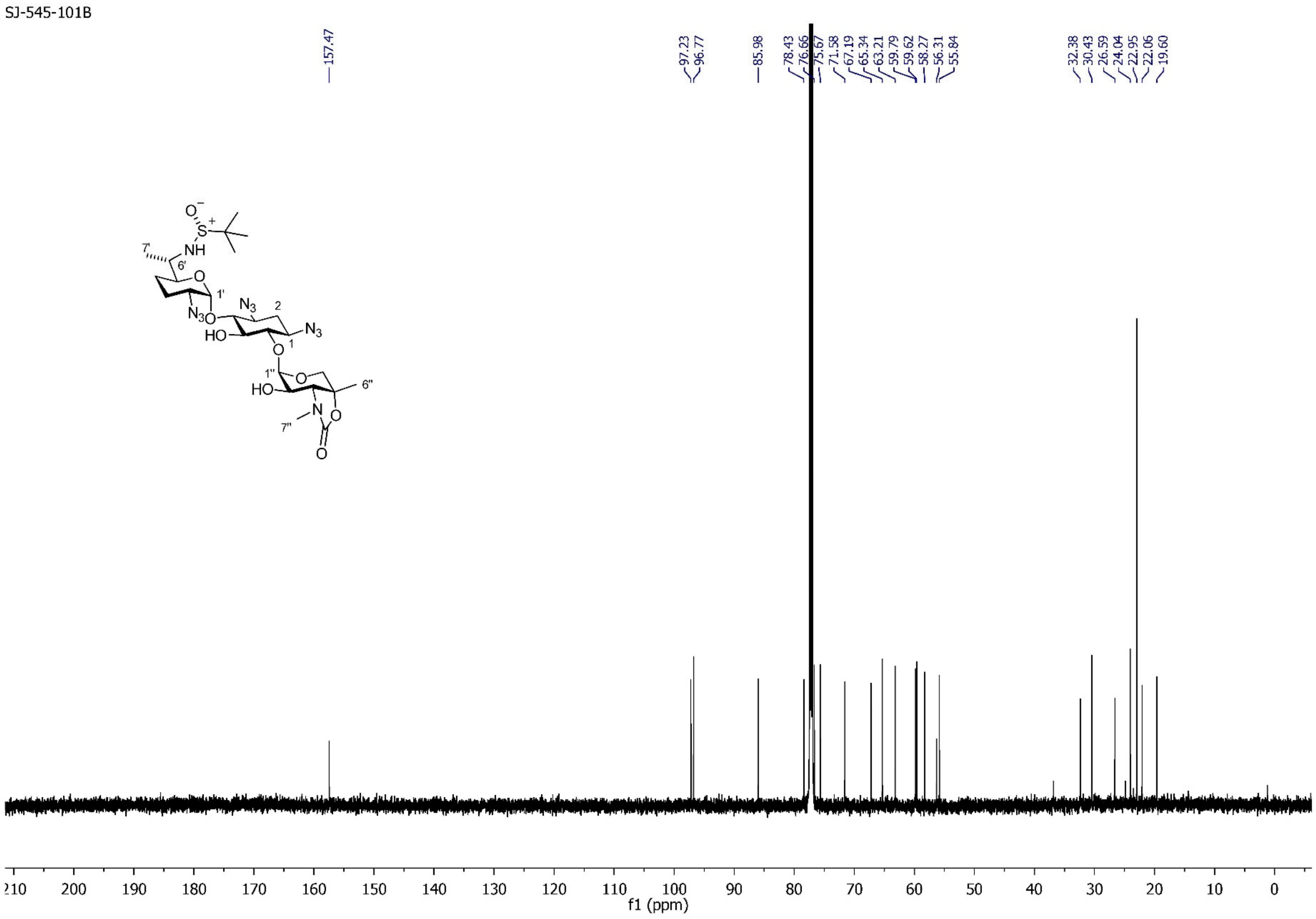

6′-(R_S)-*N*-(*tert*-Butylsulfinyl)amino-1,3,2′-tri(deamino)-1,3,2′-triazido-3′′-*N*,4′′-*O*-carbonyl-gentamicin C2a (^{1}H-^{1}H-COSY 600 MHz, $CDCl_3$) (18):

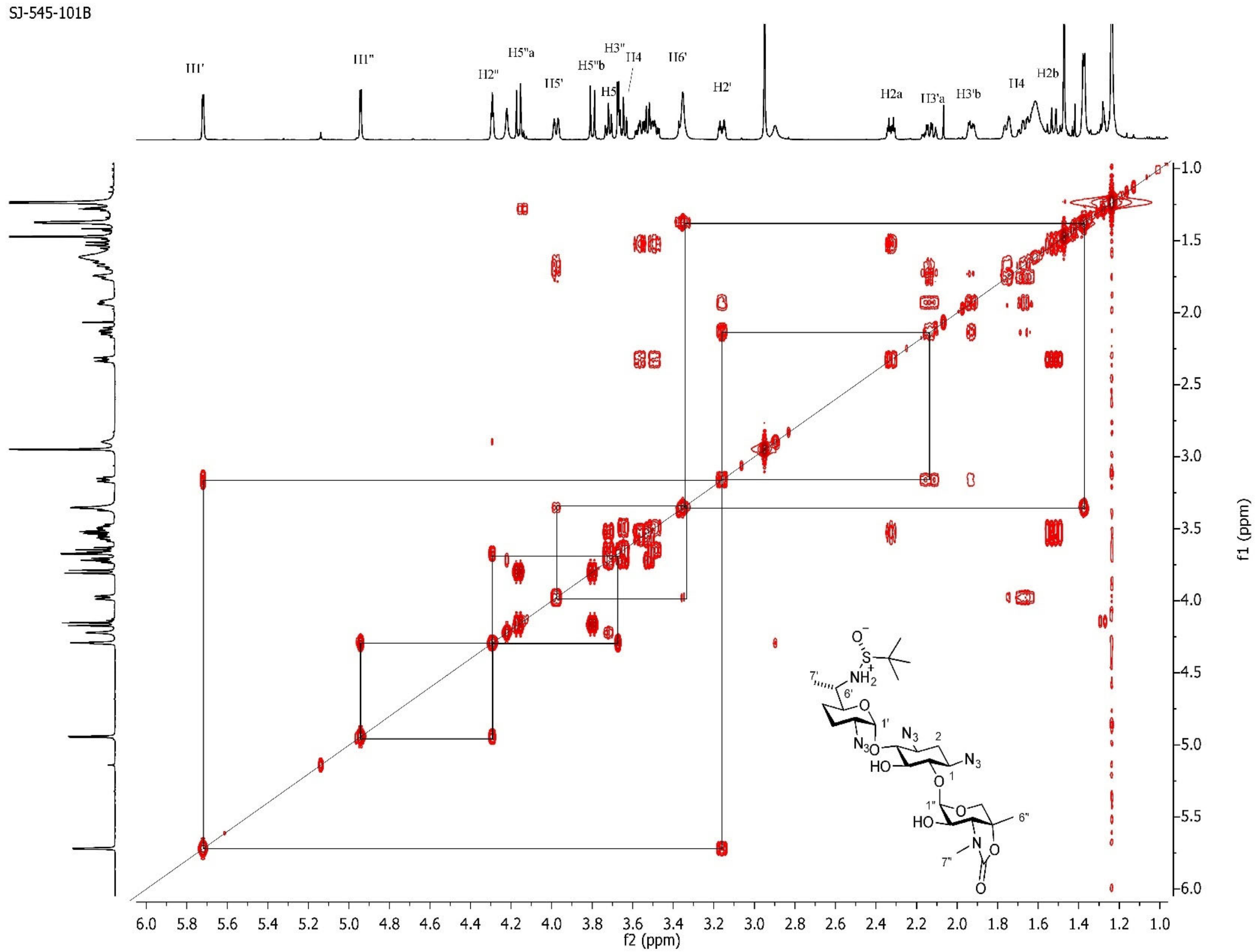

6′-(R_S)-*N*-(*tert*-Butylsulfinyl)amino-1,3,2′-tri(deamino)-1,3,2′-triazido-3″-*N*,4″-*O*-carbonyl-gentamicin C2a (^{1}H-^{13}C-HSQC 600 MHz, $CDCl_3$) (18):

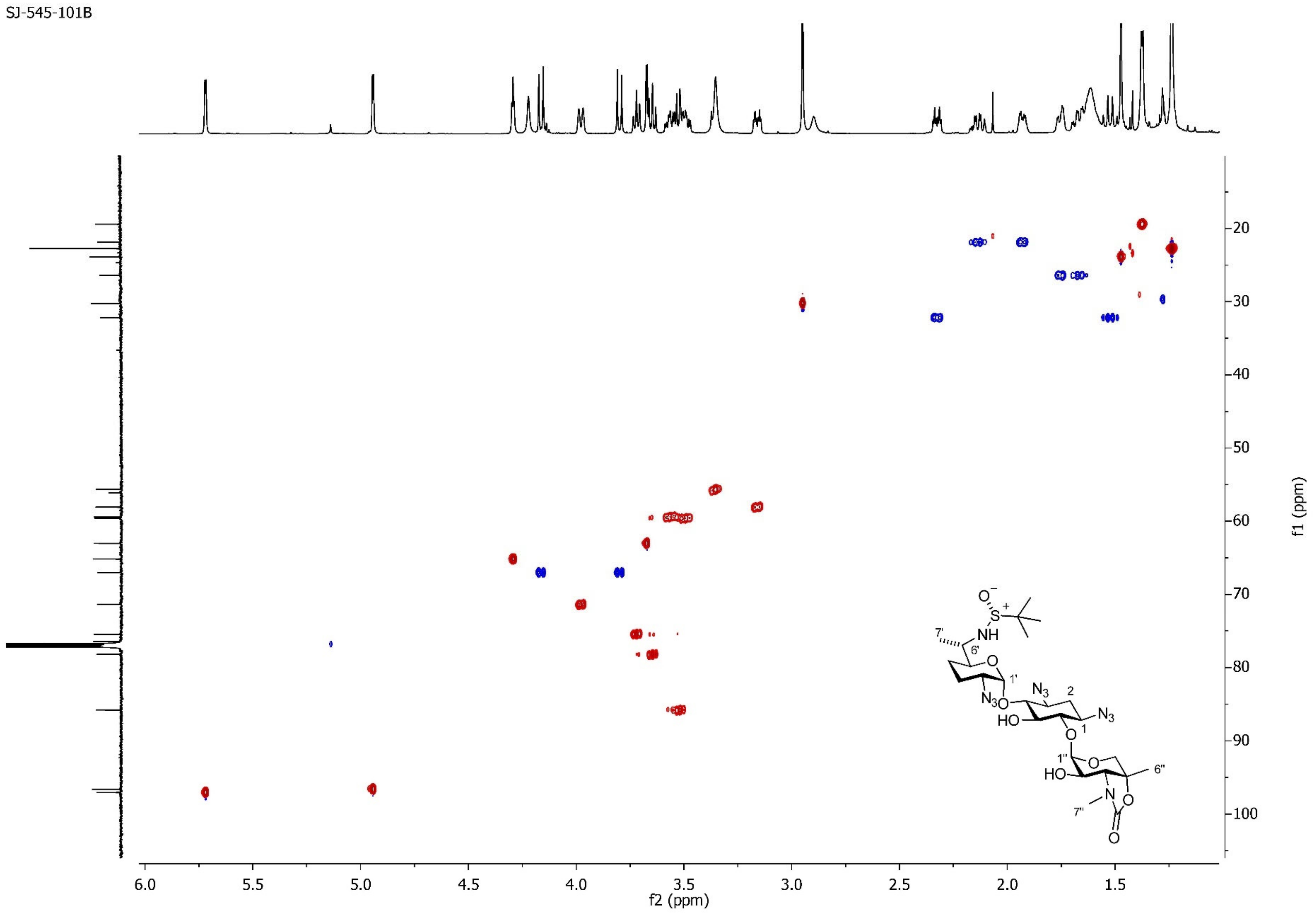

6′-(R_s)-*N*-(*tert*-Butylsulfinyl)amino-1,3,2′-tri(deamino)-1,3,2′-triazido-3″-*N*,4″-*O*-carbonyl-gentamicin C2a (^{1}H-^{13}C-HMBC 600 MHz, $CDCl_3$) (18):

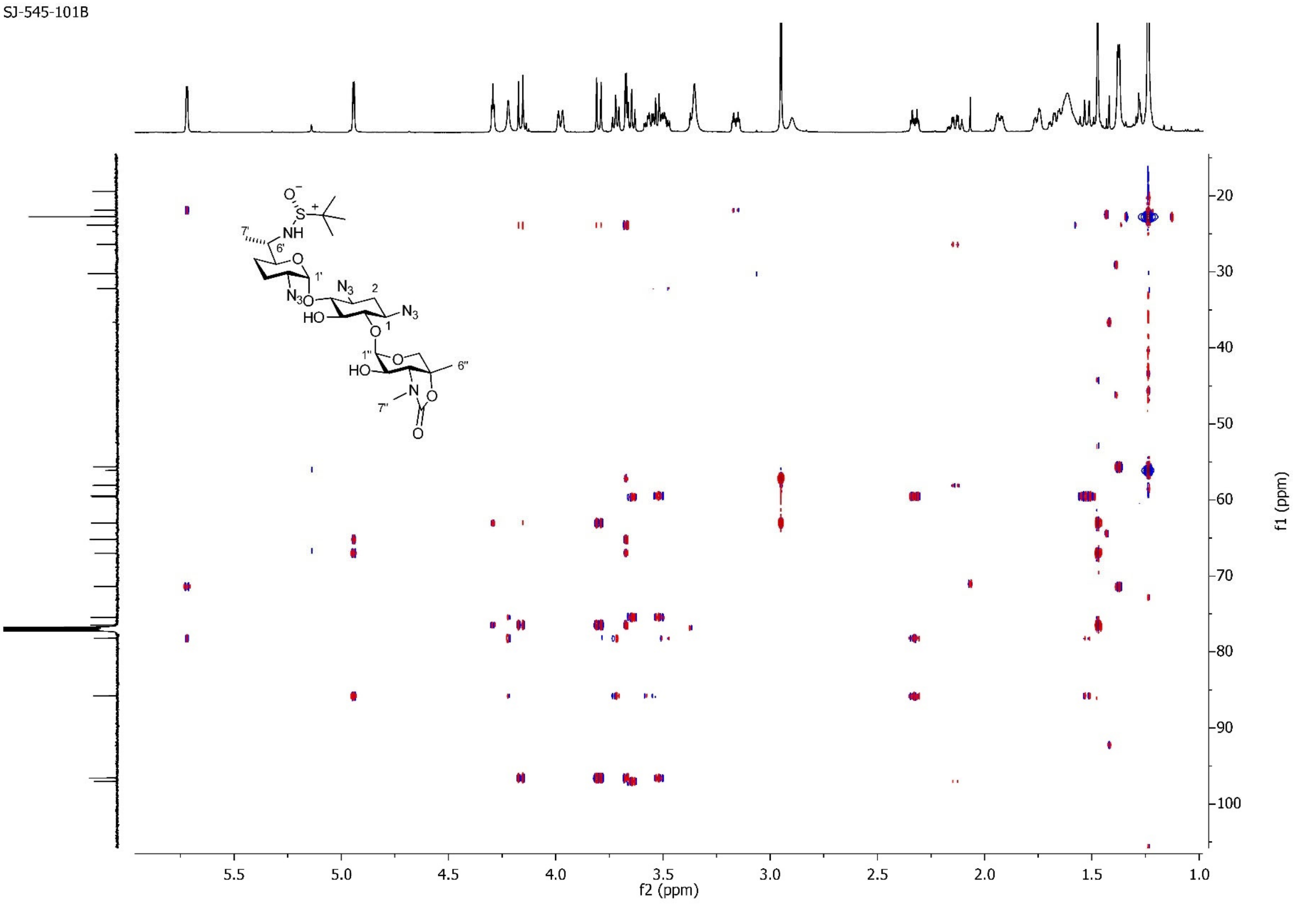

Gentamicin C2 (^{1}H 900 MHz, D_2O) (3):

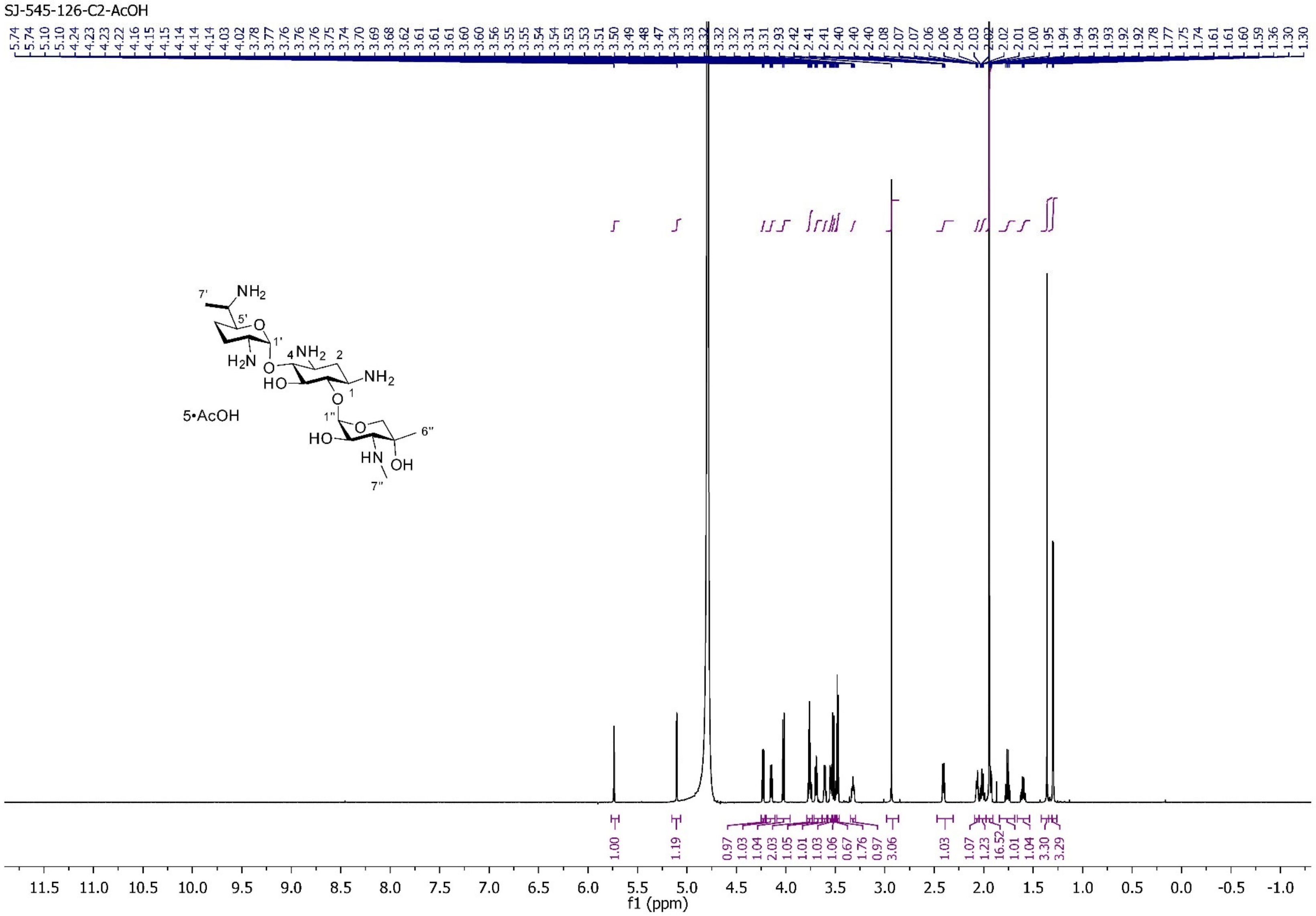

Gentamicin C2 (^{1}H 226 MHz, D_2O) (3):

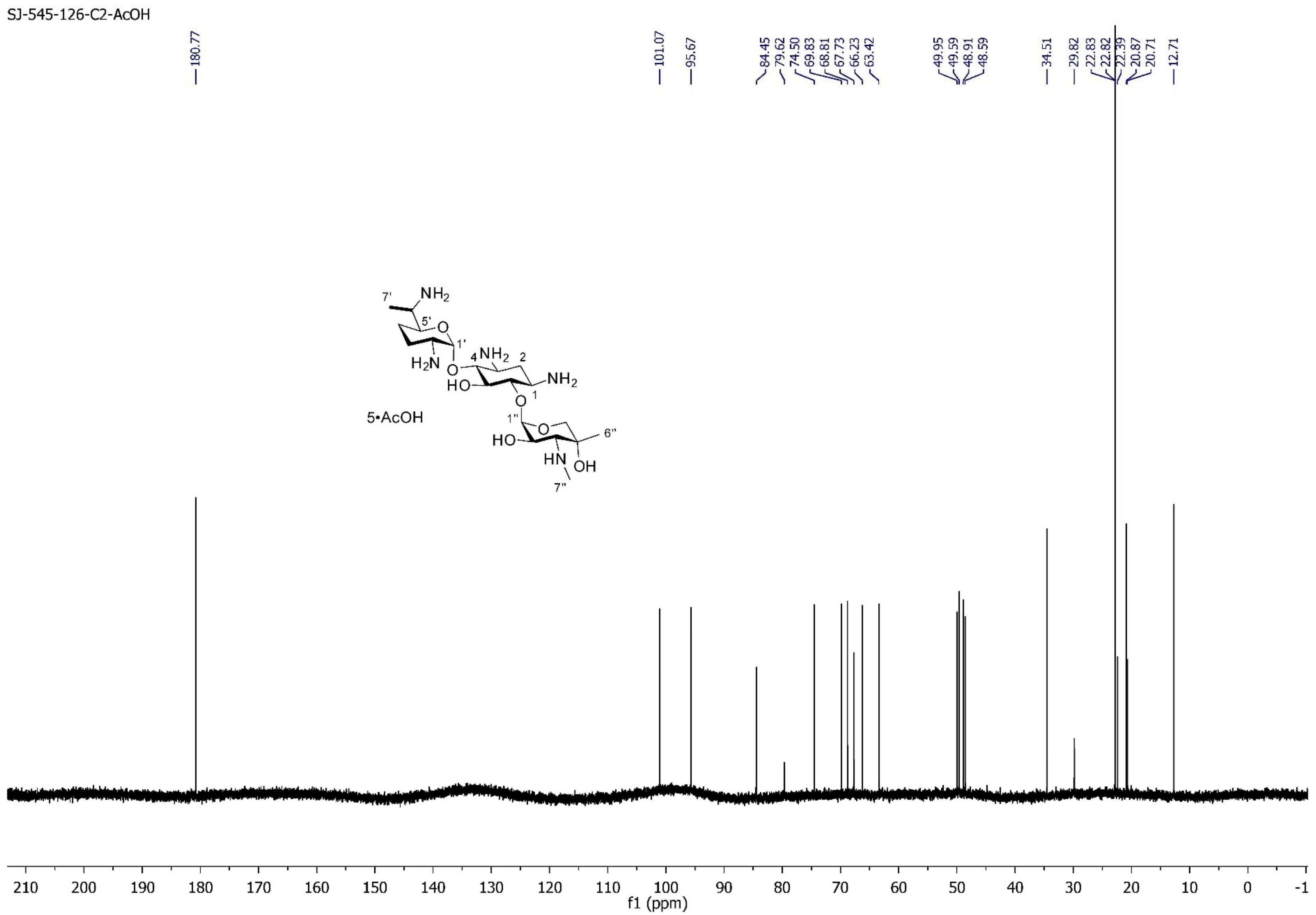

Gentamicin C2 (^1H-^1H-COSY 900 MHz, D_2O) (3):

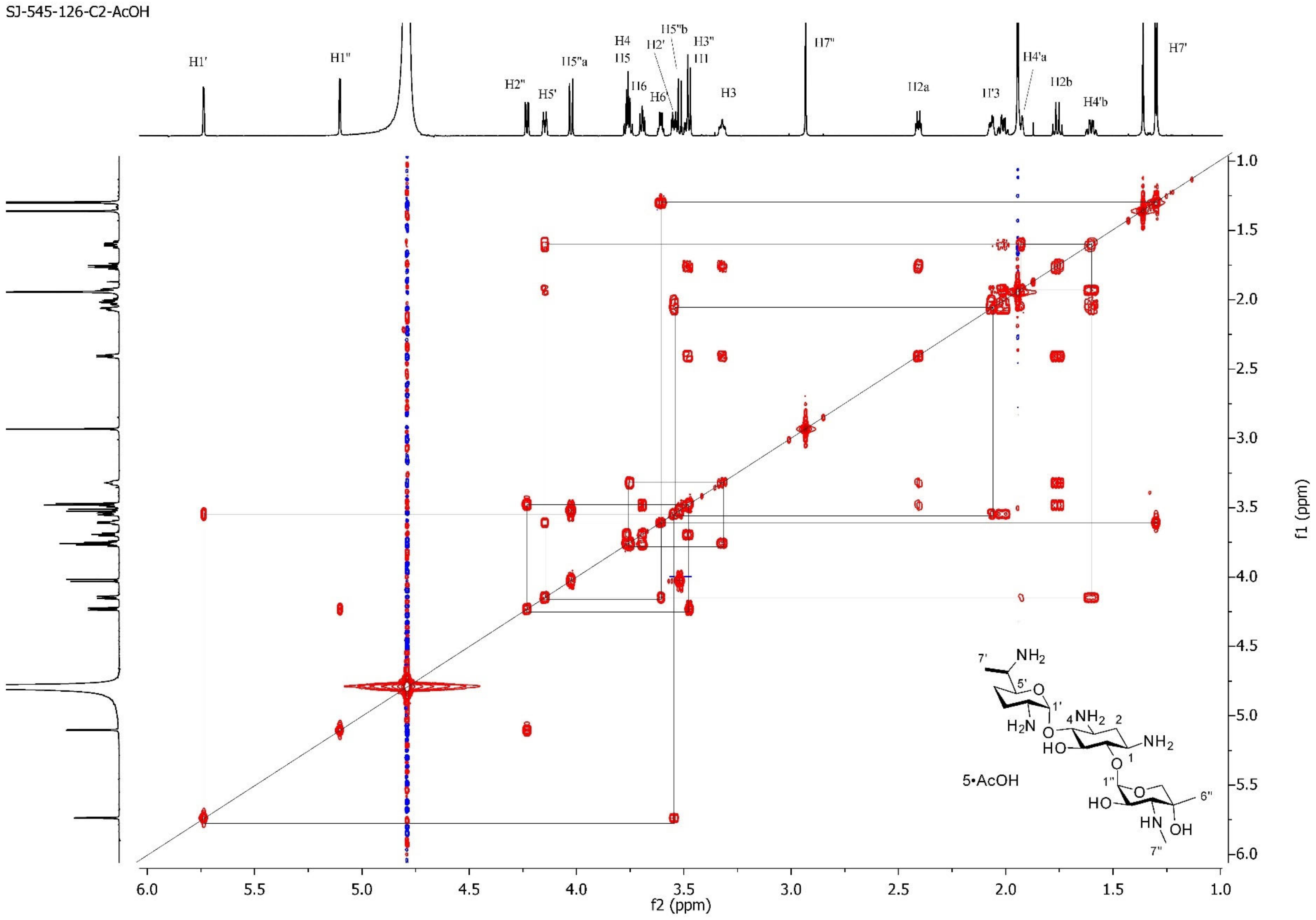

Gentamicin C2 (^1H-^{13}C-HSQC 900 MHz, D_2O) (3):

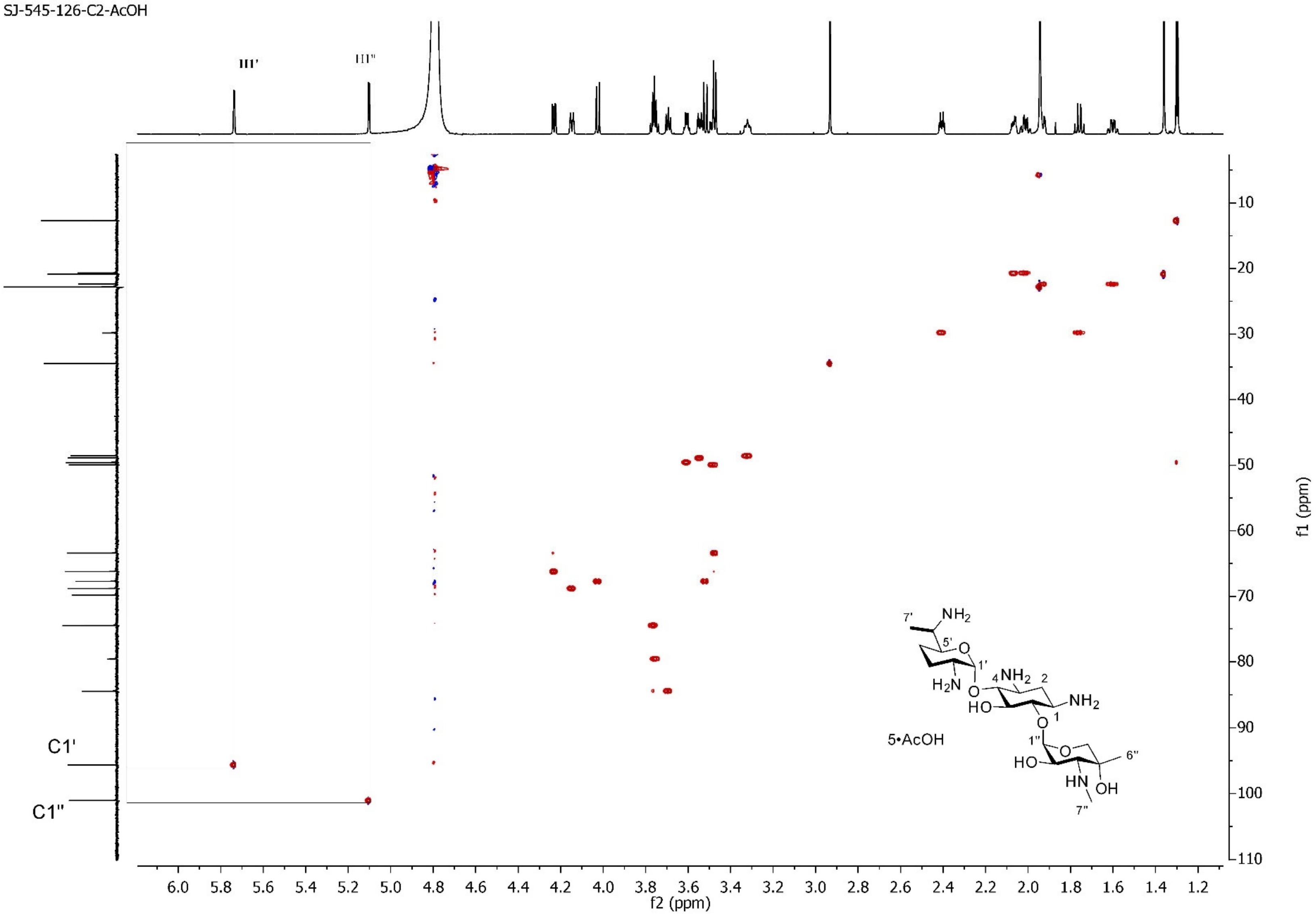

Gentamicin C2 (^{1}H-^{13}C-HMBC 900 MHz, D_2O) (3):

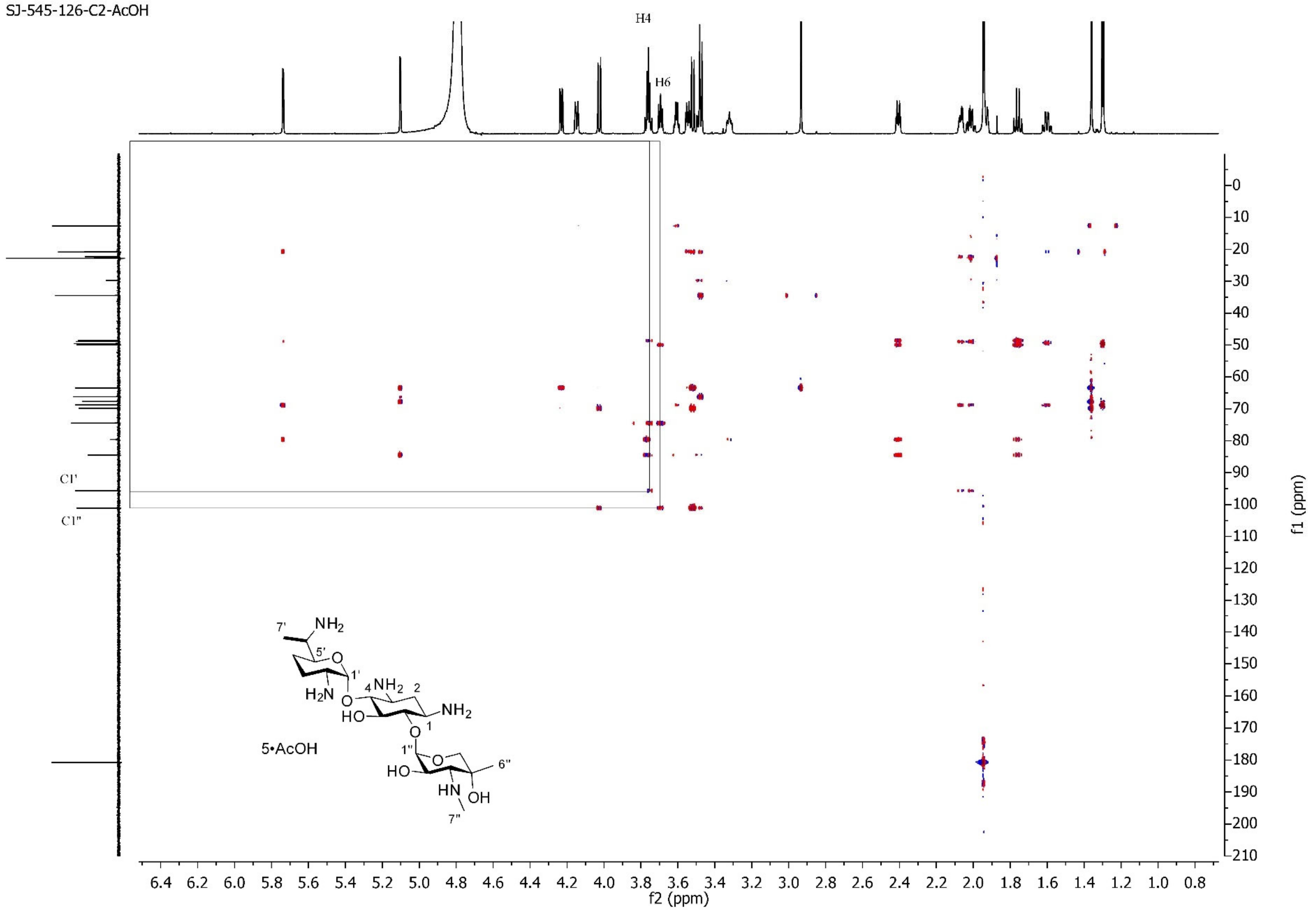

Gentamicin C2 (1D ROESY-H5′ 900 MHz, D_2O) (3):

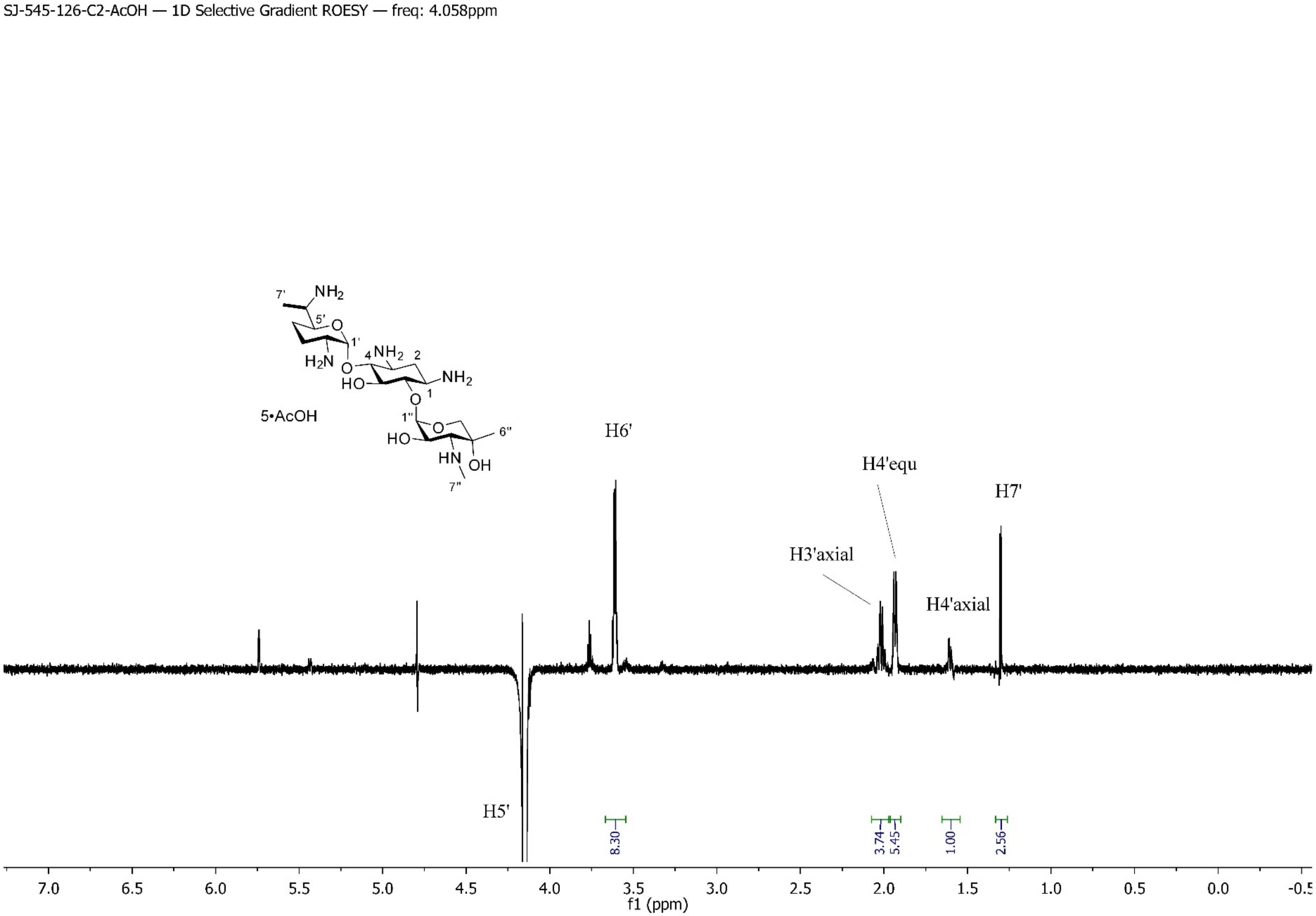

Gentamicin C2 (1D ROESY-H6′ 900 MHz, D_2O) (3):

SJ-545-126-C2-AcOH — 1D Selective Gradient ROESY — freq: 3.518ppm

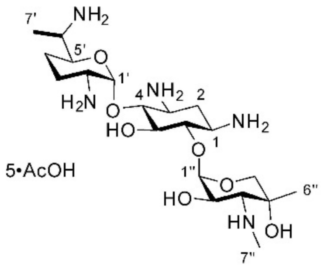

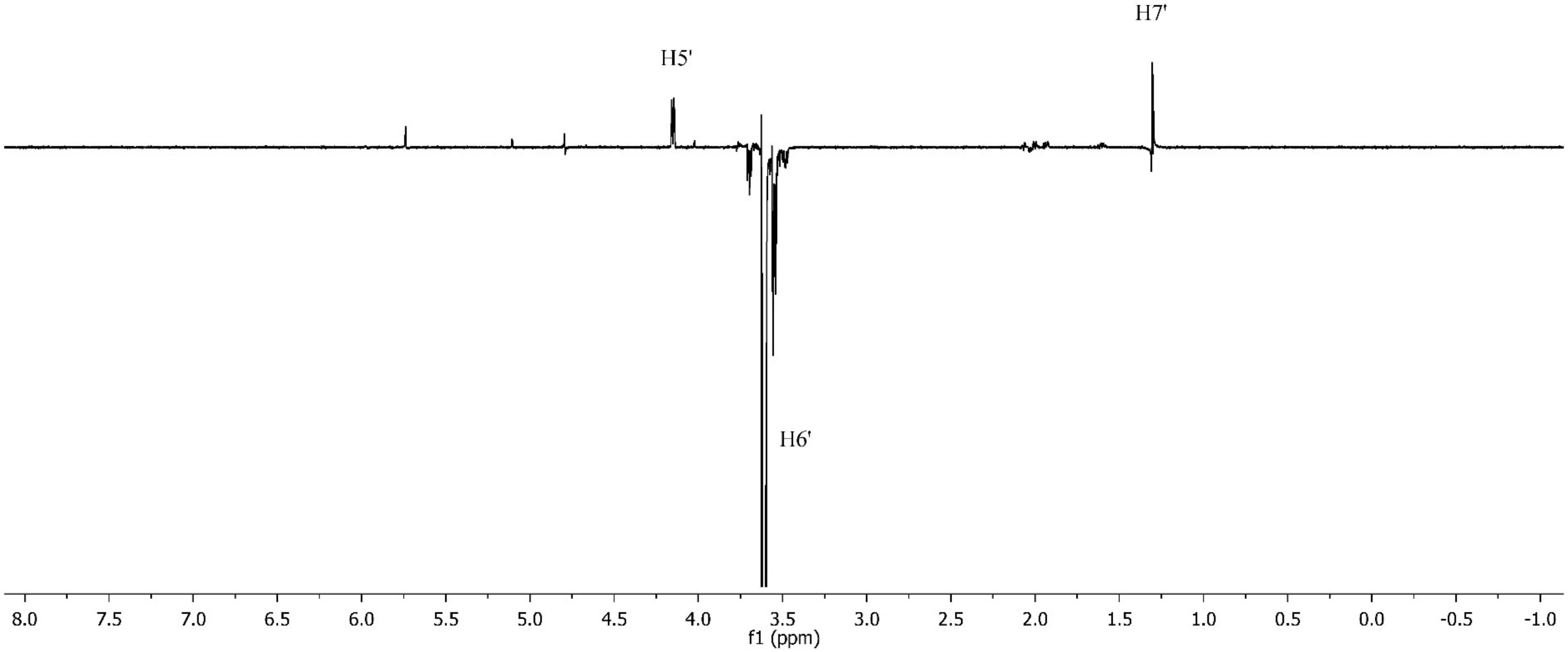

Gentamicin C2 (1D ROESY-H7′ 900 MHz, D_2O) (3):

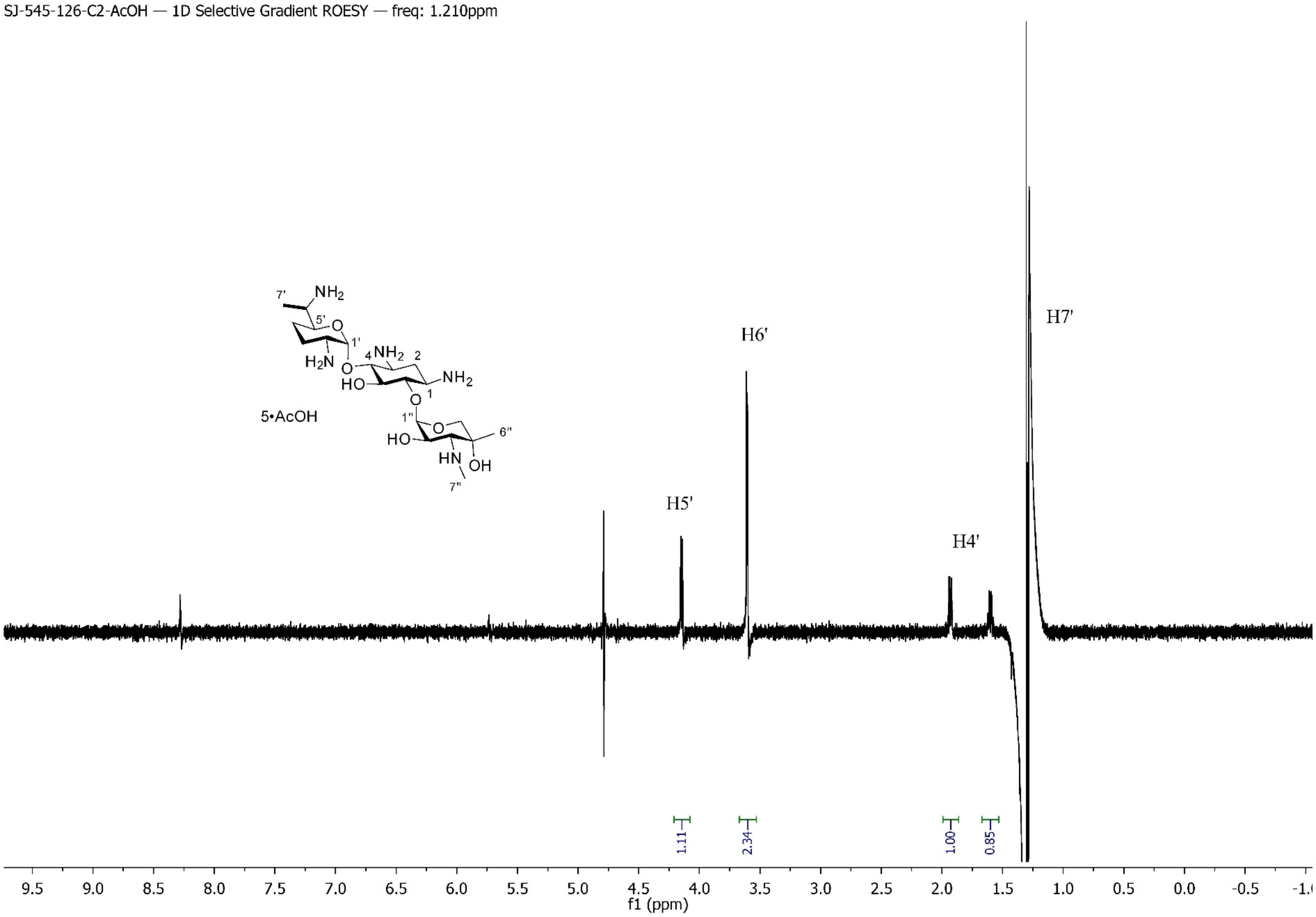

6′-(*S*R)-*N*-(*tert*-Butylsulfinyl)imino-1,3,2′-tri(deamino)-1,3,2′-triazido-3′′-*N*,4′′-*O*-carbonyl-gentamicin C1a (^{1}H 900 MHz, CDCl$_3$) (19):

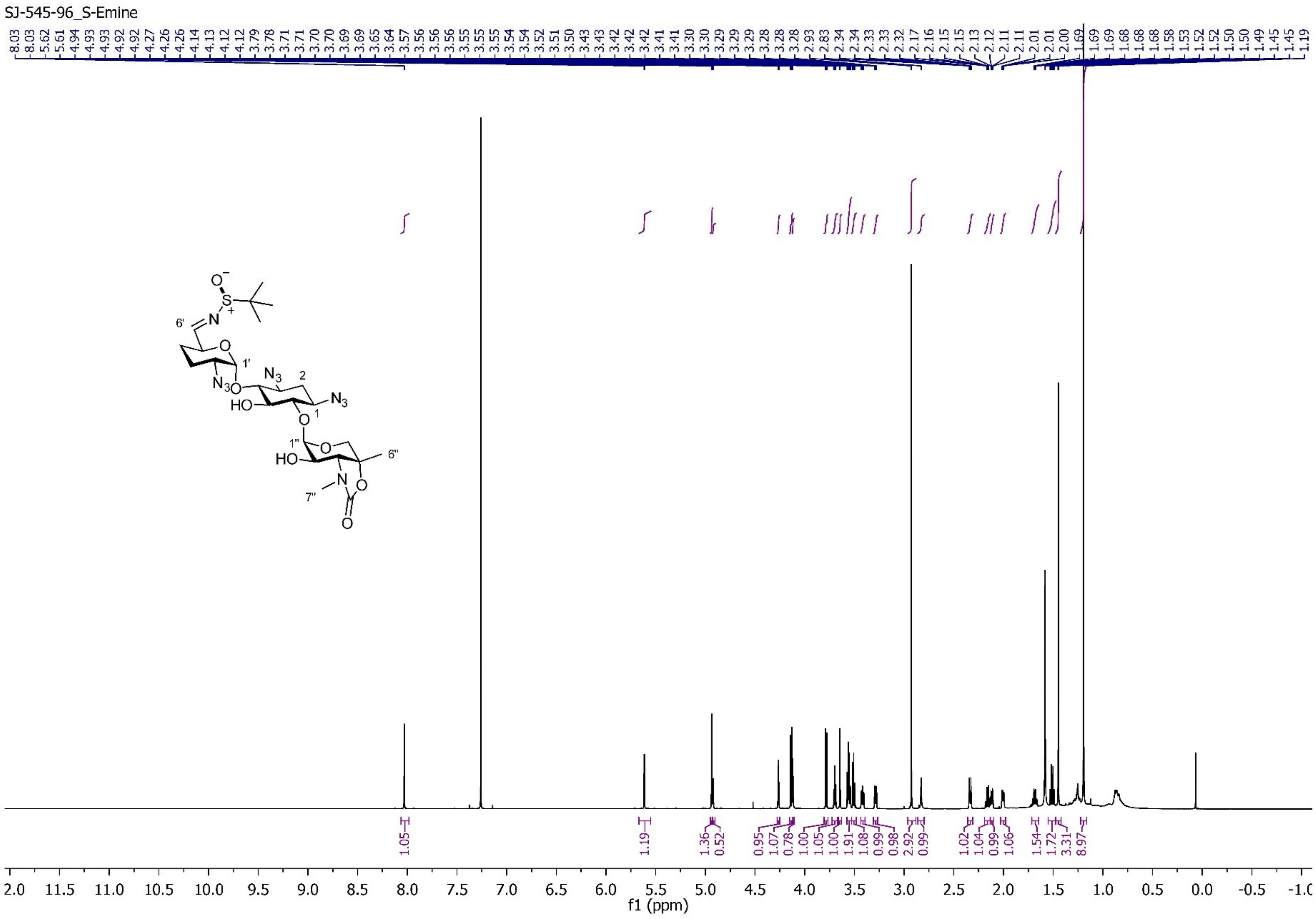

6′-(*S*R)-*N*-(*tert*-Butylsulfinyl)imino-1,3,2′-tri(deamino)-1,3,2′-triazido-3′′-*N*,4′′-*O*-carbonyl-gentamicin C1a (^{13}C 226 MHz, CDCl$_3$) (19):

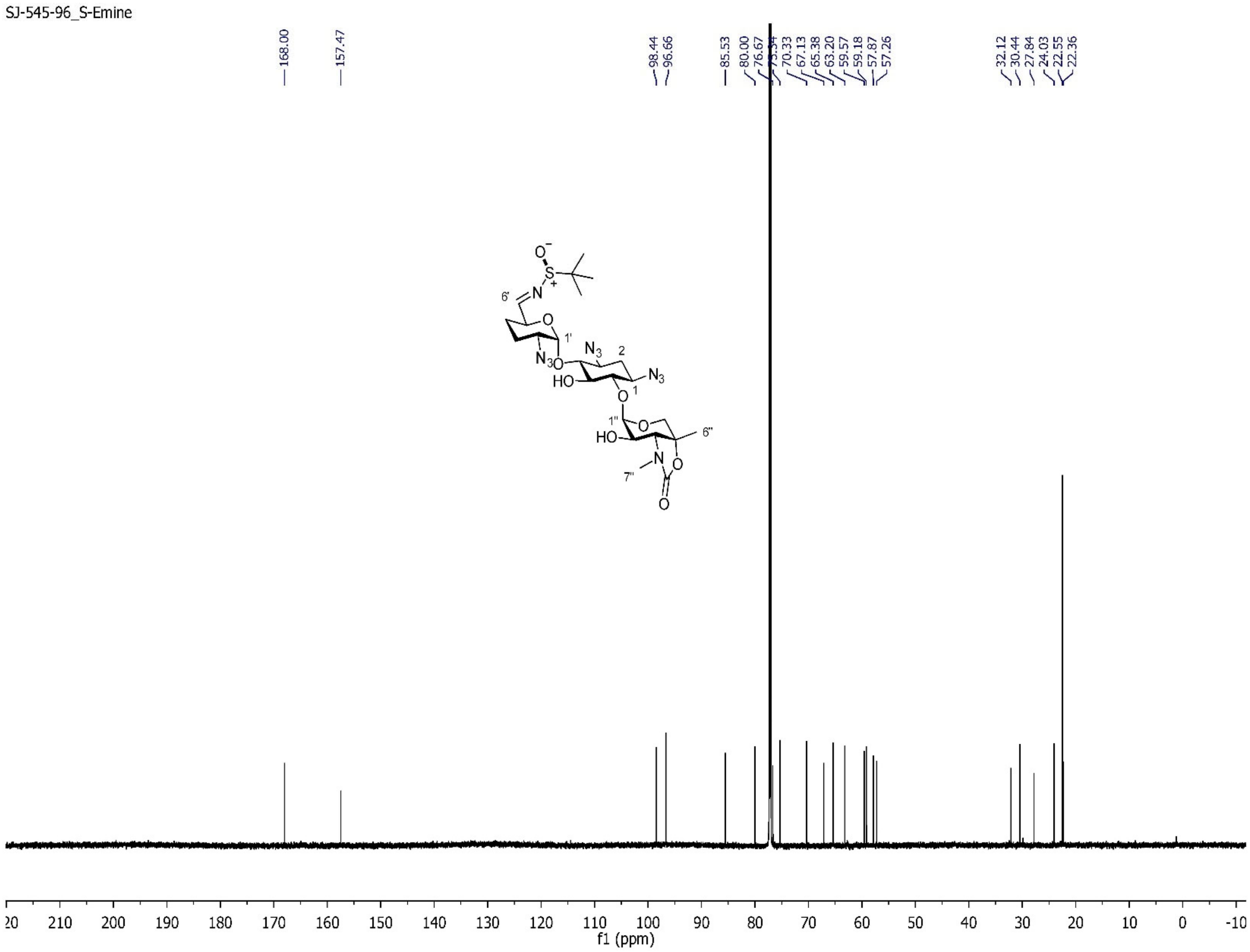

6′-(*S*$_R$)-*N*-(*tert*-Butylsulfinyl)imino-1,3,2′-tri(deamino)-1,3,2′-triazido-3′′-*N*,4′′-*O*-carbonyl-gentamicin C1a (^{1}H-^{1}H-COSY 900 MHz, CDCl$_3$) (19):

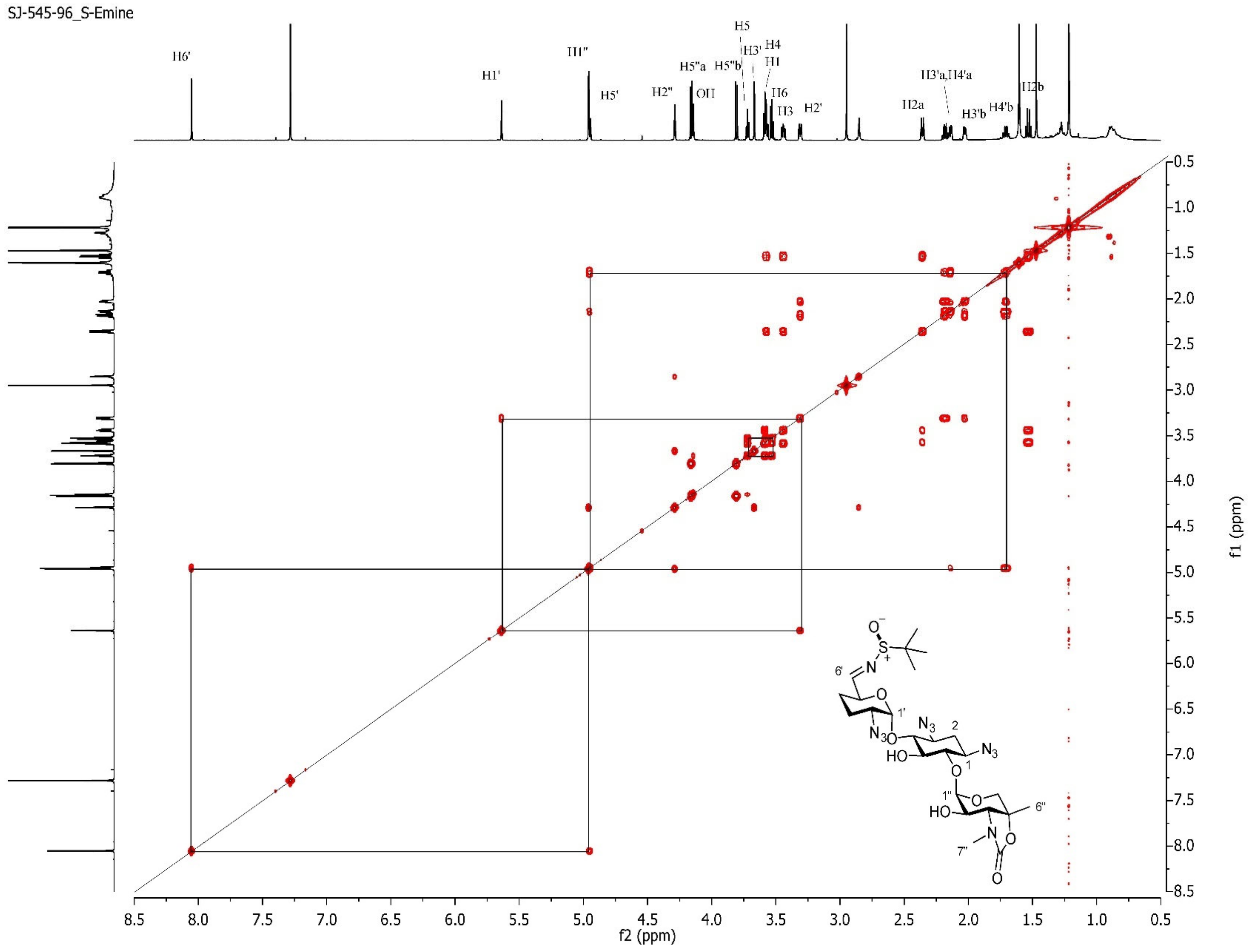

6′-(S_R)-*N*-(*tert*-Butylsulfinyl)imino-1,3,2′-tri(deamino)-1,3,2′-triazido-3′′-*N*,4′′-*O*-carbonyl-gentamicin C1a (^{1}H-^{13}C-HSQC 900 MHz, $CDCl_3$) (19):

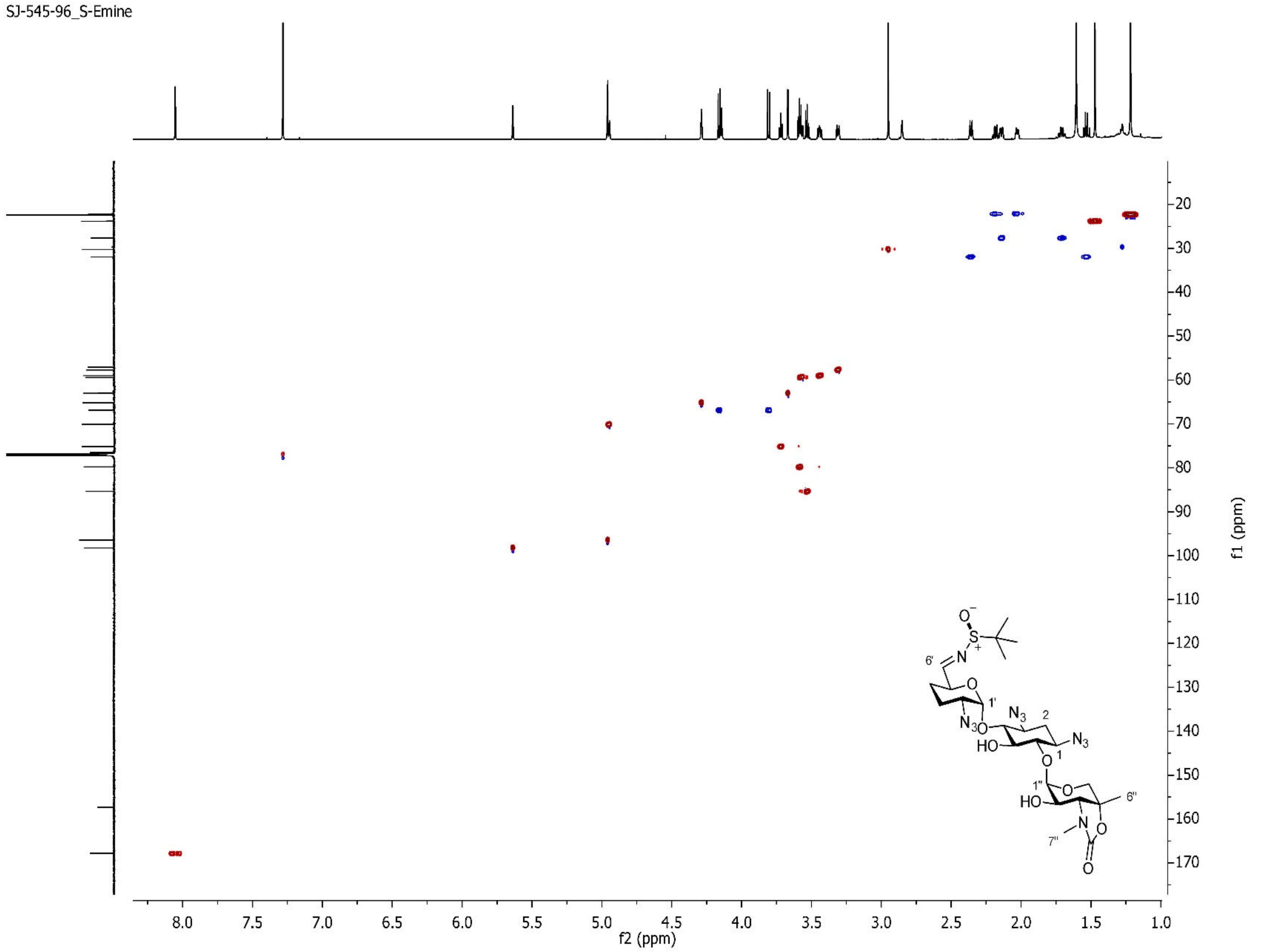

6′-(S_R)-*N*-(*tert*-Butylsulfinyl)imino-1,3,2′-tri(deamino)-1,3,2′-triazido-3′′-*N*,4′′-*O*-carbonyl-gentamicin C1a (^{1}H-^{13}C-HMBC 900 MHz, $CDCl_3$) (19):

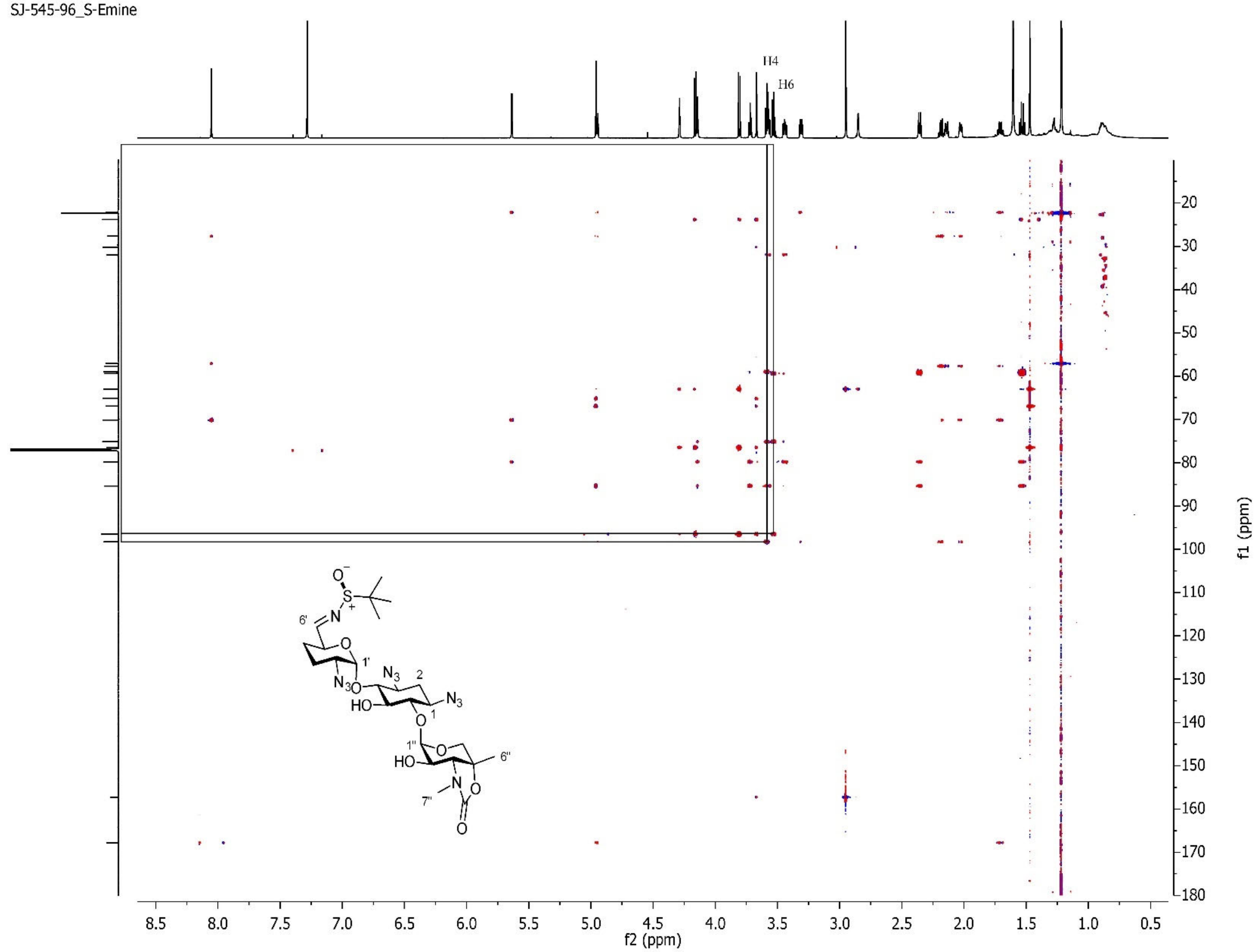

6′-(S_R)-*N*-(*tert*-Butylsulfinyl)amino-1,3,2′-tri(deamino)-1,3,2′-triazido-3″-*N*,4″-*O*-carbonyl-gentamicin C2 (^{1}H 900 MHz, CDCl$_3$) (20):

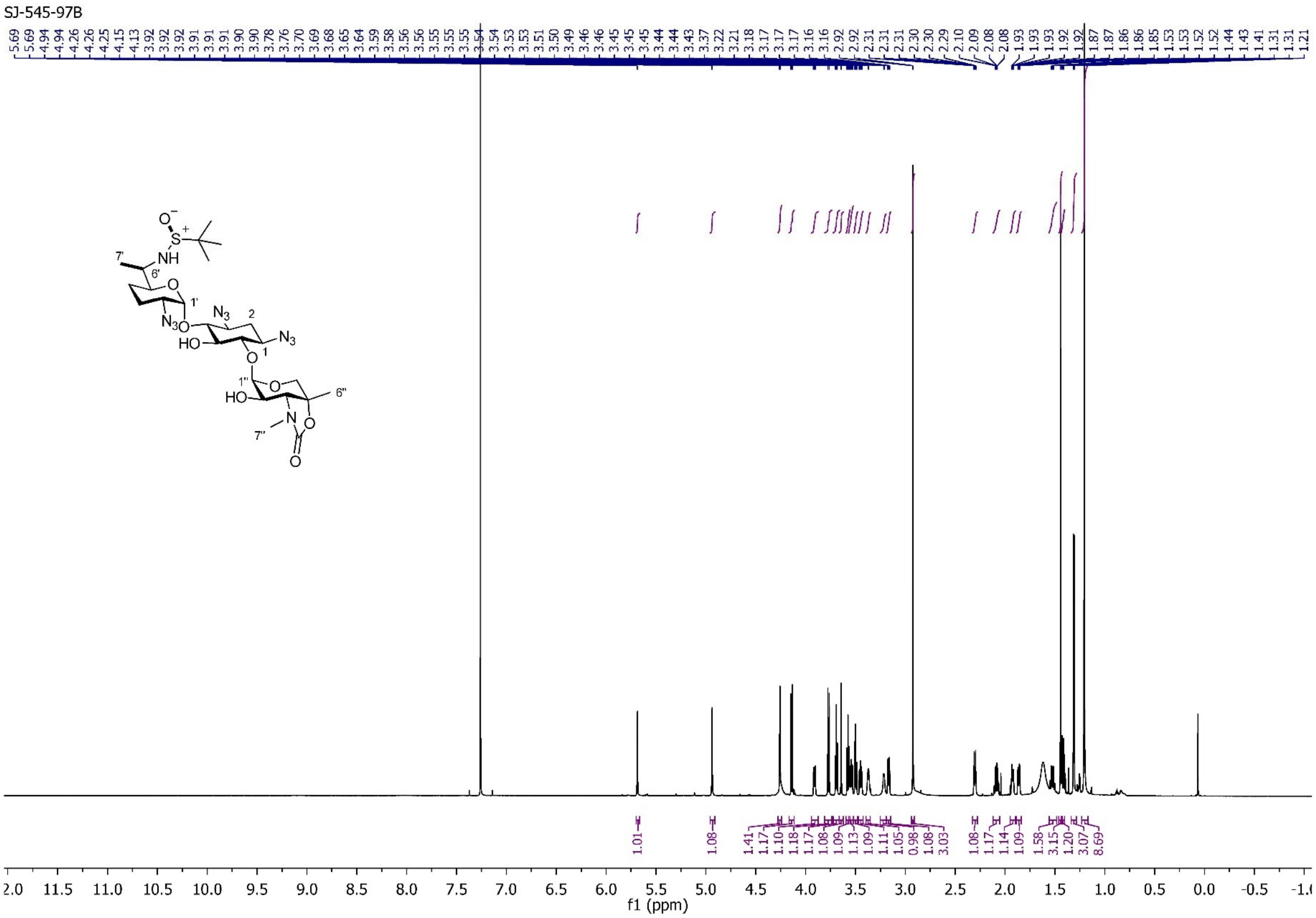

6′-(*S*R)-*N*-(*tert*-Butylsulfinyl)amino-1,3,2′-tri(deamino)-1,3,2′-triazido-3′′-*N*,4′′-*O*-carbonyl-gentamicin C2 (^{13}C 226 MHz, CDCl$_3$) (20):

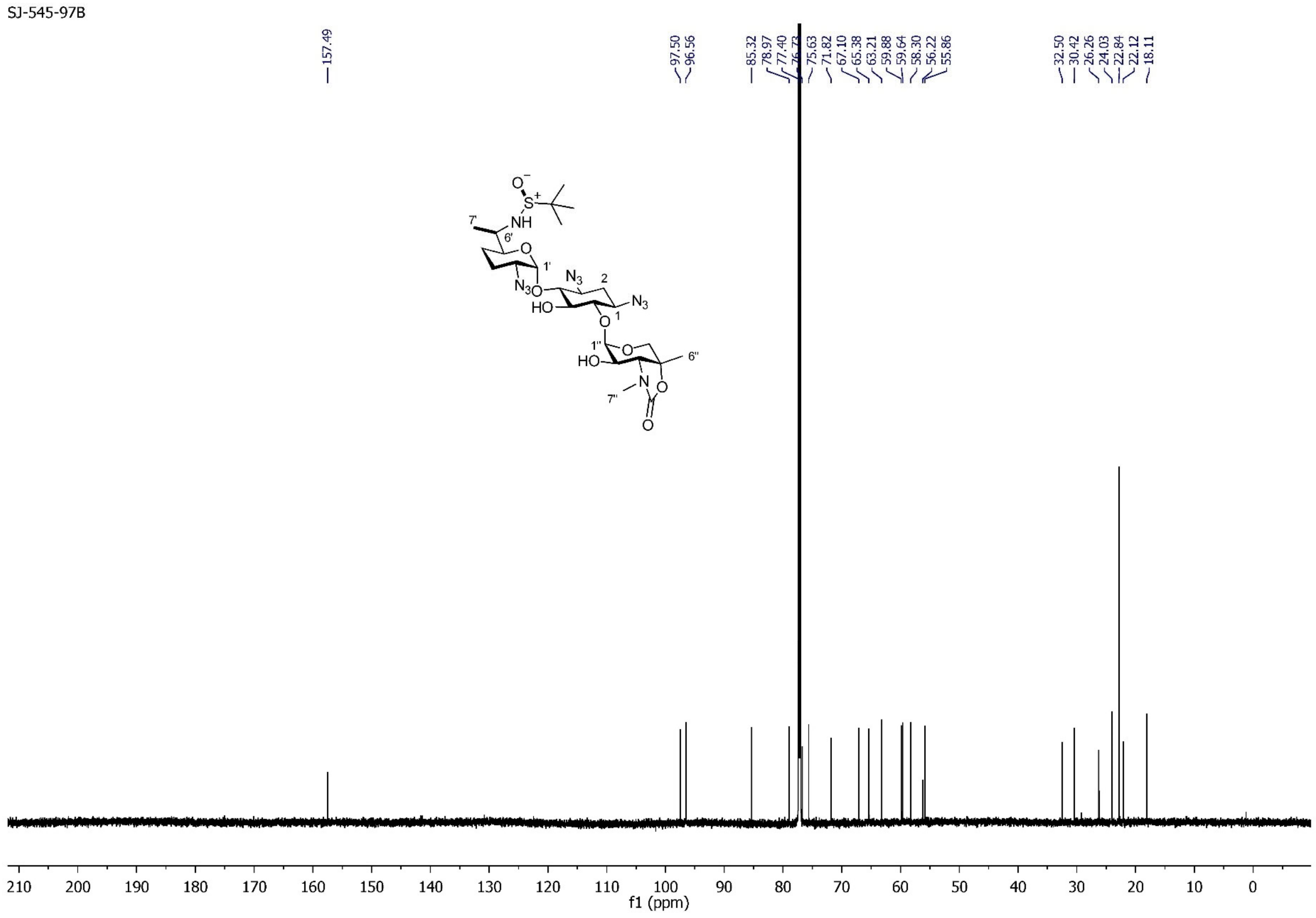

6′-(S_R)-*N*-(*tert*-Butylsulfinyl)amino-1,3,2′-tri(deamino)-1,3,2′-triazido-3′′-*N*,4′′-*O*-carbonyl-gentamicin C2 (^{1}H-^{1}H-COSY 900 MHz, $CDCl_3$) (20):

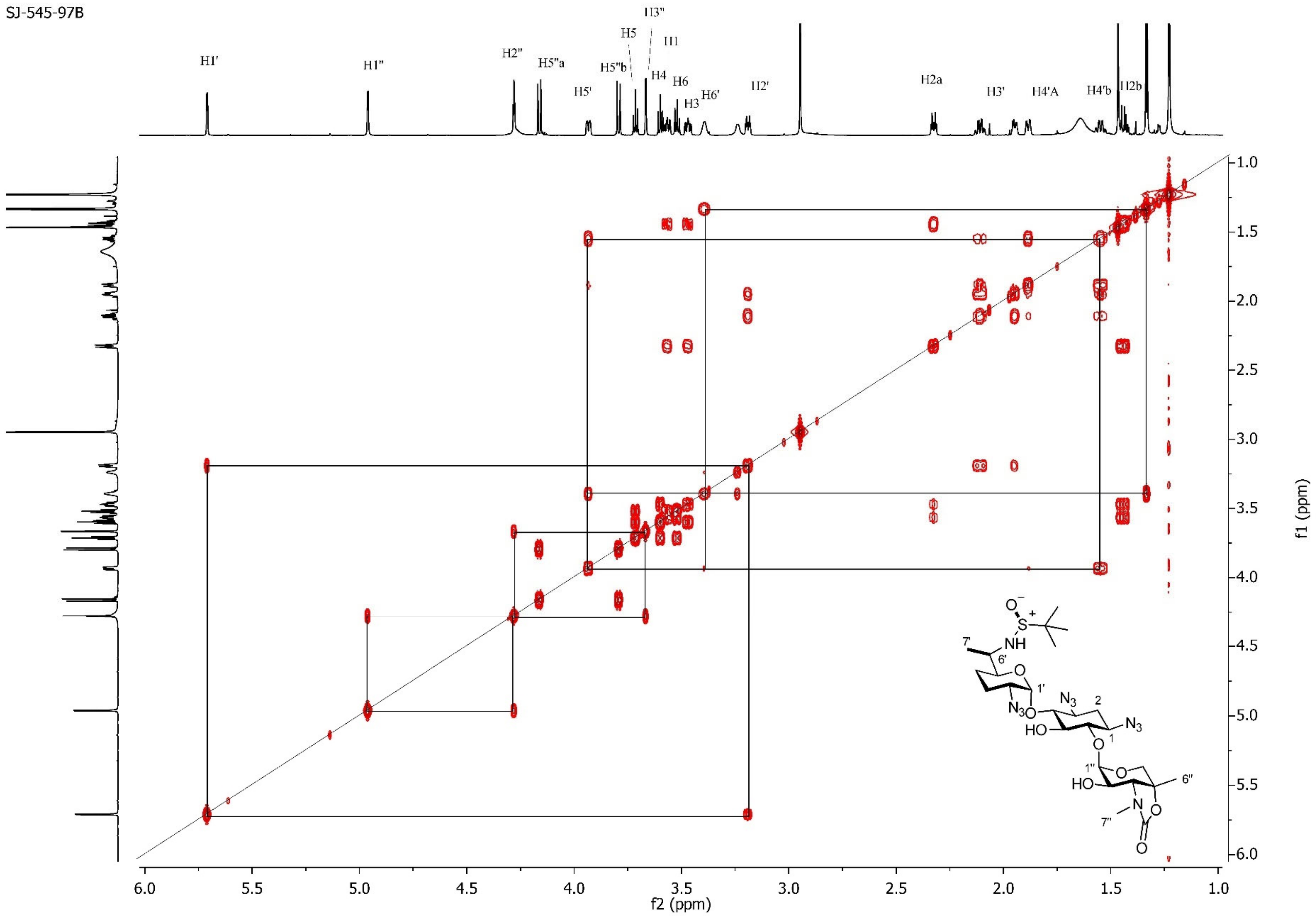

6′-(S_R)-*N*-(*tert*-Butylsulfinyl)amino-1,3,2′-tri(deamino)-1,3,2′-triazido-3′′-*N*,4′′-*O*-carbonyl-gentamicin C2 (^1H-^{13}C-HSQC 900 MHz, $CDCl_3$) (20):

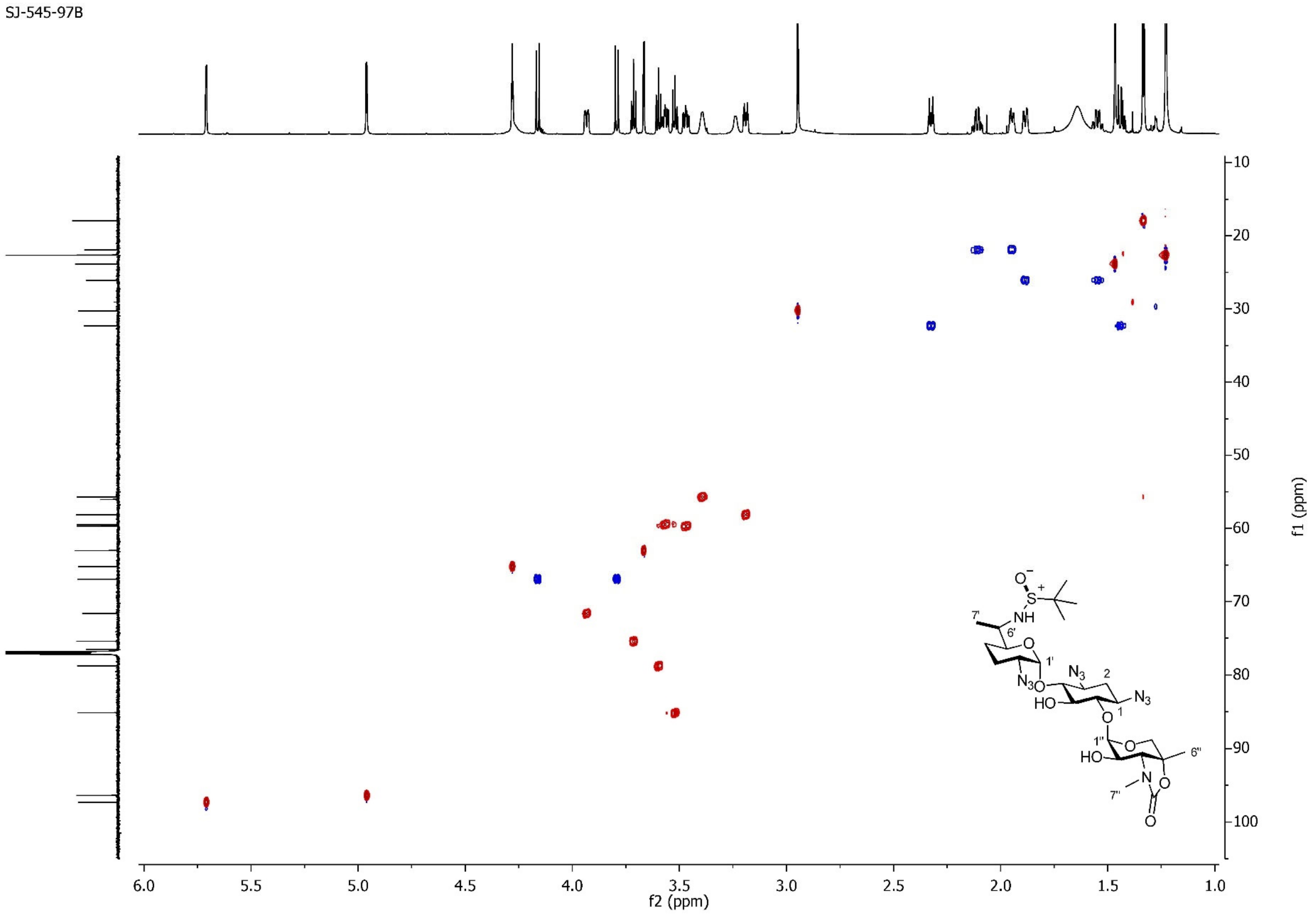

6′-(*S*R)-*N*-(*tert*-Butylsulfinyl)amino-1,3,2′-tri(deamino)-1,3,2′-triazido-3′′-*N*,4′′-*O*-carbonyl-gentamicin C2 (^{1}H-^{13}C-HMBC 900 MHz, $CDCl_3$) (20):

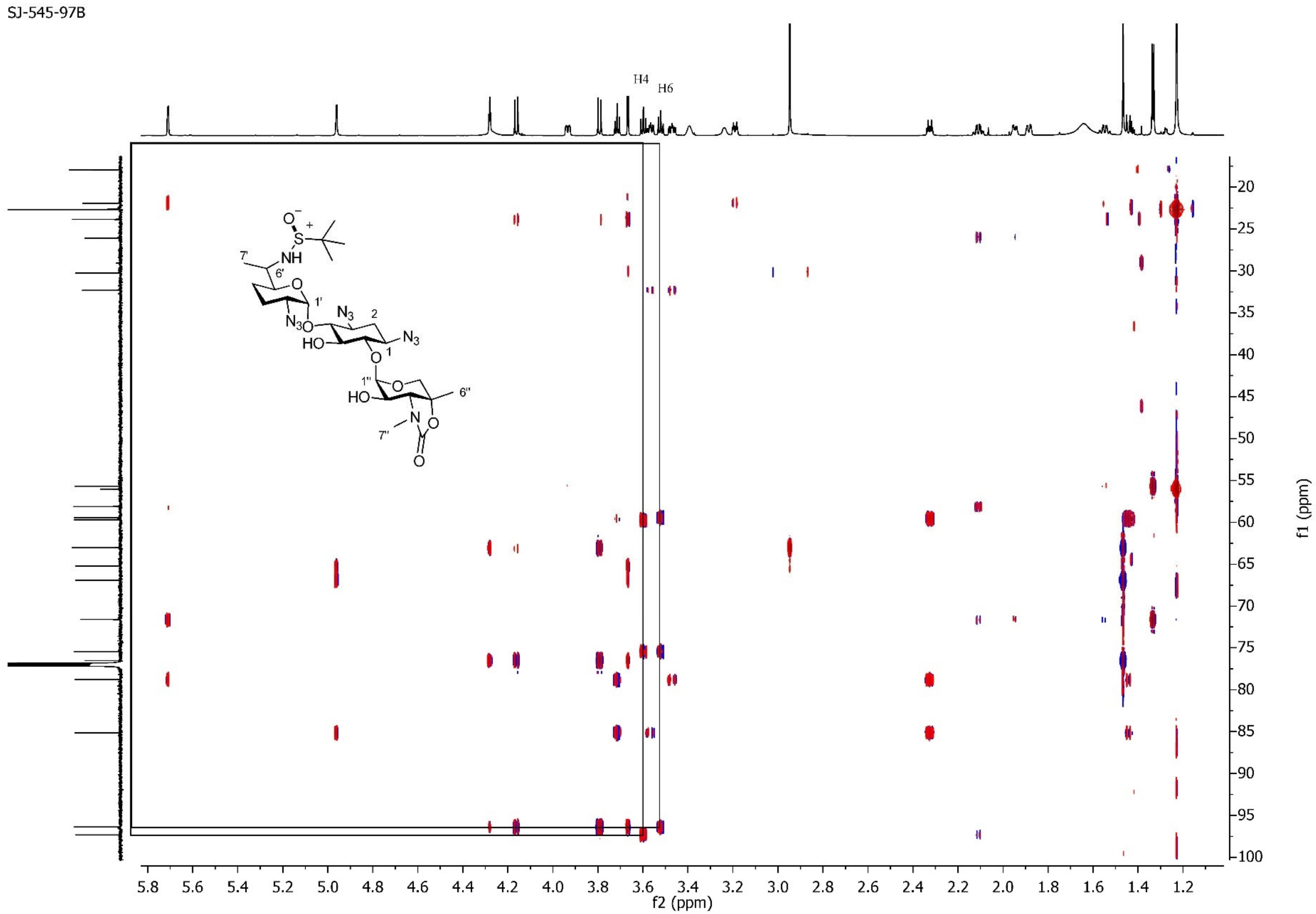

6′-(S_R)-*N*-(*tert*-Butylsulfinyl)amino-1,3,2′-tri(deamino)-1,3,2′-triazido-3′′-*N*,4′′-*O*-carbonyl-gentamicin C2a (^{1}H 900 MHz, $CDCl_3$) (21):

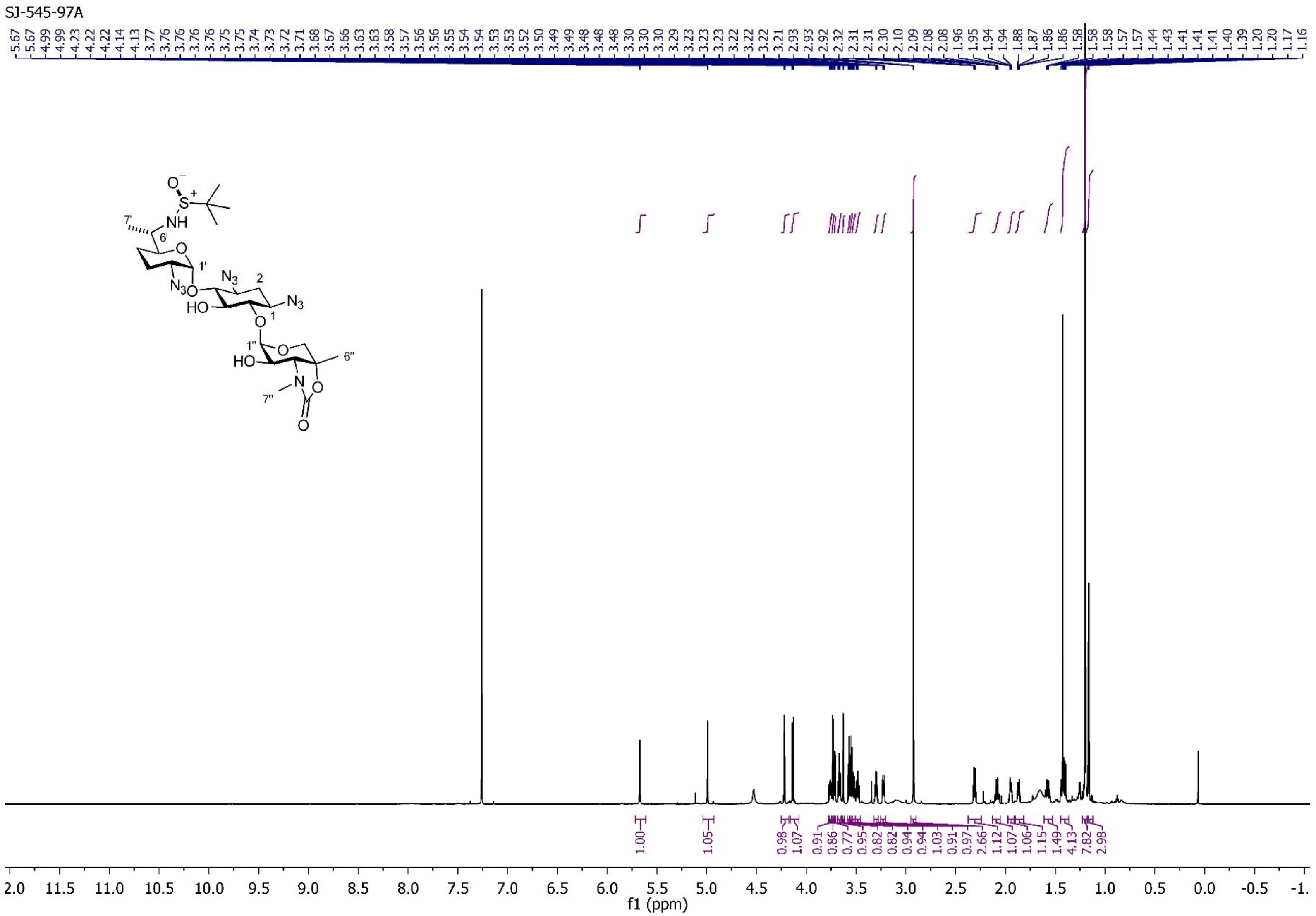

6′-(S_R)-*N*-(*tert*-Butylsulfinyl)amino-1,3,2′-tri(deamino)-1,3,2′-triazido-3′′-*N*,4′′-*O*-carbonyl-gentamicin C2a (^{13}C 226 MHz, $CDCl_3$) (21):

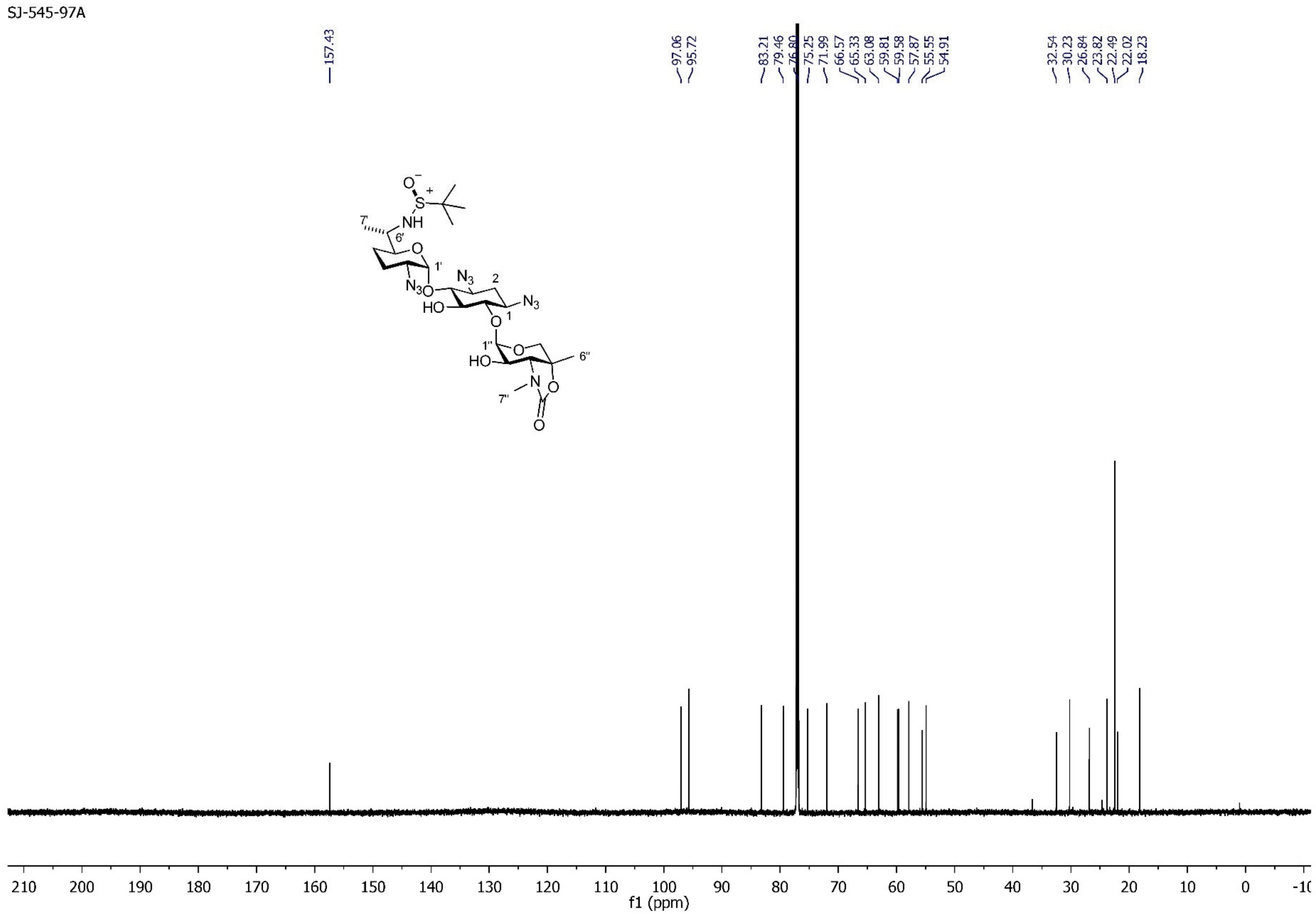

6′-(S_R)-*N*-(*tert*-Butylsulfinyl)amino-1,3,2′-tri(deamino)-1,3,2′-triazido-3″-*N*,4″-*O*-carbonyl-gentamicin C2a (^1H-^1H-COSY 900 MHz, $CDCl_3$) (21):

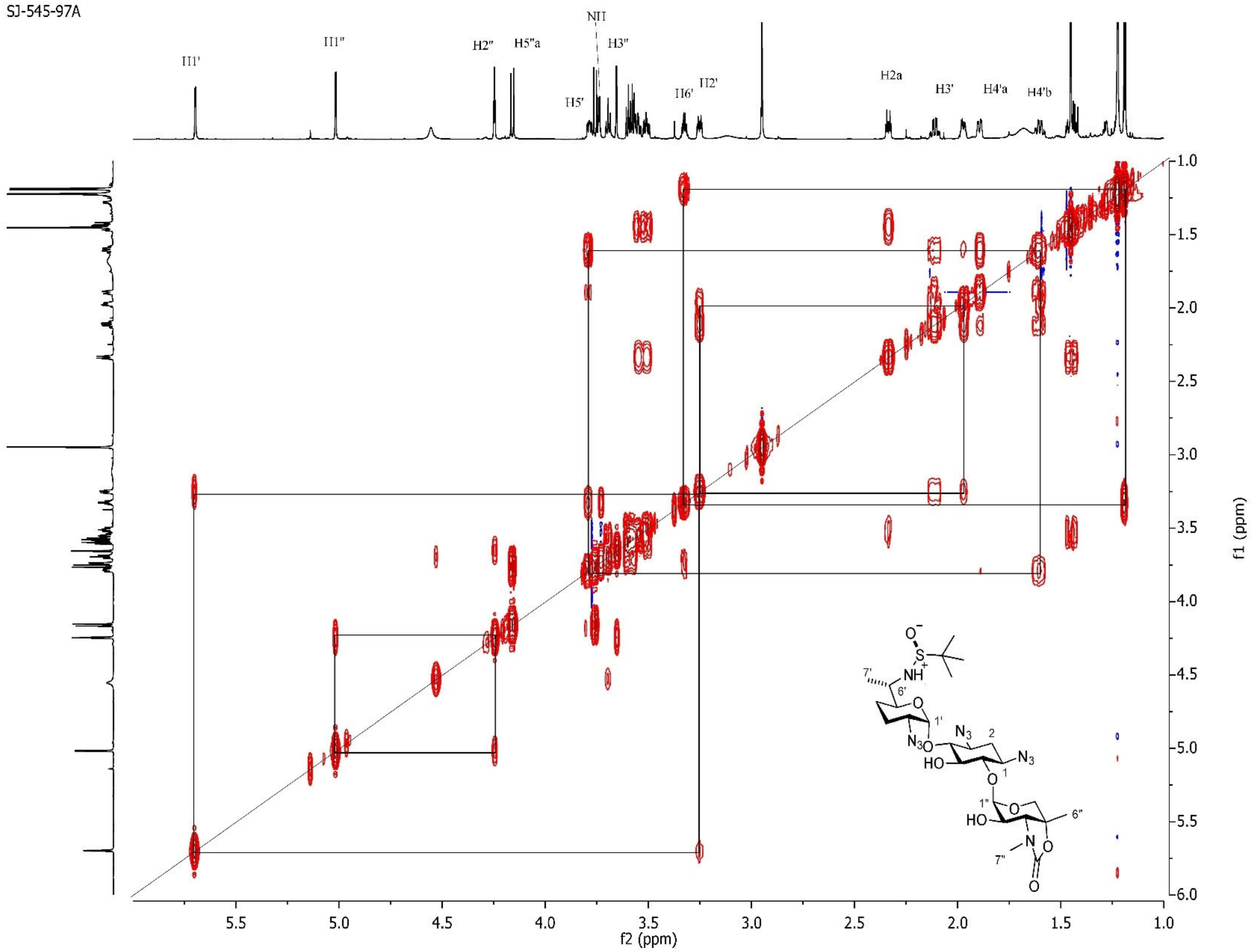

6′-(*S*R)-*N*-(*tert*-Butylsulfinyl)amino-1,3,2′-tri(deamino)-1,3,2′-triazido-3′′-*N*,4′′-*O*-carbonyl-gentamicin C2a (^{1}H-^{13}C-HSQC 900 MHz, $CDCl_3$) (21):

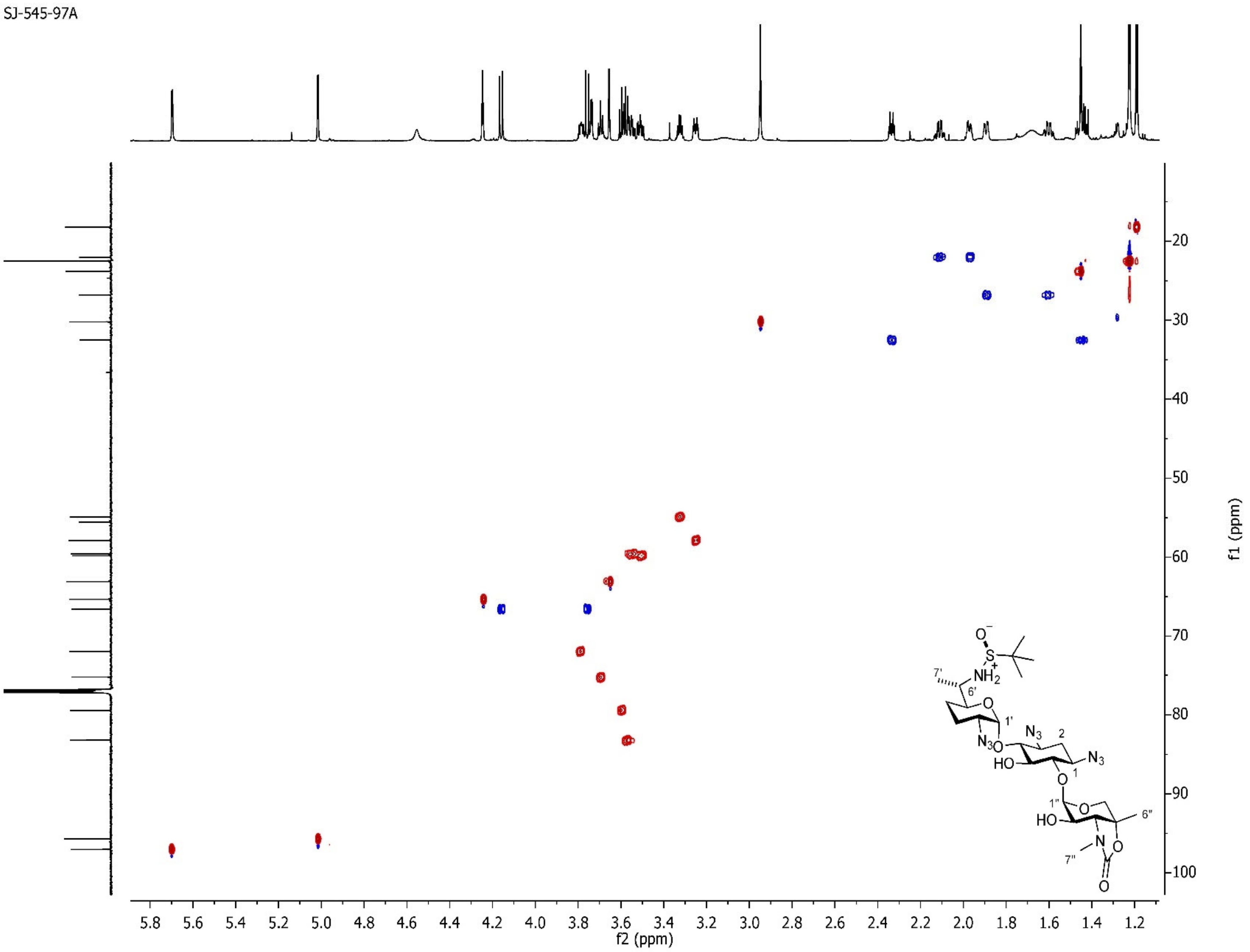

6′-(S_R)-*N*-(*tert*-Butylsulfinyl)amino-1,3,2′-tri(deamino)-1,3,2′-triazido-3″-*N*,4″-*O*-carbonyl-gentamicin C2a (^{1}H-^{13}C-HMBC 900 MHz, $CDCl_3$) (21):

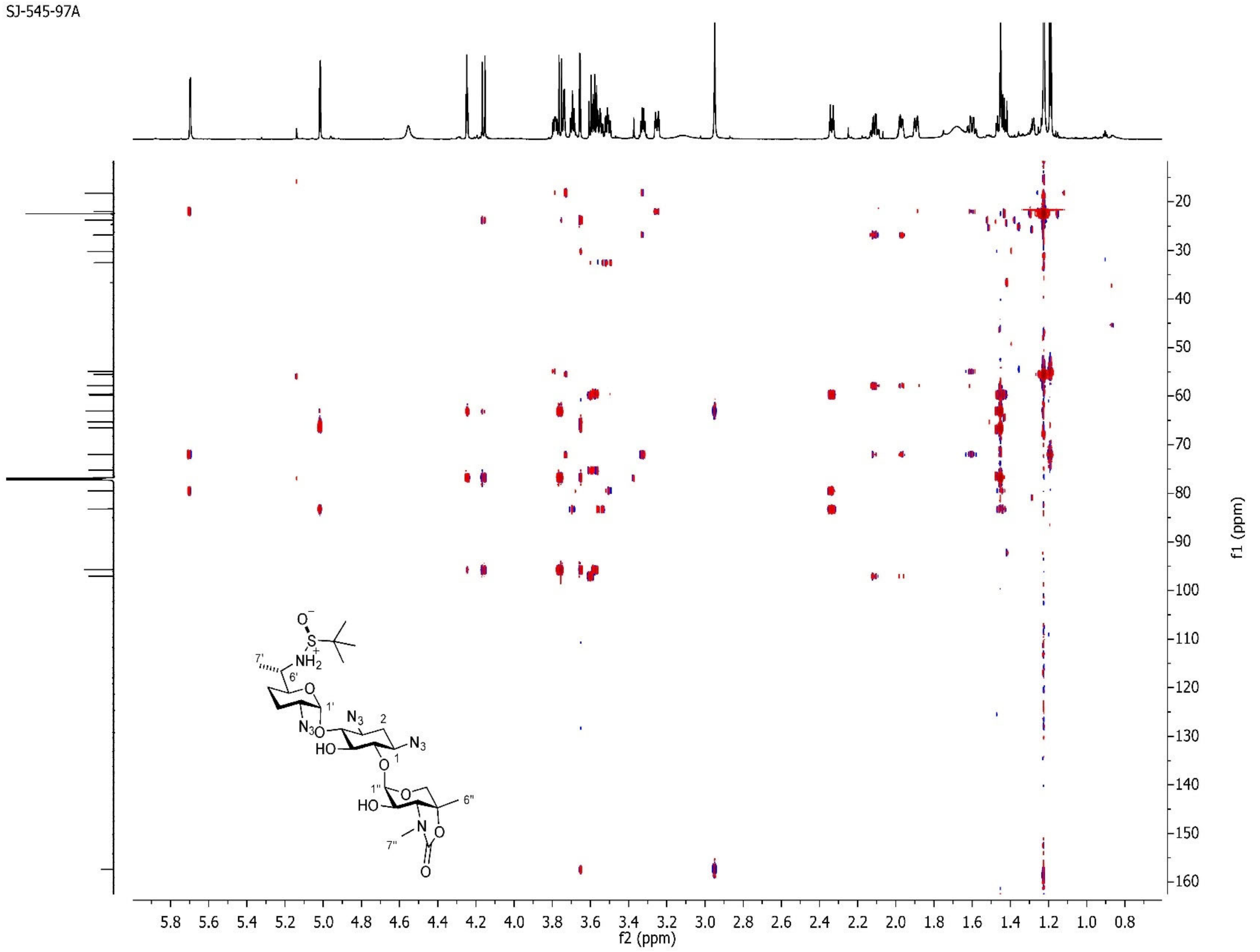

Gentamicin C2a (^{1}H 900 MHz, D_2O) (4):

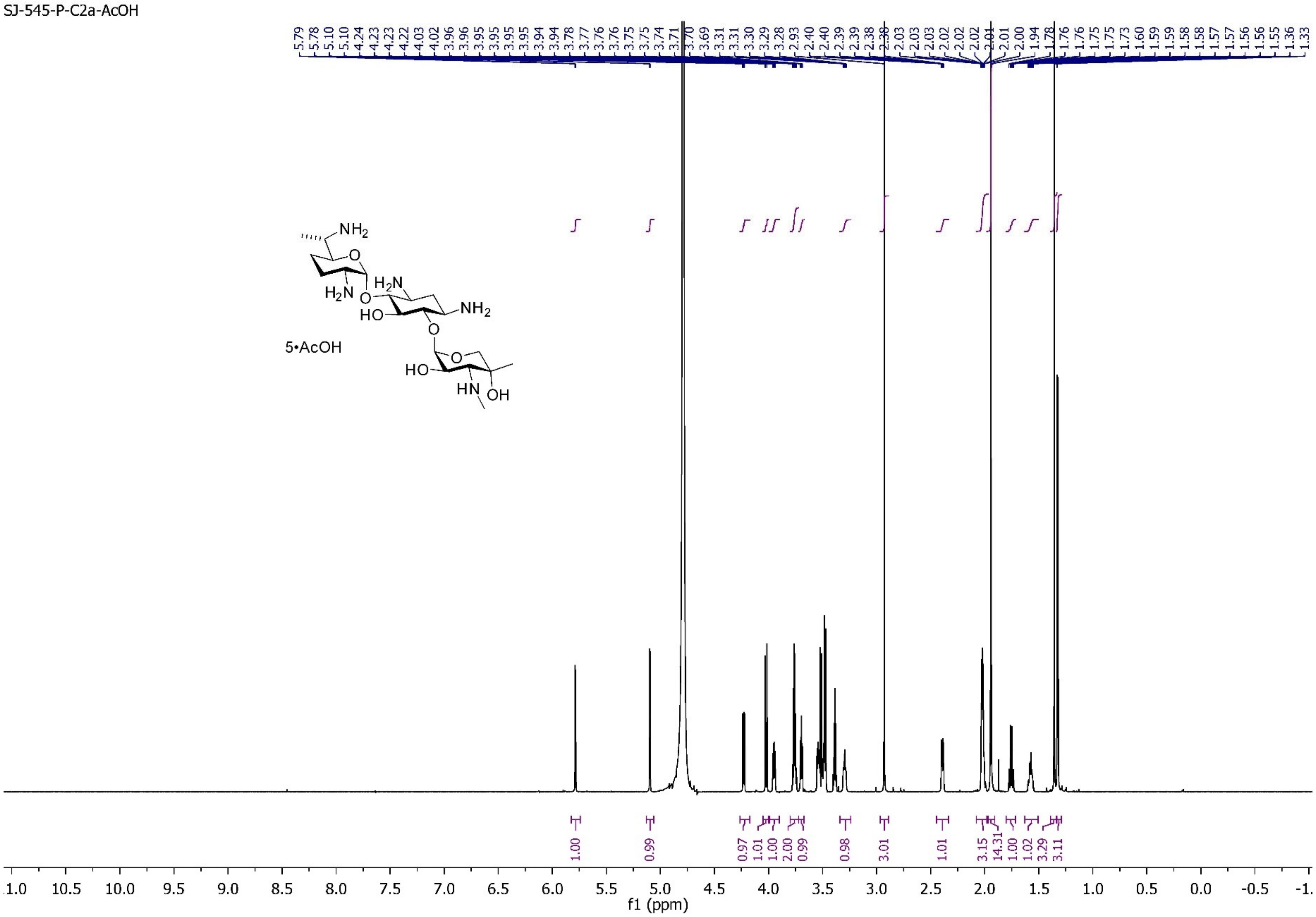

Gentamicin C2a (^{13}C 226 MHz, D_2O) (4):

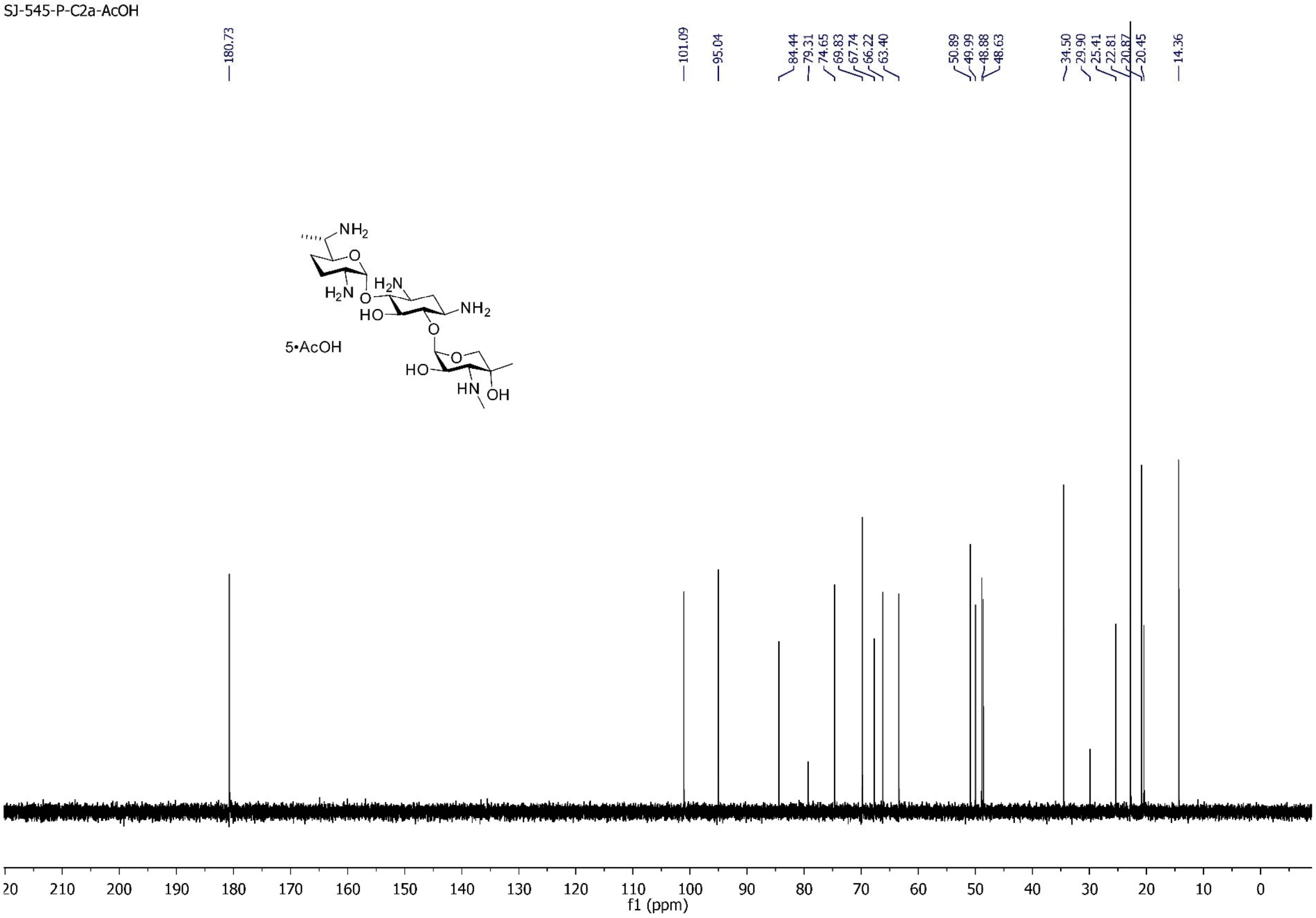

Gentamicin C2a (^{1}H-^{1}H-COSY 900 MHz, D_2O) (4):

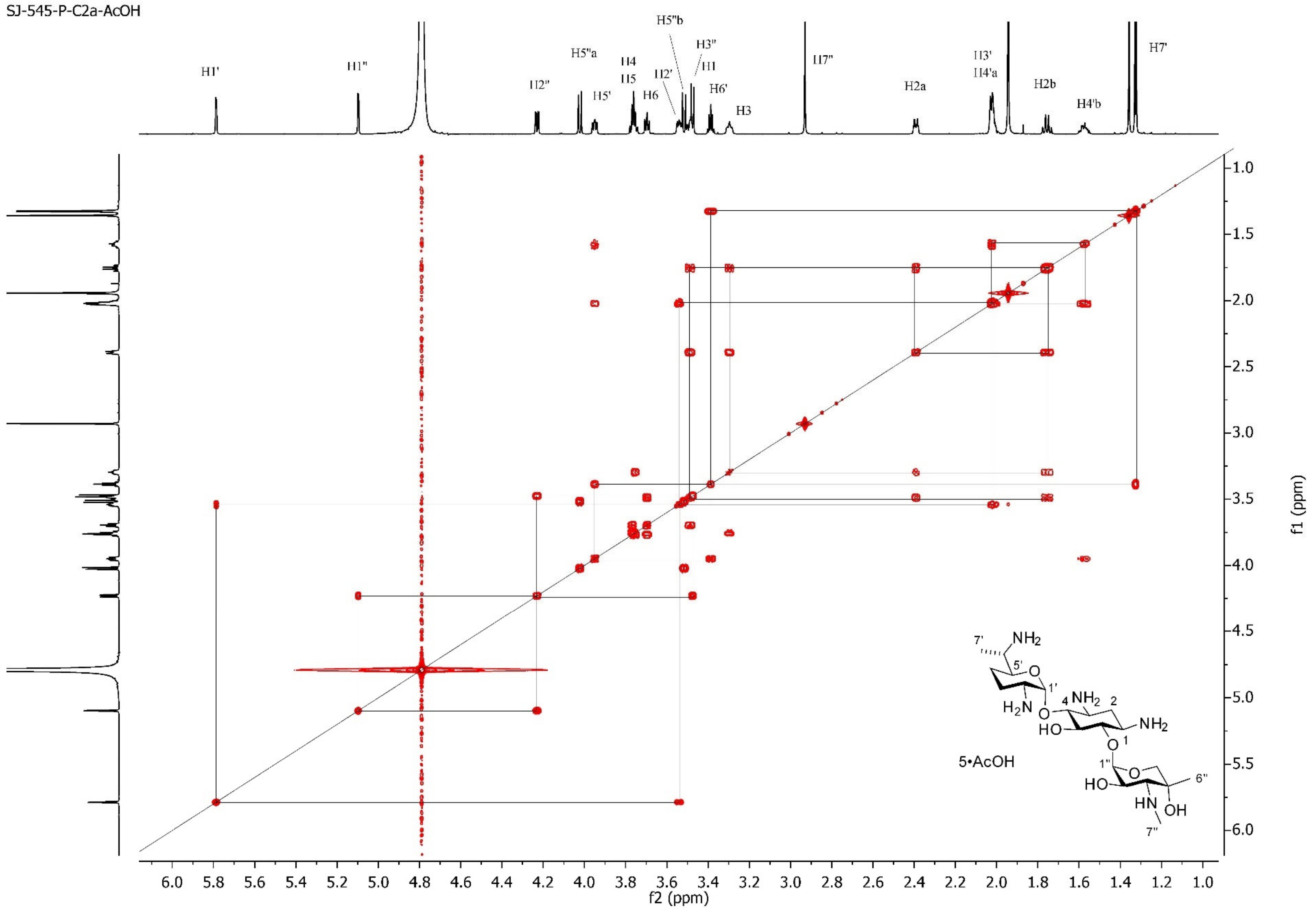

Gentamicin C2a (^1H-^{13}C-HSQC 900 MHz, D_2O) (4):

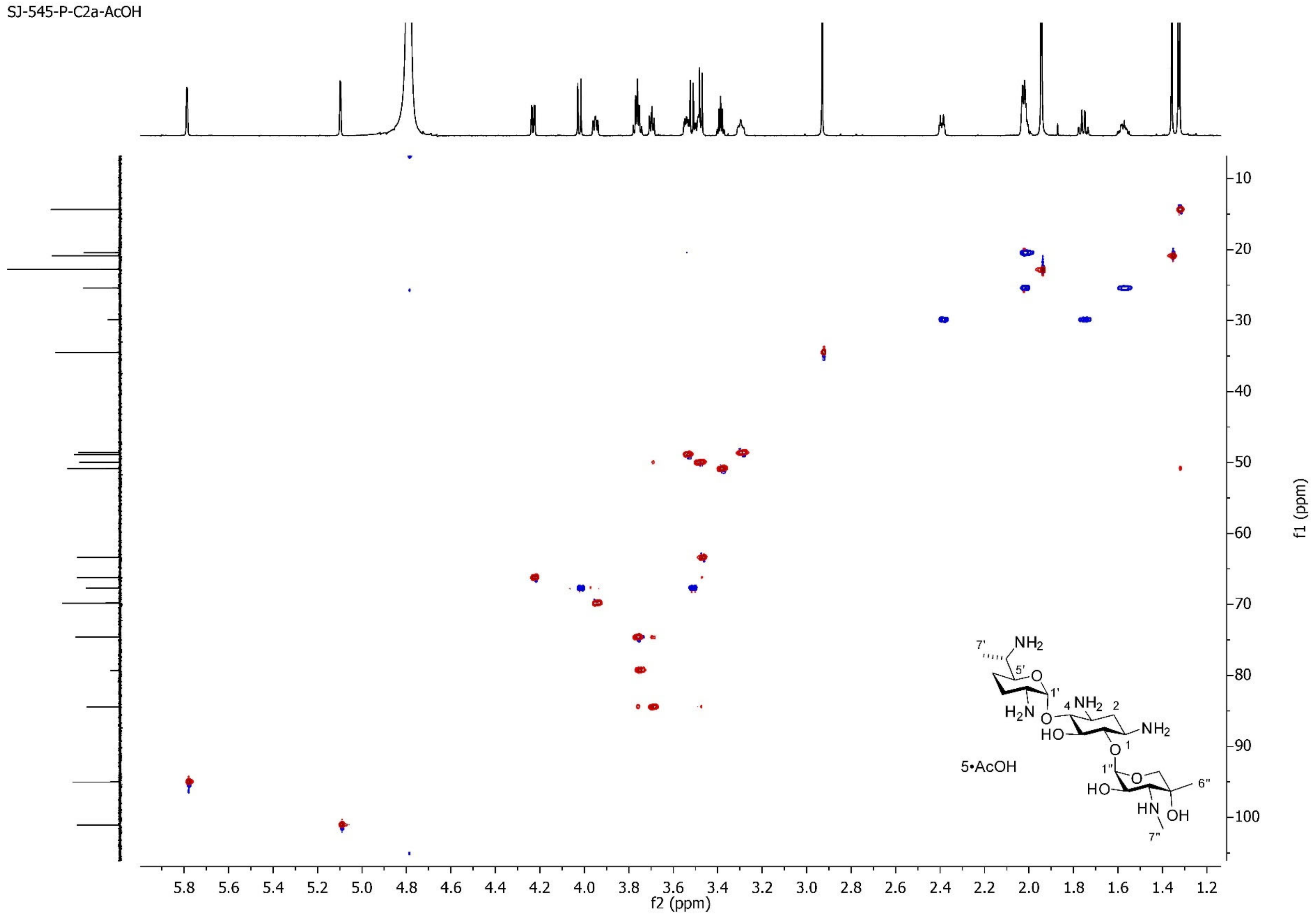

Gentamicin C2a (^{1}H-^{13}C-HMBC 900 MHz, D_2O) (4):

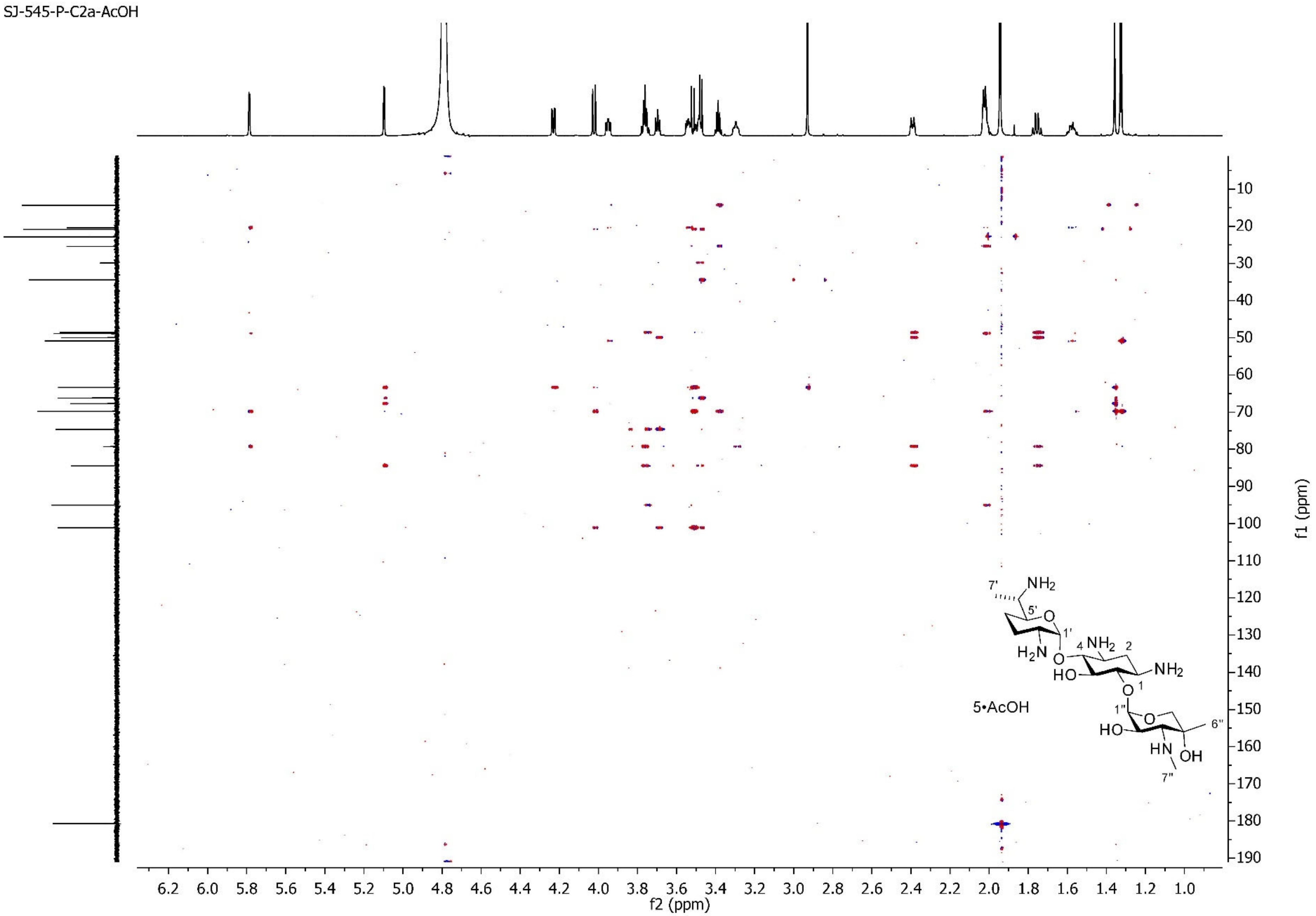

Gentamicin C2a (1D ROESY-H5′ 900 MHz, D_2O) (4):

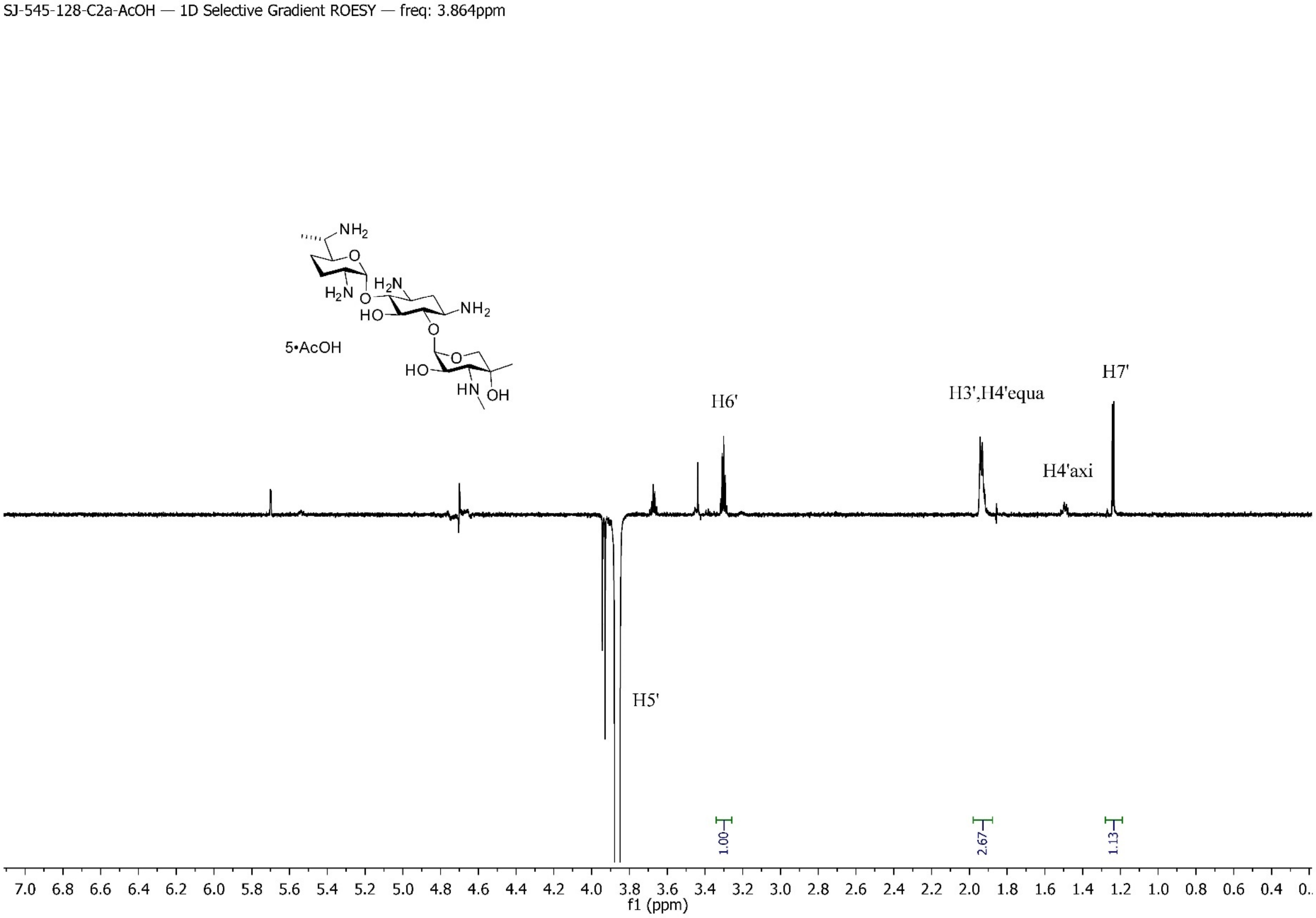

Gentamicin C2a (1D ROESY-H6′ 900 MHz, D_2O) (4):

SJ-545-128-C2a-AcOH — 1D Selective Gradient ROESY — freq: 3.301ppm

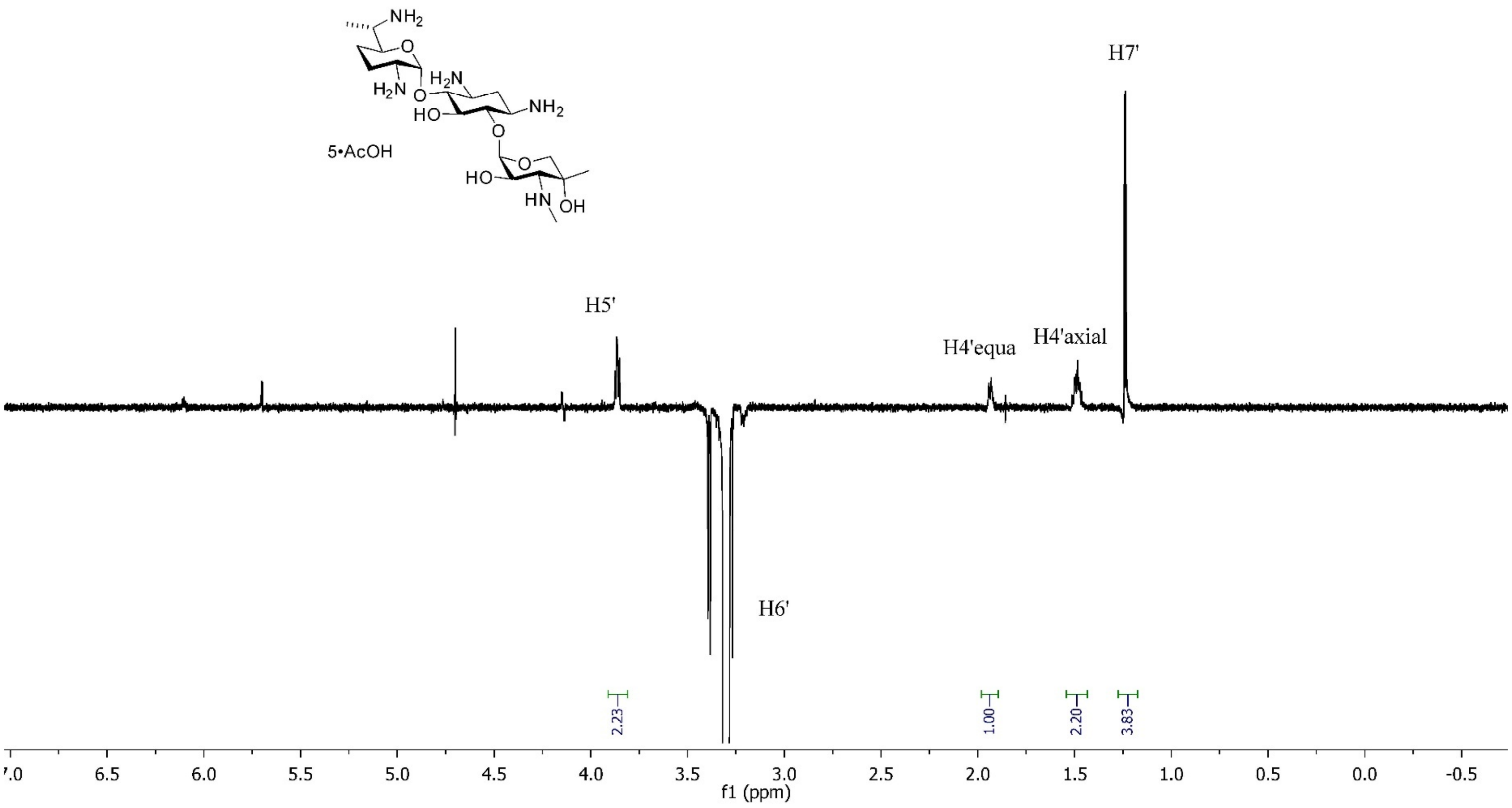

Gentamicin C2a (1D ROESY-H7′ 900 MHz, D_2O) (4):

SJ-545-128-C2a-AcOH — 1D Selective Gradient ROESY — freq: 1.239ppm

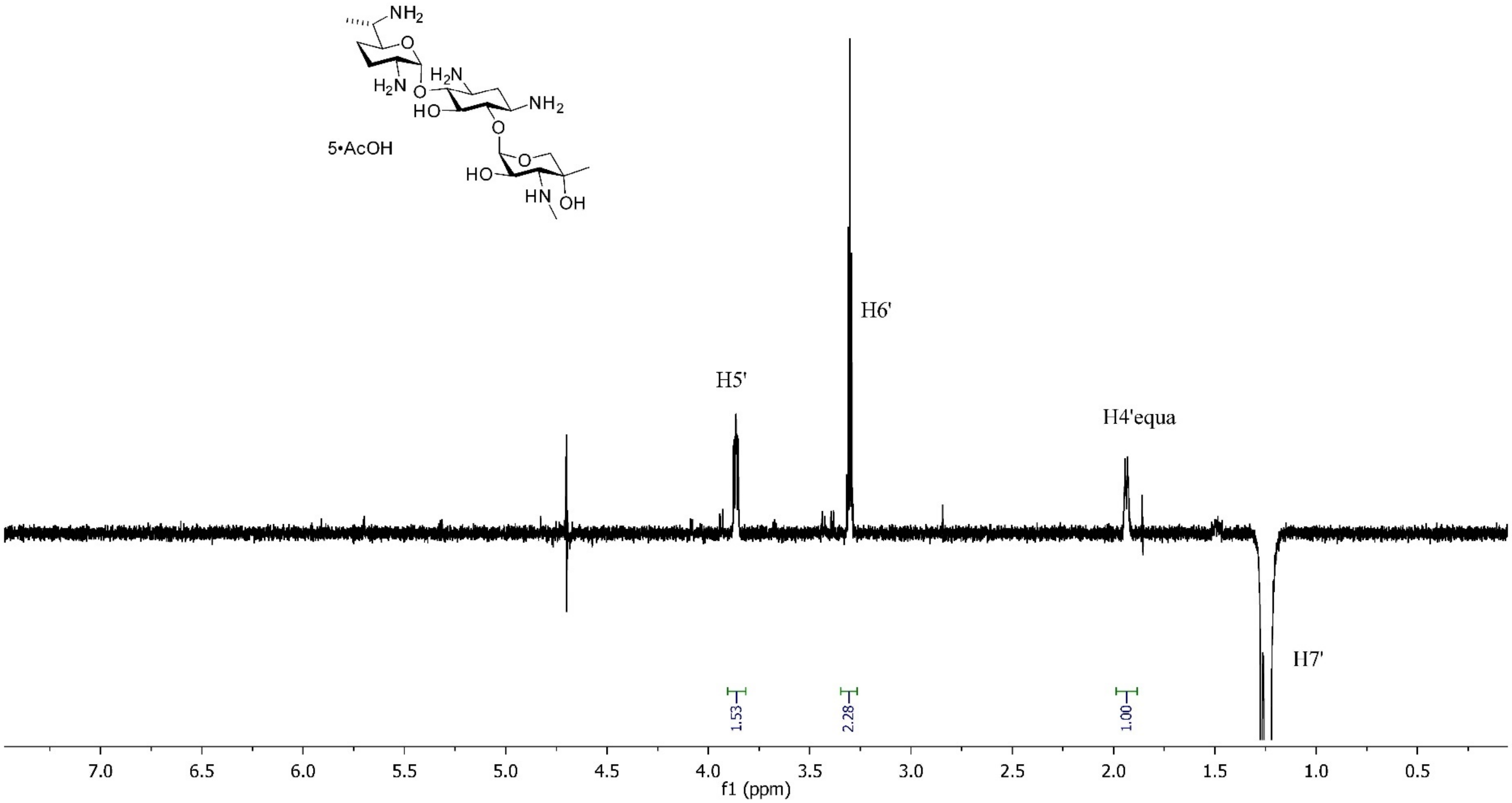

6′-*N*-Benzyl-1,3,2′-tri(deamino)-1,3,2′-triazido-3′′-*N*,4′′-*O*-carbonyl-gentamicin C1 (^{1}H 600 MHz, CD_3OD) (22):

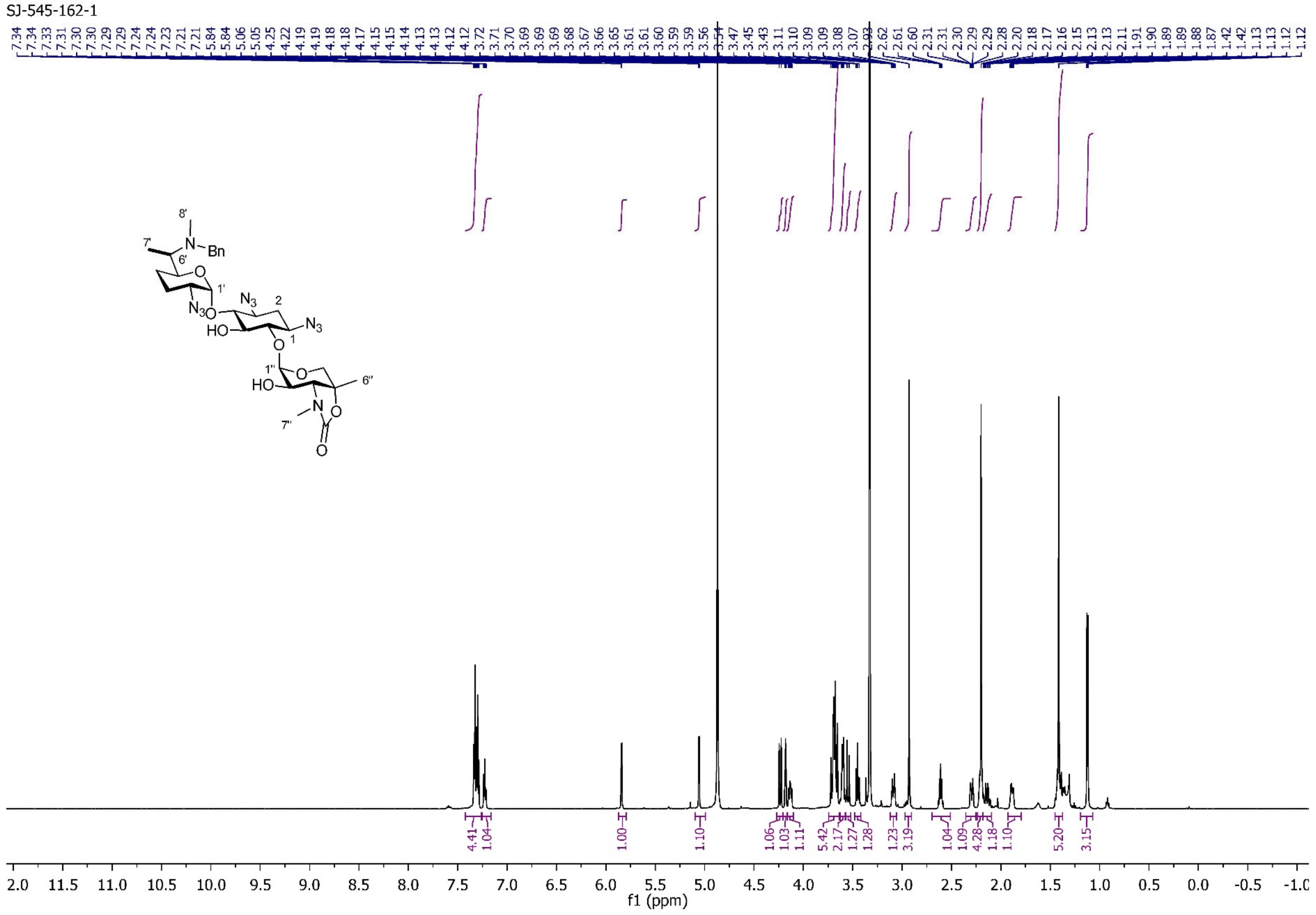

6′-*N*-Benzyl-1,3,2′-tri(deamino)-1,3,2′-triazido-3′′-*N*,4′′-*O*-carbonyl-gentamicin C1 (^{13}C 151 MHz, CD_3OD) (22):

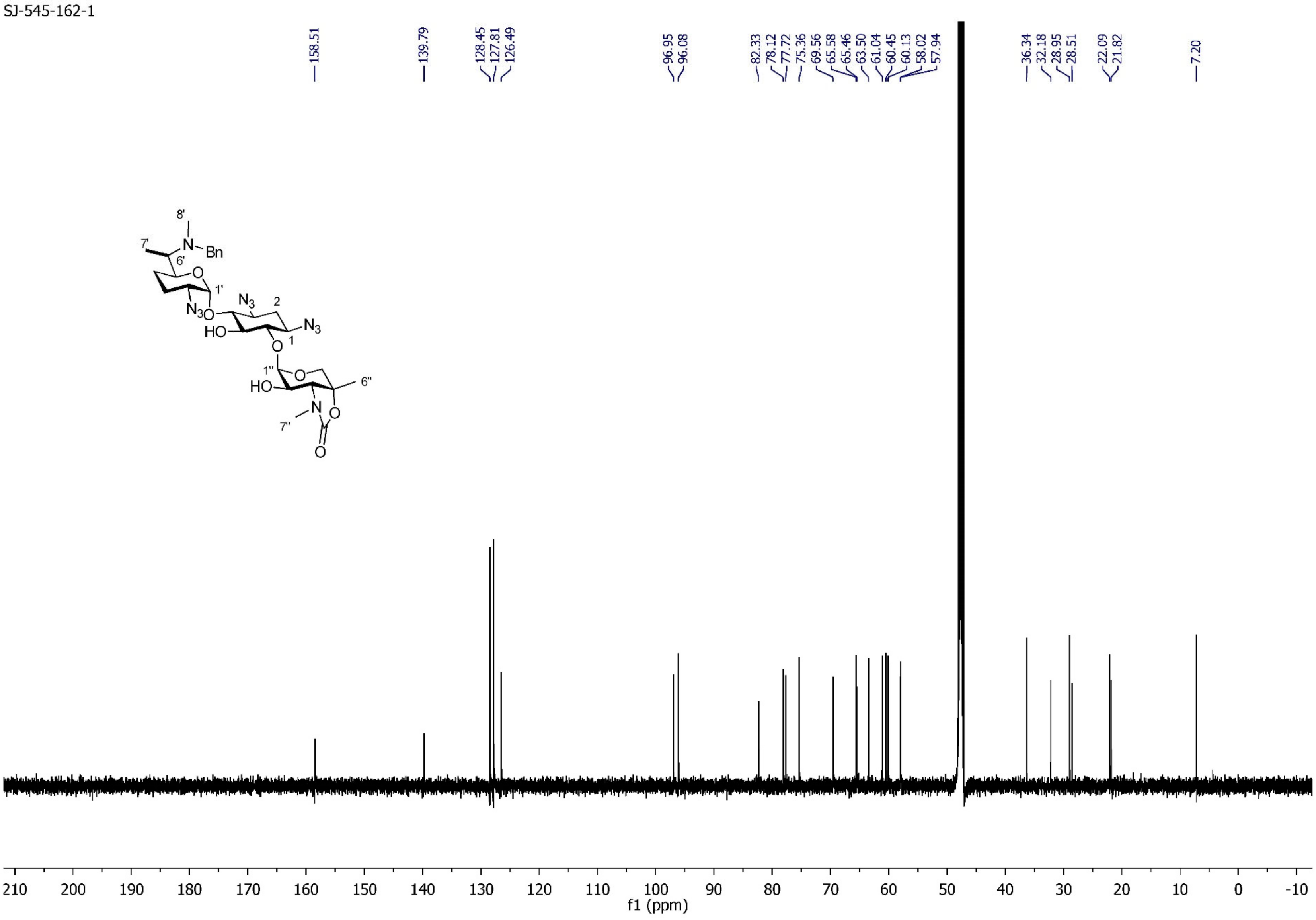

6′-*N*-Benzyl-1,3,2′-tri(deamino)-1,3,2′-triazido-3′′-*N*,4′′-*O*-carbonyl-gentamicin C1 (^{1}H-^{1}H-COSY 600 MHz, CD_3OD) (22):

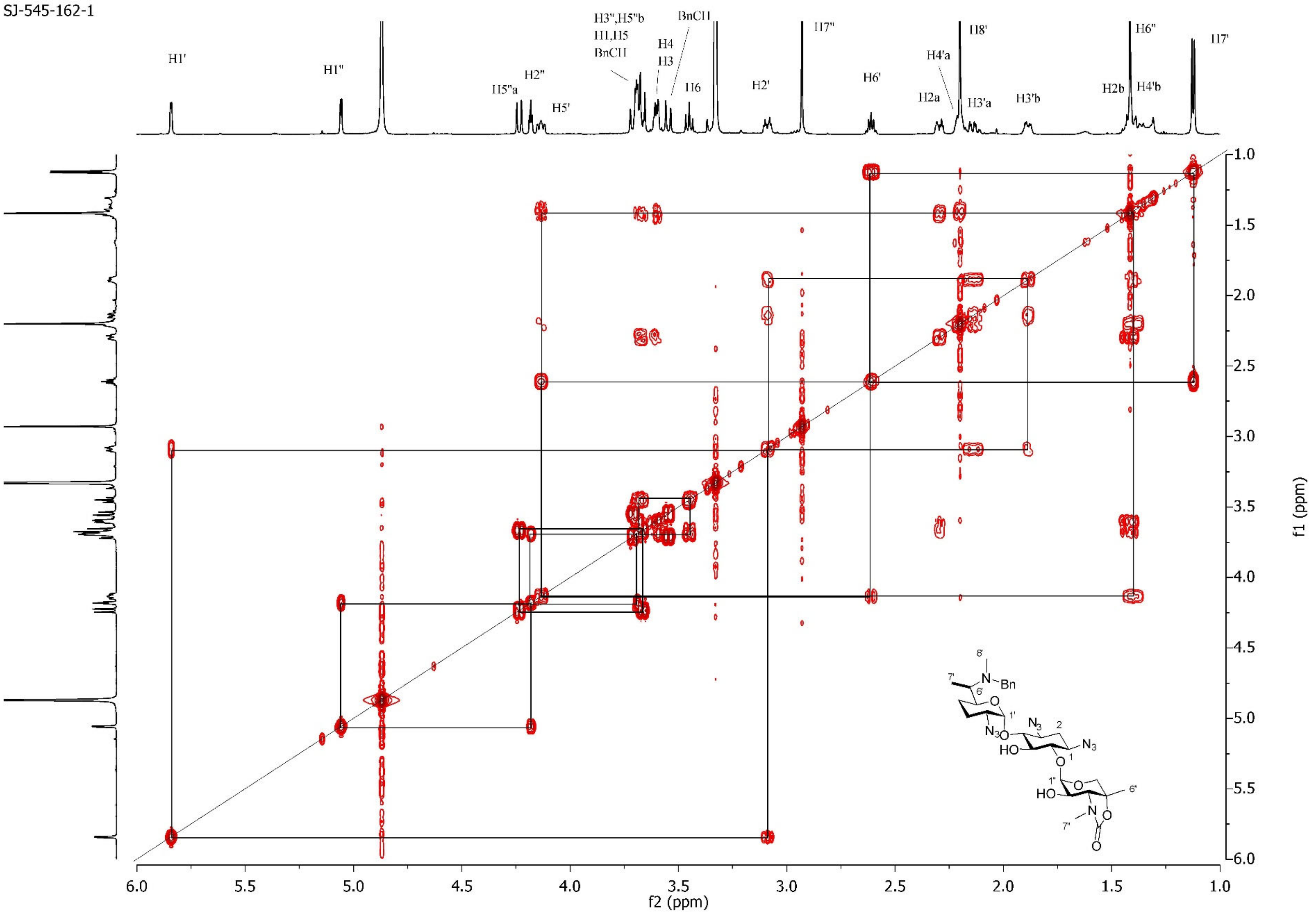

6′-*N*-Benzyl-1,3,2′-tri(deamino)-1,3,2′-triazido-3″-*N*,4″-*O*-carbonyl-gentamicin C1 (^1H-^{13}C-HSQC 600 MHz, CD_3OD) (22):

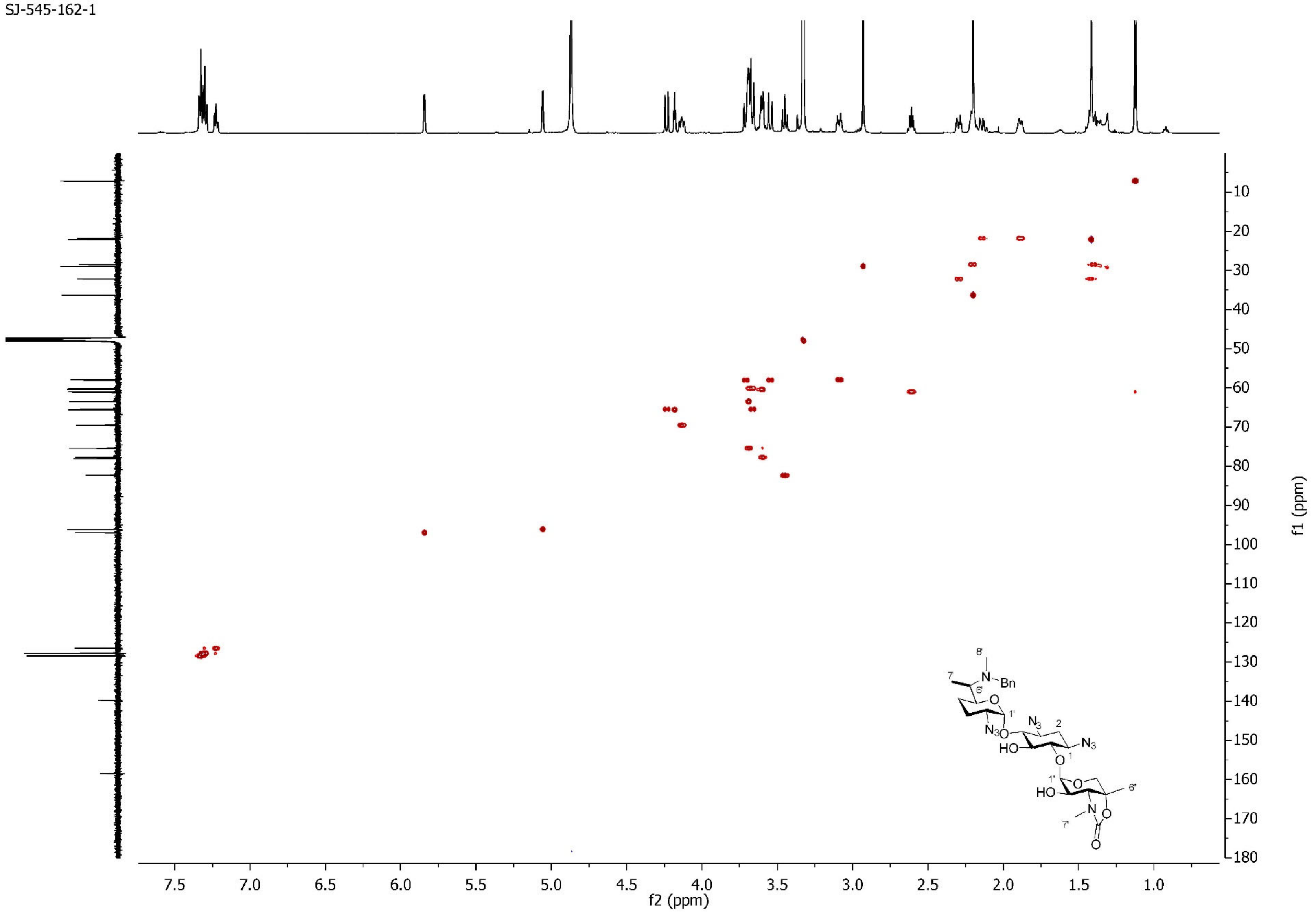

6′-*N*-Benzyl-1,3,2′-tri(deamino)-1,3,2′-triazido-3′′-*N*,4′′-*O*-carbonyl-gentamicin C1 (^{1}H-^{13}C-HMBC 600 MHz, CD_3OD) (22):

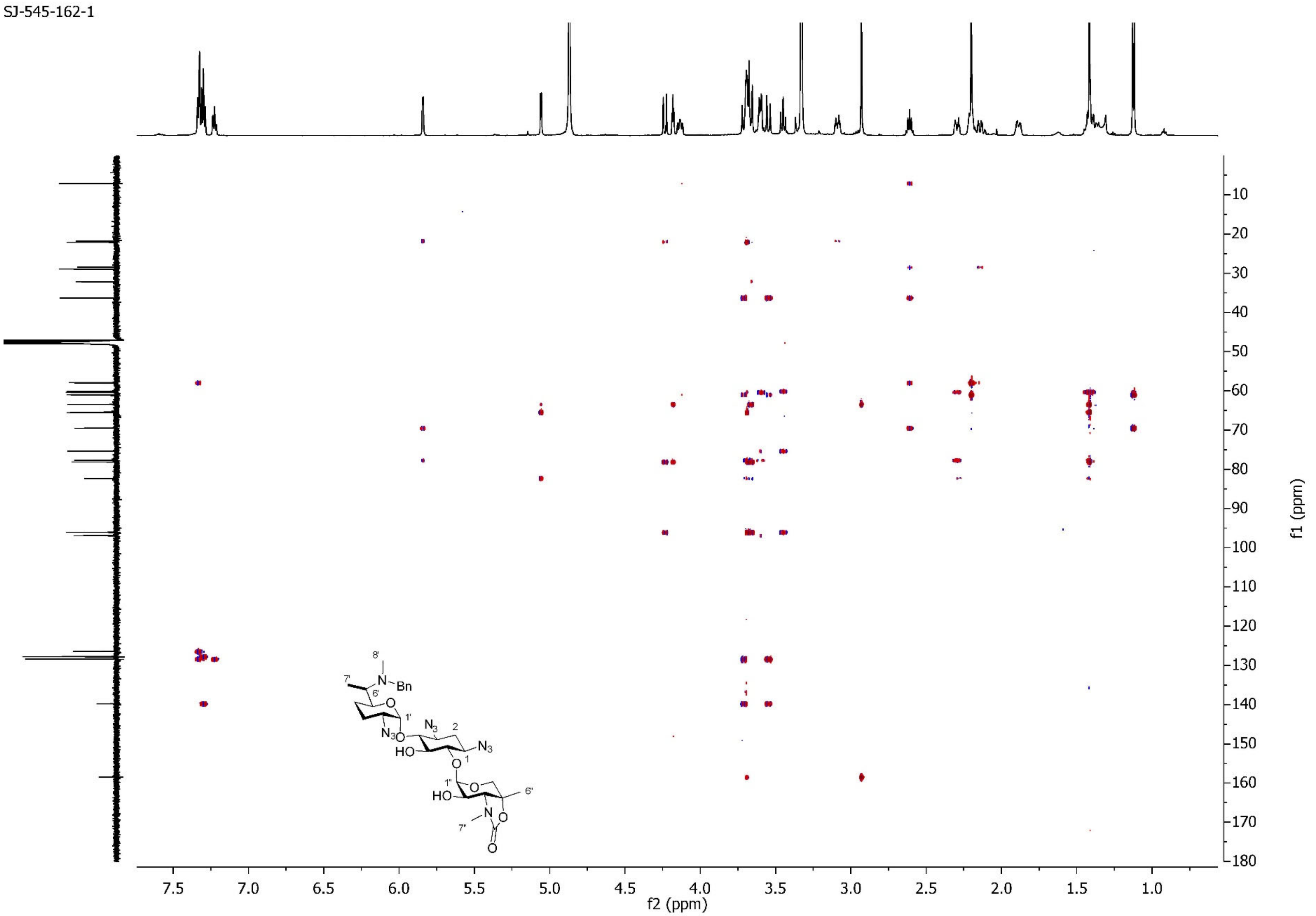

Gentamicin C1 (^{1}H 900 MHz, D_2O) (1):

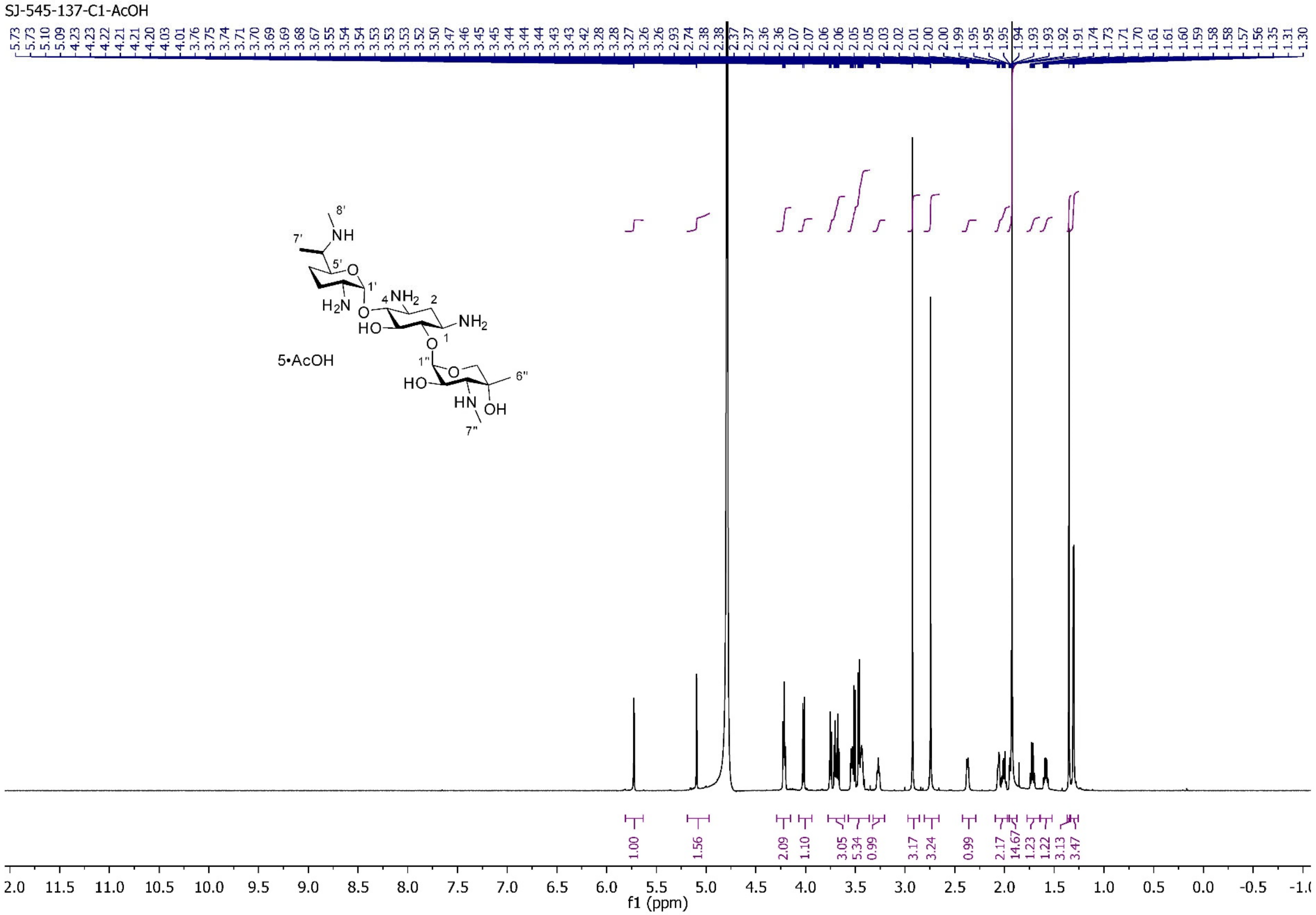

Gentamicin C1 (^{13}C 226 MHz, D_2O) (1):

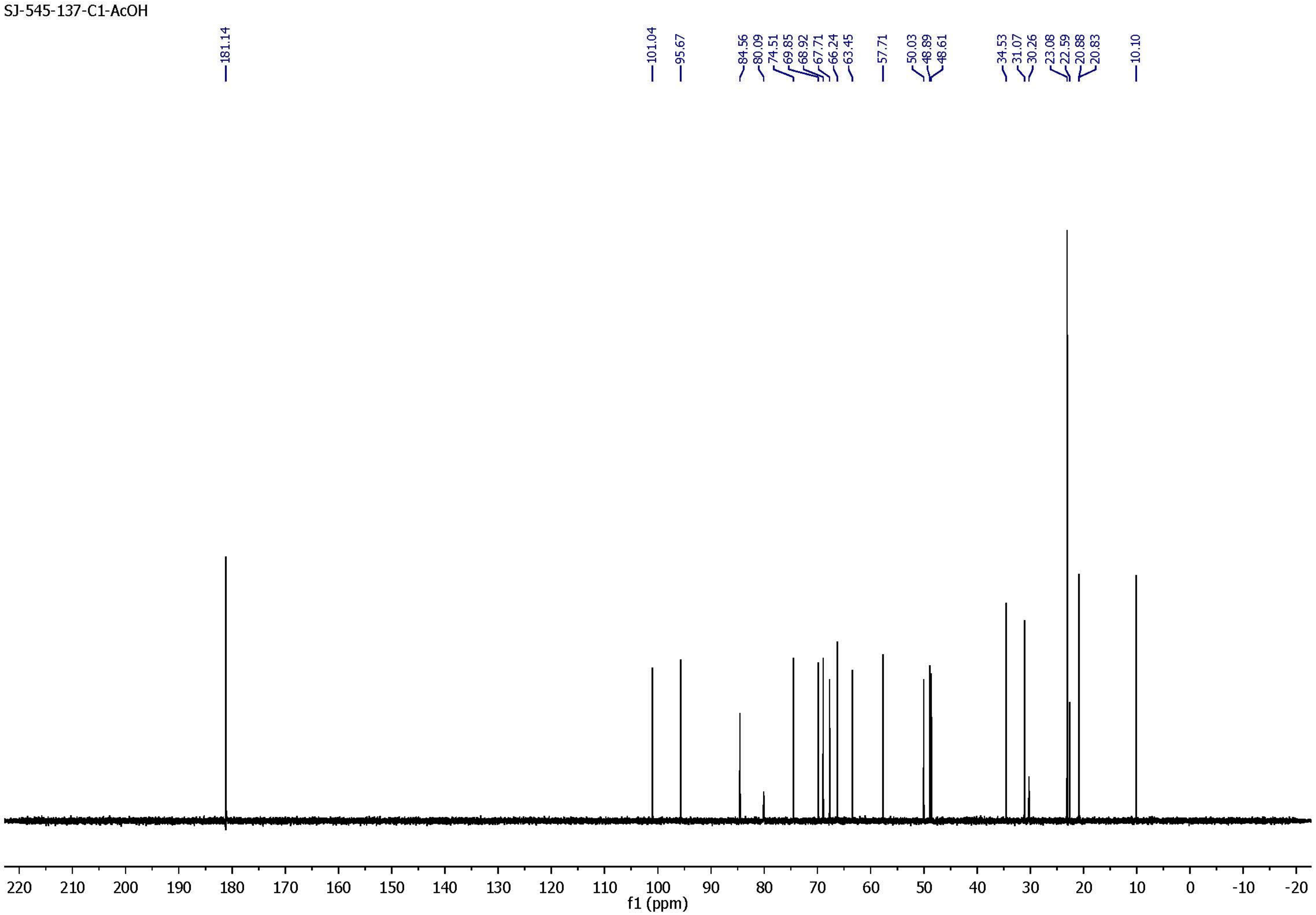

Gentamicin C1 (^{1}H-^{1}H-COSY 900 MHz, D_2O) (1):

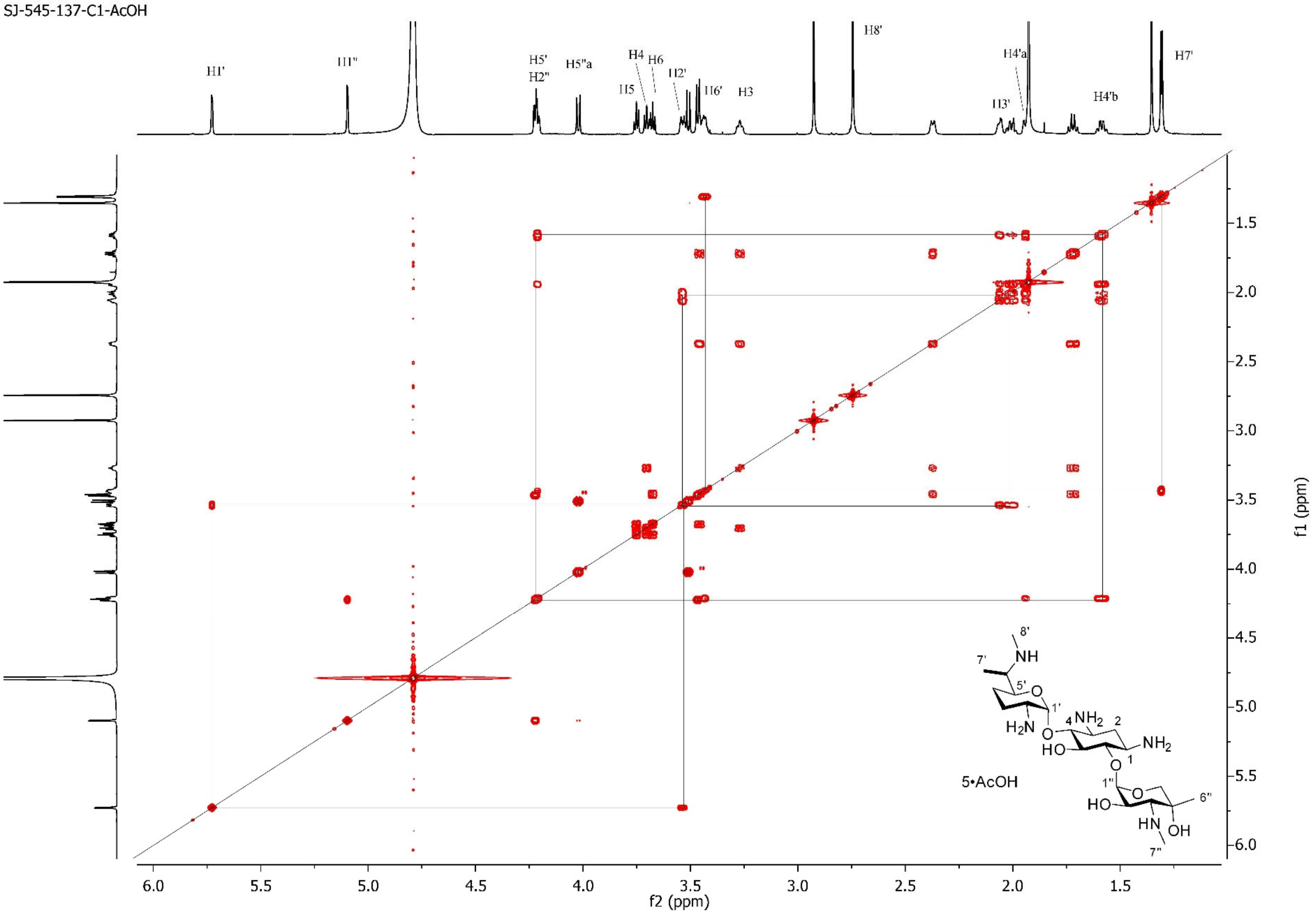

Gentamicin C1 (^{1}H-^{13}C-HSQC 900 MHz, D_2O) (1):

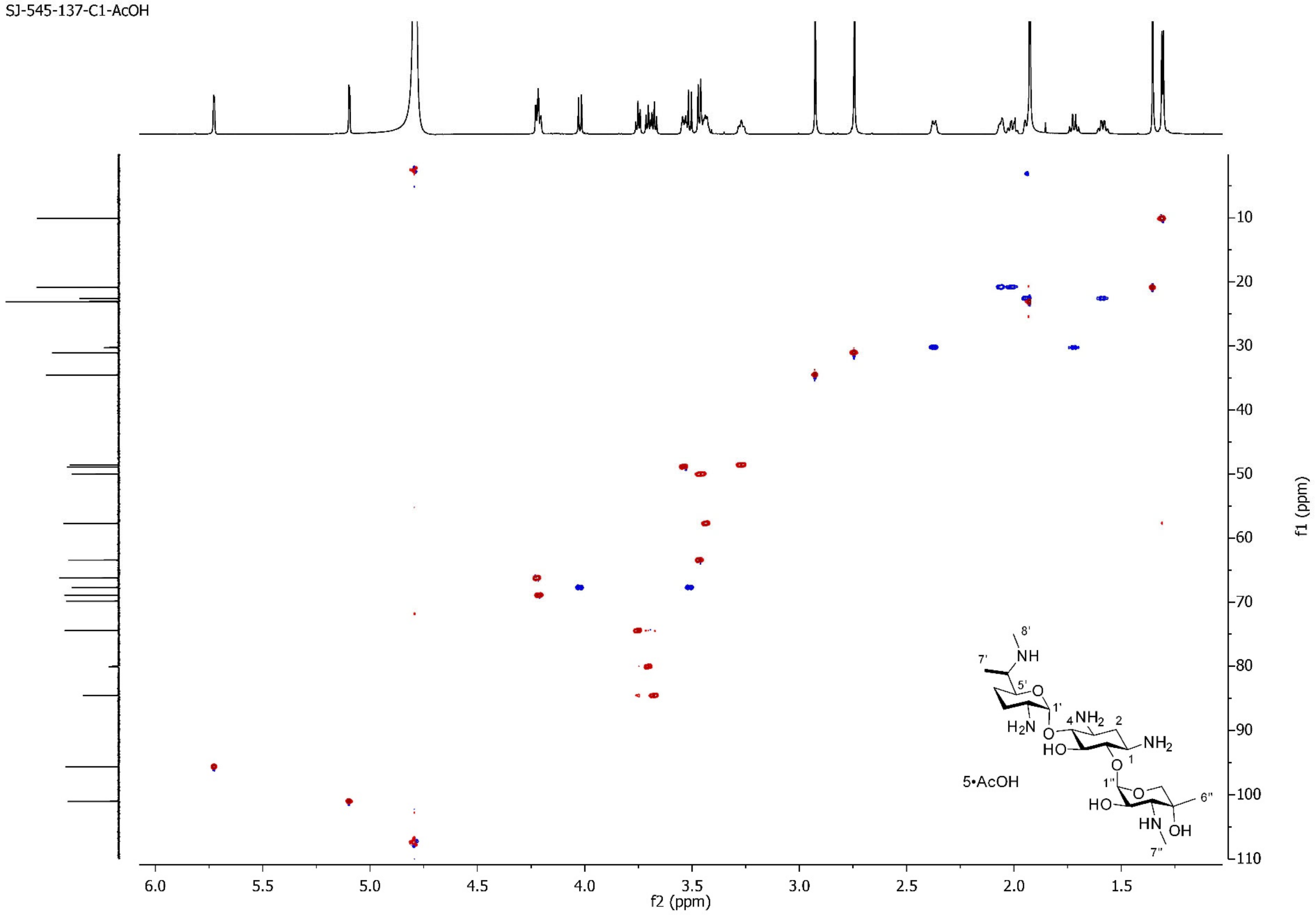

Gentamicin C1 (^1H-^{13}C-HMBC 900 MHz, D_2O) (1):

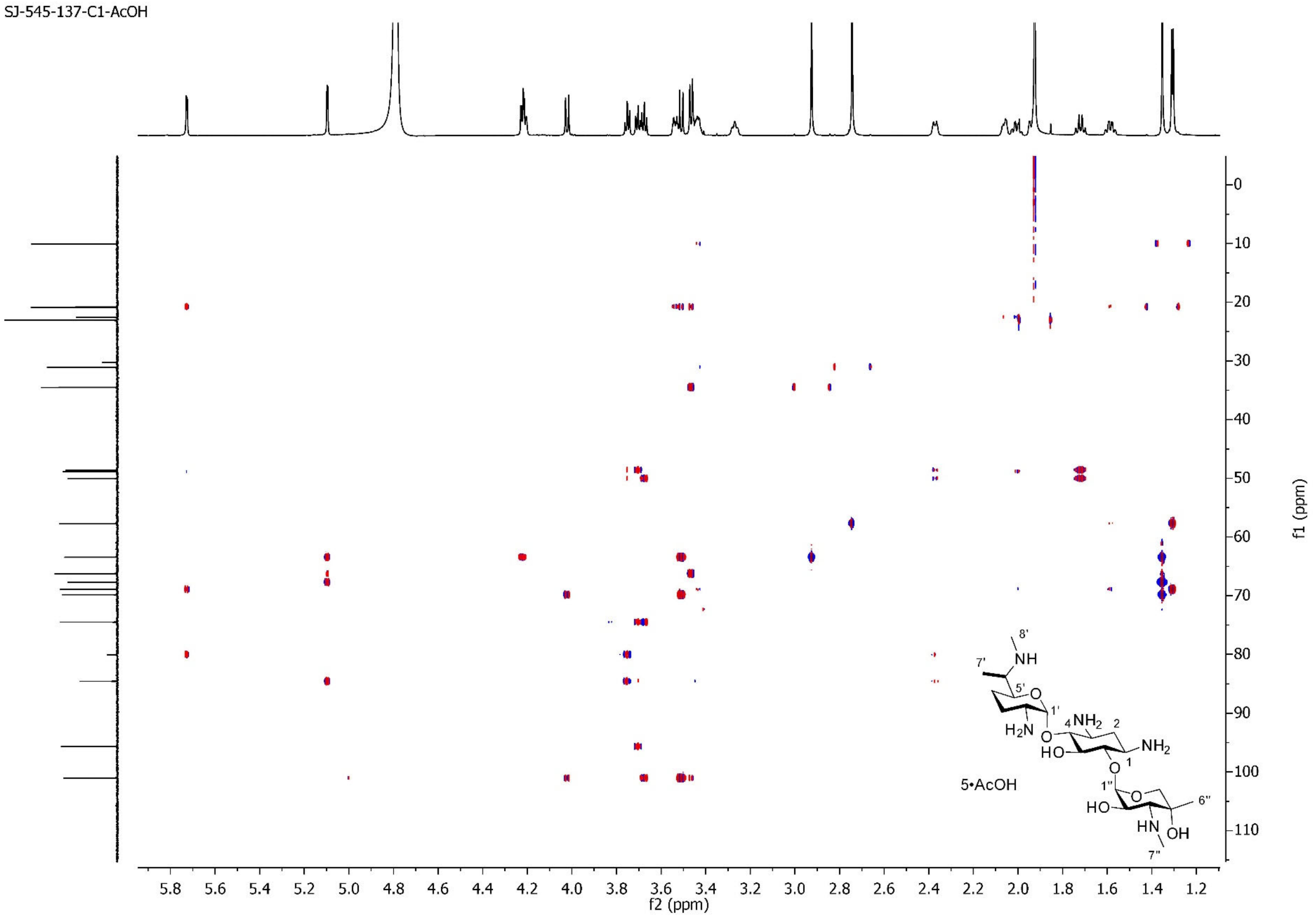

Supplement: Supplementary file 1 — id3c00233_si_001.pdf [file id3c00233_si_001.pdf]
